# Large-scale mining of plant genomes unlocks the diversity of oxidosqualene cyclases

**DOI:** 10.1038/s41589-025-02034-8

**Published:** 2025-10-06

**Authors:** Michael J. Stephenson, Charlotte Owen, James Reed, Anne Osbourn

**Affiliations:** 1 Department of Biochemistry and Metabolism, John Innes Centre, Norwich Research Park, Norwich, UK.

## Abstract

The differential cyclization and rearrangement of 2,3-oxidosqualene controlled by oxidosqualene cyclases (OSCs) represents one of the most complex single enzyme transformations in nature and gives rise to a vast array of triterpenoid diversity in the plant kingdom. Here we systematically mine 599 plant genomes representing 387 species and investigate OSC diversity across different plant lineages. From the OSC sequences identified, 20 were selected for functional evaluation. Through analysis of these enzymes, we discover product profiles within clades previously believed to be functionally conserved and OSCs producing triterpenes for which no enzymatic source was known. We also discover OSCs with product profiles that yield mechanistic insights into the control of specific reaction pathways. Our study reveals lineage-specific blooms of OSC subgroups suggestive of adaptation to different environmental niches, opens up previously inaccessible chemistry and provides a framework for systematic investigations of metabolic diversification and underlying enzymatic mechanisms in the plant kingdom.

The terpenoids are among the largest classes of plant natural products, with >80,000 reported to date^1^. Their scaffolds are biosynthesized from linear substates containing multiple five-carbon (C5) isoprene units to give cyclized molecules of varying complexity, for example monoterpenes (C10), sesquiterpenes (C15), diterpenes (C20) and triterpenes (C30). The triterpenoids are the most diverse and structurally complex of all of the terpenoids^2^. These compounds have many important functions in plants, providing protection against pests and pathogens, shaping the root microbiome and influencing crop quality^3,4^. They are also precursors to essential steroid membrane components and hormones and serve as scaffolds for the biosynthesis of steroidal specialized metabolites (for example, steroidal glycoalkaloids)^5^. The triterpenoids are a particularly rich source of bioactive molecules and are of considerable commercial interest for medicinal and other uses^3,6^. Examples include the vaccine adjuvant QS-21 produced by the Chilean soapbark tree *Quillaja saponaria*^7,8^, the anti-inflammatory compound escin from horse chestnut^9,10^ and bee-friendly insecticidal limonoids produced by neem (*Azadiracta indica*) and other members of the Meliaceae family^11^.

Over 20,000 triterpenoids have been reported so far from nature^2^, the vast majority of these from plants. The triterpene scaffolds that form the basis for this plethora of plant triterpenoids are generated from a single linear C30 precursor 2,3-oxidosqualene, by a family of enzymes known as oxidosqualene cyclases (OSCs). Collectively, OSCs are capable of cyclizing 2,3-oxidosqualene into an array of diverse triterpene scaffolds (>200 reported so far) through a complex origami-like process involving sequential carbocation cascades (Fig. 1)^5,12,13^. These scaffolds can then be modified by cytochromes P450 and other tailoring enzymes (for example glycosyltransferases and acyltransferases) to give enormous structural diversity^2,6^. Around 170 plant OSCs have been functionally characterized to date, collectively accounting for ~60 of the >200 triterpene scaffolds represented in the natural product databases^14^. The OSCs for the remaining ~140 reported triterpene scaffolds (orphan scaffolds) are as yet unknown. The amount of triterpene scaffold diversity represented in plants is in reality likely to be much greater than this, as a number of examples of OSCs that make entirely new triterpene scaffolds have recently been reported, even for well-characterized species such as rice^15,16^.

The conversion of 2,3-oxidosqualene to different triterpene scaffolds is one of the most complex enzymatic transformations observed in nature and has fascinated chemists for the best part of a century. In the 1950s, attempts to mechanistically rationalize the stereochemical divergence of two constitutionally identical tetracyclic triterpenes, lanosterol and euphol, led to the formulation of the biogenic isoprene rule^17,18^. This rule postulated that the stereoselectivity of initial ring-forming electrophilic additions is controlled through the prior conformational folding of the substrate to either a chair–boat–chair (CBC) or chair–chair–chair (CCC) arrangement with respect to the A, B and C rings (Fig. 1a). Subsequent formation of the D ring would result in two different tetracyclic cation intermediates, now termed the protosteryl and dammarenyl cations, respectively. Resolution of these two cations through an analogous series of 1,2-hydride and 1,2-alkyl shifts terminating in an elimination yields the neutral alkenes, lanosterol (from the protosteryl cation) and euphol (from the dammarenyl cation) (Fig. 1a). The protosteryl cation can in addition give rise to other CBC conformation products such as parkeol, cycloartenol and cucurbitadienol (Fig. 1a), while the dammarenyl cation is the gateway to a huge array of CCC conformation products that in turn form the basis of a swathe of plant specialized triterpenes (Fig. 1b). The paradigm that all triterpenes comply with this stereochemical conformity and can be rationalized mechanistically as originating from one of these two common intermediates is not, however, absolute. For example, the recent discovery of a divergent OSC from rice that produces a pentacyclic triterpene (orysatinol), the stereochemistry of which cannot be reasoned to originate from either the protosteryl or dammarenyl cation, provides an exception to this rule^15,16^. Although multiple amino acid residues have been shown by mutational analysis to be involved in OSC function^14,19,20^, the features determining product specificity and mechanisms of triterpene cyclization are not well understood.

It is clear that the natural products reported from plants to date represent only the tip of the iceberg, as sequencing has revealed that around 20% of the genes within plant genomes are predicted to have yet-uncharacterized functions in specialized metabolism^21^. The amount of available plant genome sequence data has expanded rapidly over the last 10 years and is set to explode with the advent of large-scale sequencing initiatives such as the Earth BioGenome Project^22^. This opens up opportunities to investigate the potential of plant genomes to encode enzymes that make different chemistry through systematic genome mining and functional analysis of OSCs, to understand the mechanisms of triterpene cyclization and to reveal how the ability to synthesize different types of triterpenes has arisen in different plant lineages suggestive of adaptation to environmental niches.

There are currently over 4,800 land plant species represented in the National Center for Biotechnology Information (NCBI) genome database. However, fewer than 15% of these genomes have been submitted with accompanying gene annotation data (www.ncbi.nlm.nih.gov/genome/). In contrast to microbes, predicting accurate gene models without transcriptome mapping in plants is nontrivial because of variability in genome size and complexity, splice site frequency, intron size and intergenic distances^23–25^. In this study, we use the highly accurate and easy to use Selenoprofiles^26^ in a sequential pipeline with PSI-tBLASTn^27^, Exonerate^28^ and GeneWise^29^ as a targeted approach for the identification of putative OSC gene models from unannotated plant genome sequences.

## Results

### Mining OSC sequence diversity across the plant kingdom

**Extended Data Fig. 1:**
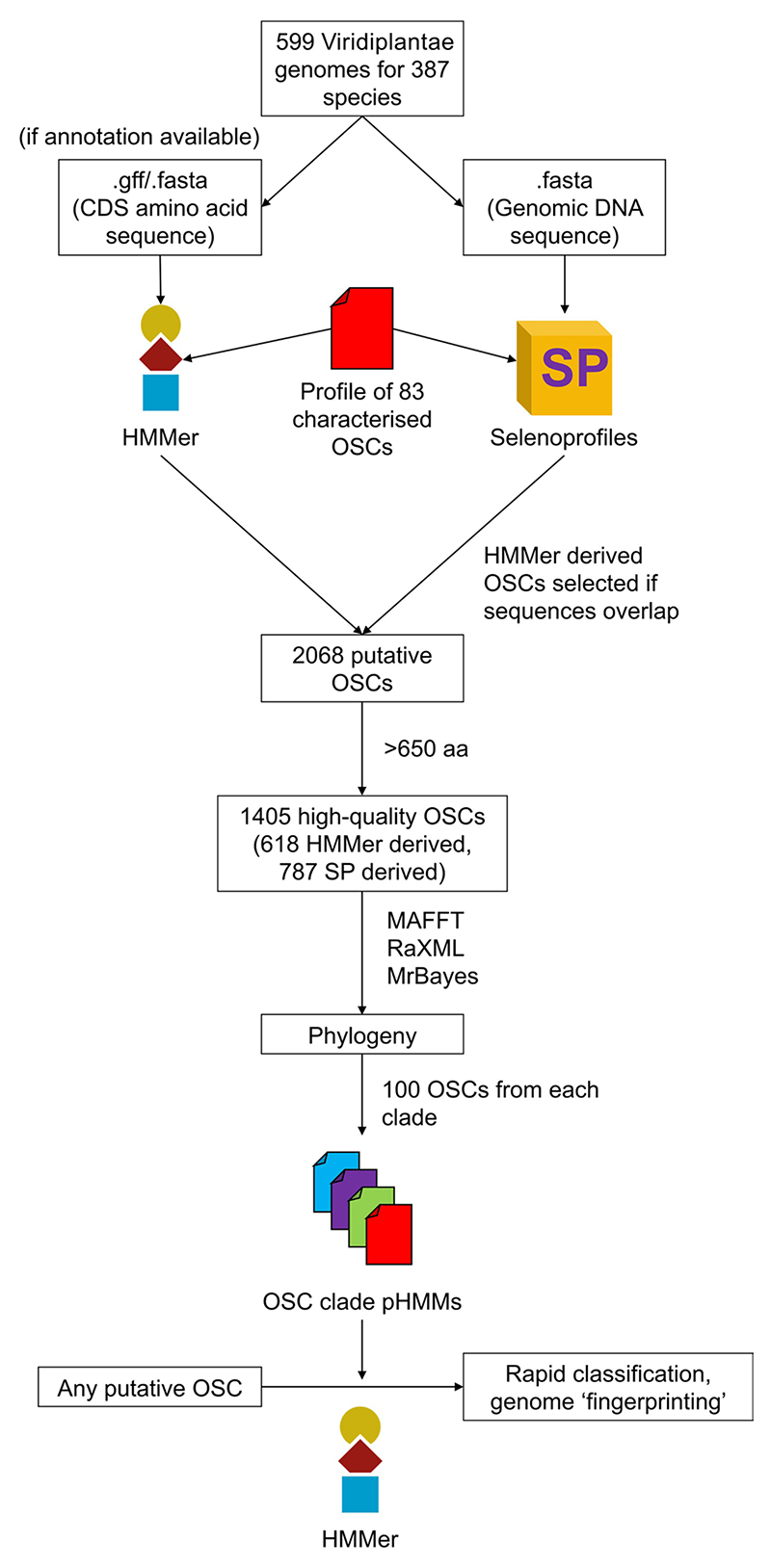
Summary of computational workflow for high-throughput, systematic OSC mining from plant genomes of varying annotation quality. HMMER^50^ and Selenoprofiles^26^ were used in conjunction with characterised OSCs in order to fully utilise the available plant genome sequence data, despite the absence or low quality of genome annotations. After filtering the discovered OSCs to ensure only high-quality sequences were assessed, a phylogenetic analysis was carried out, which was then used to define distinct OSC groups and pHMM generation for on-the-fly OSC characterisation.

We used a total of 599 plant genome sequences of varying annotation quality representing 387 species within the Viridiplantae (Supplementary Data 1). The order-level taxonomy of the plant species used is shown in Supplementary Fig. 1, with species with multiple genomes counted as one. The computational workflow for high-throughput systematic mining for OSC genes is shown in Extended Data Fig. 1. A total of 2,068 unique, nonoverlapping predicted OSC genes were identified from genomes of 258 species. These were filtered on the basis of sequence length and gene model confidence to give 1,405 high-quality OSC sequences (Methods), of which 809 sequences were derived from unannotated genome data using Selenoprofiles.

**Extended Data Fig. 2:**
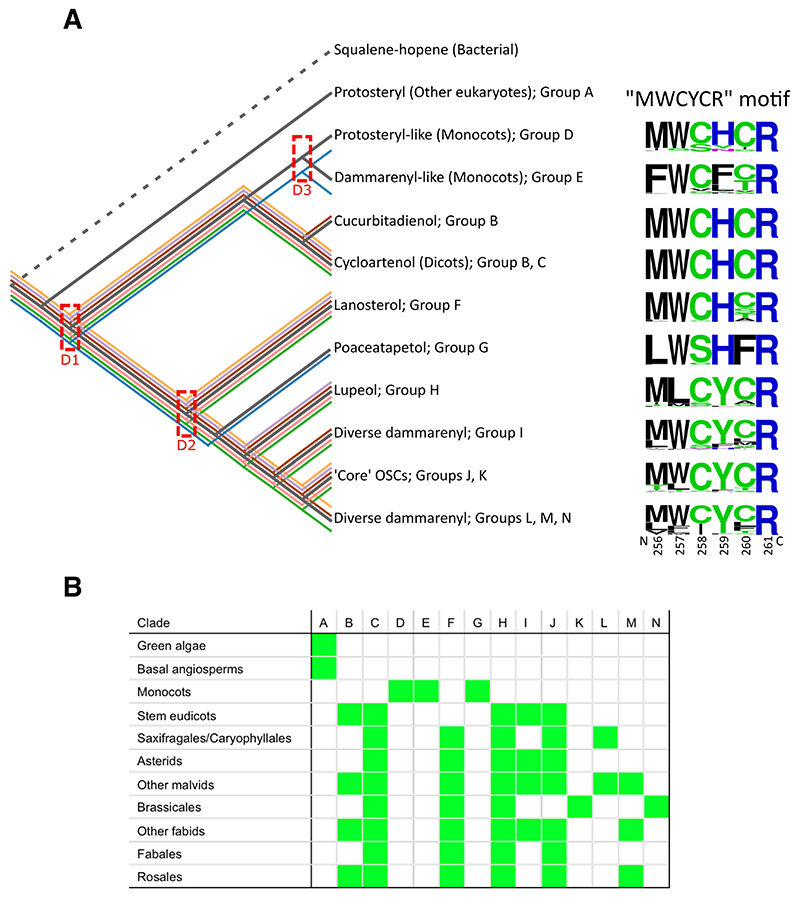
Diversity and distribution of OSCs across plant clades. **a**, Cladogram summarizing OSC evolutionary pathways across plant clades, with D1-3 labelled. Branch colours are as used in Fig. 2. The evolutionary origin of eukaryotic OSCs, unlike other terpene synthases, is shared with the bacterial squalene-hopene synthases (dotted line). Conserved residues from the ‘MWCYCR’ motif are shown^5^. **b**, Absence/presence of OSC groups in different plant clades.

**Fig. 2:**
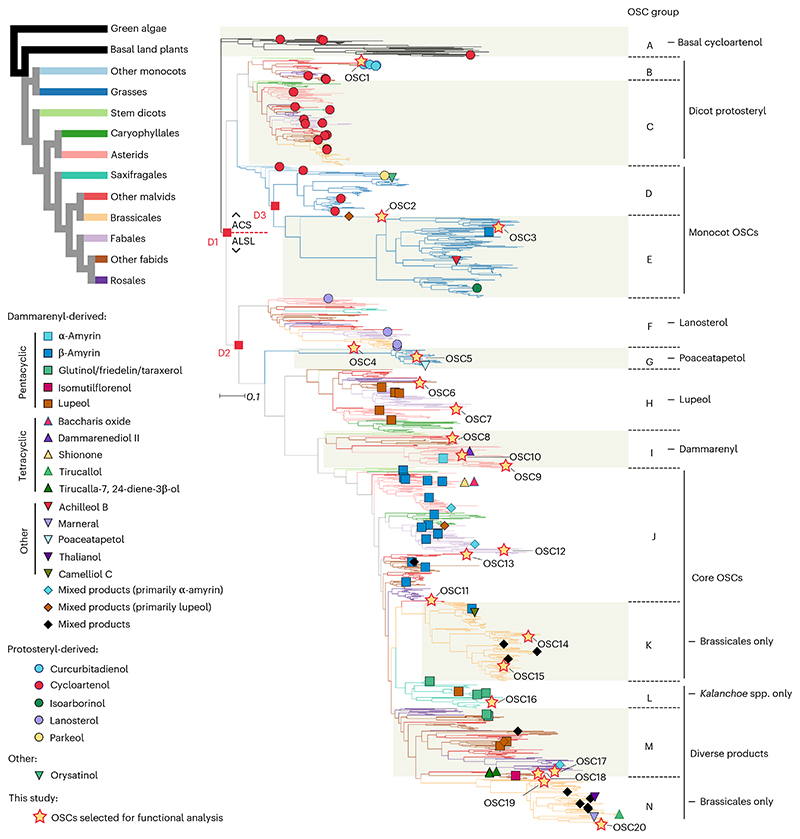
Mining OSC sequence diversity across the Viridiplantae. Maximum-likelihood tree of 1,405 OSC sequences mined from plant genomes, along with 83 previously characterized OSCs (Supplementary Data 2). Symbols on the tree indicate the products made by these characterized OSCs (key, bottom left). The structures of compounds named here are presented in Supplementary Fig. 2. Top left: the branch colors indicate the plant clades to which the OSCs belong. The OSCs are grouped into clades A–N, following nomenclature published previously^5,30,31^, with some OSC groups sharing apparent functional and/or plant clade specificity. D1 represents the ancient gene duplication of the ACS and ALSL OSCs as in ref. 31. D2 and D3 represent the origins of dicot (D2) and monocot (D3) OSCs that make dammarenyl-derived products. The OSCs selected for functional analysis here (OSCs 1–20) are indicated by yellows stars.

A maximum-likelihood tree of the amino acid sequences of the 1,405 high-quality OSC genes, along with 83 functionally characterized OSCs (Supplementary Data 2), is shown in Fig. 2. The structures of the products of these enzymes are shown in Supplementary Fig. 2. The OSC clades are labeled A–N following the nomenclature used in previously published analyses^5,30,31^, with cycloartenol synthases from green algae and early diverging land plants forming the root (group A). Eudicot OSCs that produce protosteryl-derived products form groups B and C; group B contains both cycloartenol and cucurbitadienol synthases, while group C appears to contain cycloartenol synthases only. Monocot OSCs that make protosteryl-derived and dammarenyl-derived products fall primarily into groups D and E, respectively. Group F consists of lanosterol synthases, which are specific to eudicots^32,33^. Group G is a monocot-specific clade from which a single OSC producing the bicyclic product poaceatapetol has been characterized in rice^34^. All characterized OSCs in group H are lupeol synthases, although lupeol synthase activity is also found in other groups. The remaining groups (I–N) represent a large monophyletic dicot clade that contains β-amyrin synthases and other diverse types of plant OSCs^5,30,31^. Groups K and N are specific to brassicas, corresponding respectively to the clades I and II reported by Liu et al^35^. Group J is of particular note, as all eudicot genomes (with the exception of *Kalanchoe* spp. and *Brassica* spp.) have at least one OSC in this group. Groups J and K contain characterized β-amyrin synthases (primarily in group J) and also multifunctional OSCs that make α-amyrin and other mixed products. As such, these two groups appear to form a (paraphyletic) core group of OSCs that likely function as β-amyrin synthases or as other types of dammarenyl-derived triterpene synthases. Outside of these core groups, groups I, L, M and N all contain OSCs that produce dammarenyl-derived compounds, although there is little indication of conservation of function within the groups. Duplication 1 (D1) (Fig. 2) represents the ancient gene duplication of the ancestral cycloartenol synthase (ACS) and the ancestral lanosterol synthase-like (ALSL) OSCs (>140 million years ago)^31^. D2 and D3 represent the origins of dicot (D2) and monocot (D3) OSCs that make dammarenyl-derived products. It is evident that the monocots have independently evolved dammarenyl-derived biosynthetic function through the ACS clade, as opposed to the ALSL clade as in eudicots. A summary of the representation of the different OSC groups across the Viridiplantae is provided in Extended Data Fig. 2.

**Fig. 3:**
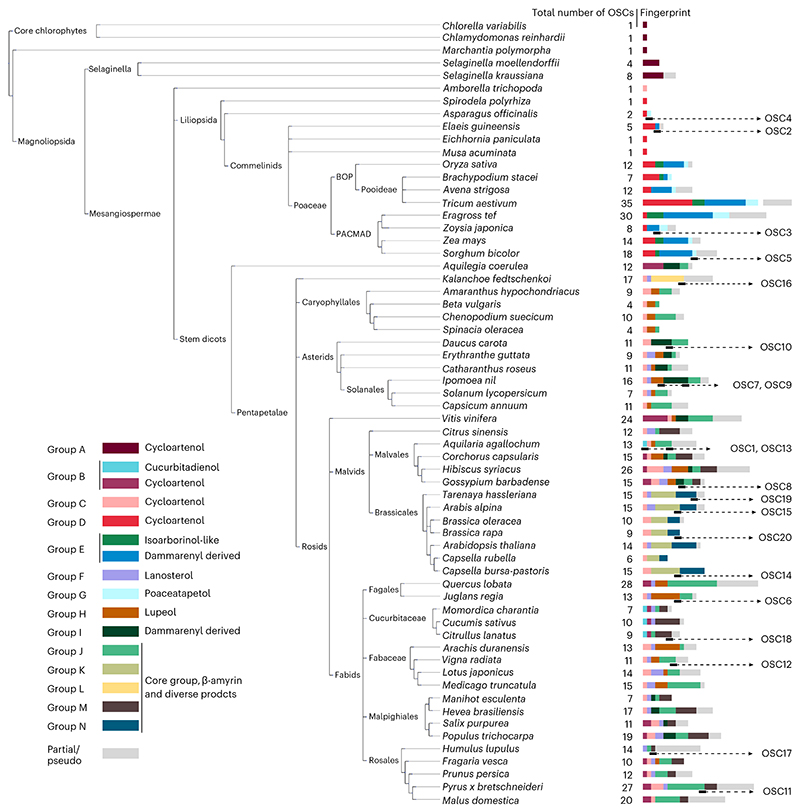
OSC profiling across diverse plant species. Total number of OSCs mined from representative genome data and their putative OSC groups as defined by pHMM homology is shown. The OSCs selected for characterization here (OSCs 1–20; Supplementary Table 1 and Supplementary Data 2) are indicated.

Current Pfam classification groups all eukaryotic OSCs into a single family together with prokaryotic squalene-hopene cyclases (SQHop_cyclase_N/C: PF13249, PF13243). Having established the above model of phylogenetic diversity of OSCs across plants, we next generated profile hidden Markov models (pHMMs) (Supplementary Data 3) for each of OSC groups A–N to enable classification of OSCs without the need for alignments and tree building (an approach that we term OSC profiling). To demonstrate the utility of our pHMM-based profiling approach, we applied this to the genomes listed in Supplementary Data 1 (Supplementary Fig. 3). Figure 3 shows a subset of these OSC profiles for species across a range of plant clades, while profiles for selected plant clades can be found in Supplementary Fig. 4. This analysis again demonstrates how the diversity of OSCs differs across plant lineages and allows rapid assessment of potentially interesting or unusual OSCs in a given species. It is also possible to quickly assess clade-specific or species-specific blooms of OSCs of different groups (as defined by a mean copy number of ≥4 (Supplementary Fig 5), for example, group A OSCs in spikemosses (*Selaginella* spp.), group L OSCs in widow’s thrill stonecrops (*Kalanchoe* spp.) and the expansion of the apparently lupeol-specific group H OSCs in the Fabales species walnut (*Juglans regia*) and the peanut relative *Arachis duranensis*. Some caveats apply to the interpretation of the outputs of this approach. For example, high overall OSC numbers can in some cases be because of polyploidy (for example, bread wheat, *Triticum aestivum*), while relatively low numbers may be indicative of poorer quality genome assemblies (for example, for hop, *Humulus lupulus*, and bottle gourd, *Lagenaria siceraria*) (Supplementary Fig. 3). OSC groups can also be observed to have variable propensity to occur in putative biosynthetic gene clusters (BGCs; as detected by plantiSMASH)^36^, such as the notable clustering seen with group N OSCs (that is, those identified as clade II OSCs by Liu et al.^35^) (Supplementary Fig. 6). There is enrichment of the apparently conserved OSC group F (lanosterol) and group H (lupeol) in BGCs. No BGCs have been characterized thus far that use OSCs from these groups, suggesting that undiscovered metabolic pathways might be found in plants using OSCs from these groups.

### Functional analysis of selected OSC sequences

**Fig. 4:**
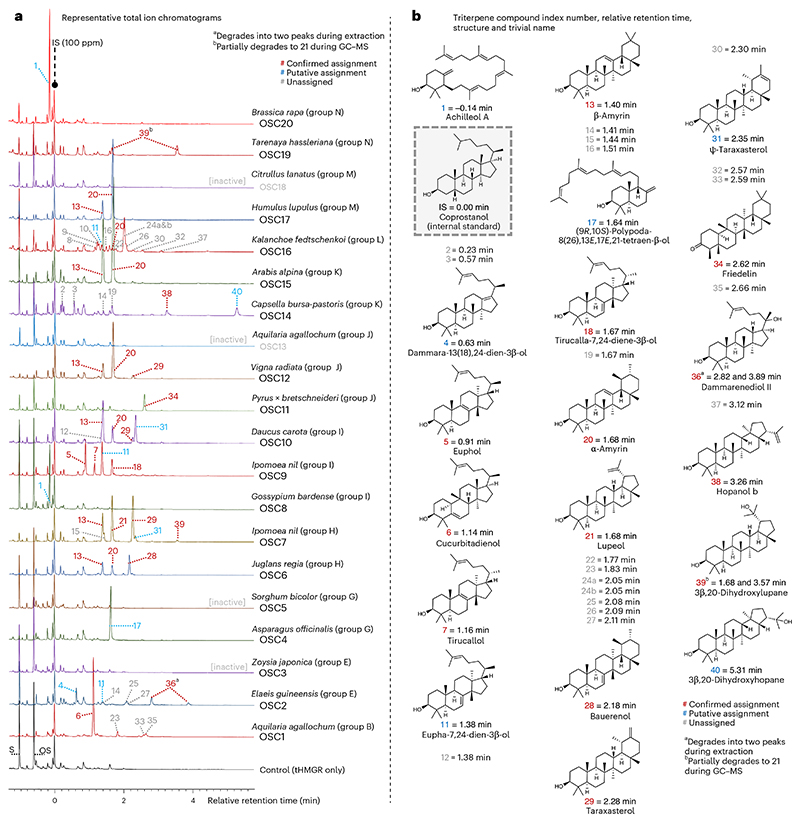
Products of OSCs 1–20. **a**, Representative TICs for OSCs 1–20 and the control (tHMGR expressed alone). Retention times are relative to an IS (coprostanol, 100 ppm). The product peaks are numbered 1–40 in order of increasing retention time (EI spectrum in Supplementary Data 4). S, squalene; OS, oxidosqualene. **b**, Summary of key data (relative retention times and structures where known) for the products shown in **a** (in numerical order from top left, reading down the columns). Red numbers, assignments confirmed by comparison with authentic standards or NMR. Blue numbers, putative assignments based on comparison of EI spectrum to those reported in the literature (details in Supplementary Figs. 8–28). Gray numbers, unassigned products along with their respective relative retention times. Compound **36** (†) degrades into two peaks during extraction (Supplementary Fig. 12); compound **39** (**‡**) partially degrades to compound **21** during GC–MS (Supplementary Fig. 8). *The relative retention time scale is approximate. Source TICs are provided in Supplementary Data 4.

We next selected a total of 20 previously uncharacterized OSC sequences for functional analysis (labeled OSCs 1–20 in Figs. 2 and 3; alignment shown in Supplementary Fig. 7). These OSCs were prioritized because they are either located in divergent branches of the phylogenetic tree or in clades that have not been well characterized. Further information about these 20 OSCs, along with the reason for their selection, is provided in Supplementary Table 1. OSC coding sequences were synthesized and cloned into the pEAQ-HT-DEST1 expression vector before *Agrobacterium-*mediated transient expression in *Nicotiana benthamiana*^37^. The OSCs were coexpressed with a feedback insensitive mutant variant of HMG-CoA reductase (tHMGR) that boosts precursor supply and, hence, triterpene yields^38,39^. Then, 5 days after agroinfiltration, the leaves were isolated and extracts were analyzed by gas chromatography–mass spectrometry (GC–MS). The total ion chromatograms (TICs) of extracts from leaves expressing each OSC were compared to those of leaves infiltrated with *A. tumefaciens* containing the tHMGR expression construct alone (which served as a negative control) (Fig. 4a). The triterpenes present in these extracts were monitored on the basis of retention time and estimated abundance relative to an internal standard (IS; coprostanol, 100 ppm). Unique OSC products were subsequently indexed by their electron impact (EI) mass spectra and numbered in order of increasing relative retention time (Supplementary Data 4). Putative structural assignments of the indexed triterpenes were initially made on the basis of a comparison of the fragmentation patterns observed in each EI spectrum to those reported in the literature for known triterpene compounds. These putative assignments were then confirmed by comparison to authentic standards where available. Remaining unconfirmed assignments were selectivity targeted for further investigation and either confirmed or revised on the basis of nuclear magnetic resonance (NMR) analysis (Fig. 4b and Supplementary Figs. 8–28).

**Extended Data Fig. 3:**
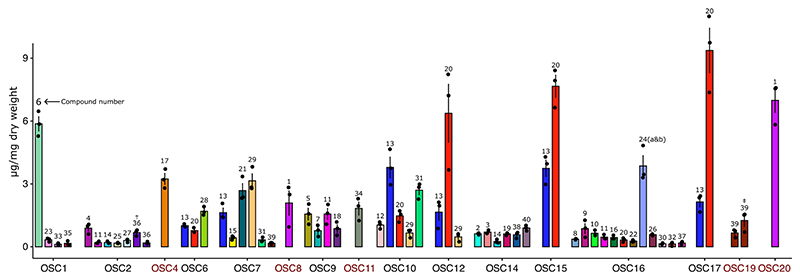
Estimated accumulation of products generated by OSCs. Levels were estimated by GC-MS relative to an internal standard (coprostanol 100 ppm) following transient expression in *N. benthamiana*. Error bars represent standard error of the mean (three biological replicates). **NB:** Compound **36** (†) degrades into two peaks during extraction; compound **39** (**‡)** partially degrades to compound **21** during GC-MS (See, Fig. 4a and Supplementary Figs. 8 and 12). Names of OSCs that make multiple products (multifunctional OSCs) are in black, while those of OSCs that make one major product (that is are committed) are in red. See Supplementary Data 4 – Supplementary Database for source Total Ion Chromatograms and Source Data for this figure.

**Extended Data Fig. 4:**
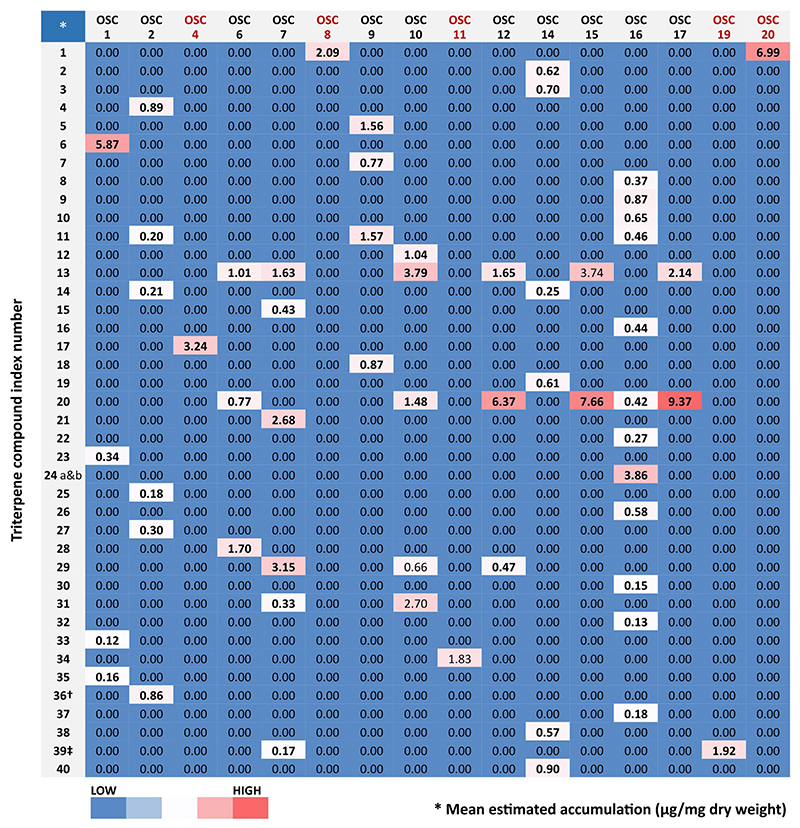
Summary matrix showing the overall mean estimated accumulation of triterpene compounds 1–40 (µg/mg dry weight) for each OSC tested. For compounds 36 and 39, the sum of their artefactual degradation peaks are used. Compound **36** (†) degrades into two peaks during extraction; compound **39** (**‡)** partially degrades to compound **21** during GC-MS (See Fig. 4a and Supplementary Figs. 8 and 12). See Supplementary Data 4 – Supplementary Database for source Total Ion Chromatograms.

Triterpene products were detected by GC–MS for 16 of the 20 OSCs tested (Fig. 4a). Overall, a total of 41 distinct triterpene products were detected. These included 19 known or putatively assigned triterpenes and a further 22 unassigned triterpenes (Fig. 4b and Supplementary Table 1). The product profiles of the 16 functional OSCs are shown in Extended Data Figs. 3 and 4. Five of the OSCs produced one major detectable product, while the remaining 11 produced multiple products. For the four OSCs that did not produce any detectable products, this may be because of errors in the publicly available sequences, failure to express or fold correctly, lack of catalytic activity or conversion of products to undetected compounds in *N. benthamiana*.

### OSC16 produces a unique E-ring methyl group arrangement

**Fig. 5:**
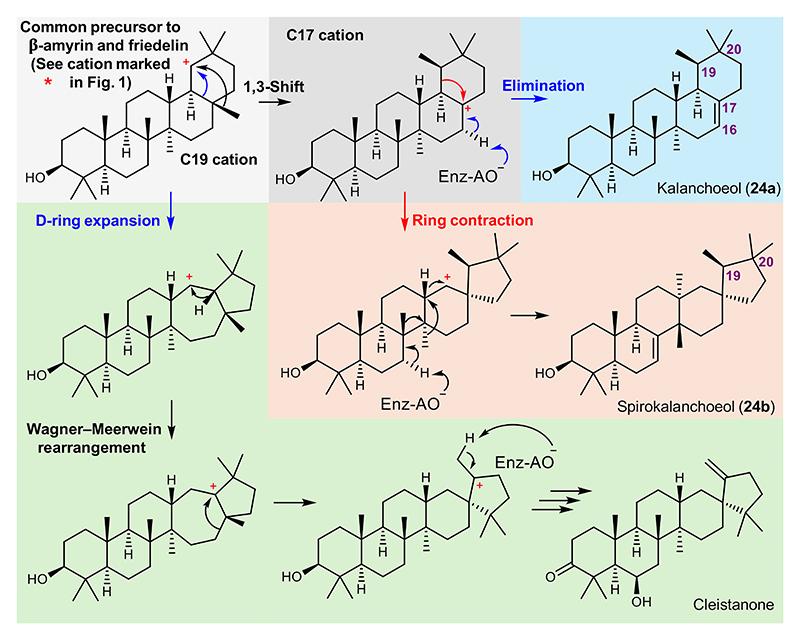
Proposed reaction pathway for products of OSC16 and cleistanone-type triterpenoids. The structures of kalanchoeol (**24a**) and spirokalanchoeol (**24b**) produced by OSC16 and their proposed reaction pathway relationship to a common cationic intermediate shared with pentacyclic triterpenes such as β-amyrin (**13**) and friedelin (cation marked with the red asterisk in Fig. 1). The difference between the likely reaction mechanisms of these scaffolds and the known spriocyclized cleistanone-type triterpenoids is shown. The structure of kalanchoeol (**24a**) can be seen to support the proposed 1,3-shift of the C28 methyl group before ring contraction in the formation of spirokalanchoeol (**24b**). The stereochemistry of kalanchoeol (**24a**) and spirokalanchoeol (**24b**) is presented in its most likely configuration based on the stereospecific rearrangements required from intermediatory dammarenyl-derived cations and may be open to future revision.

The group L enzyme OSC16 from *Kalanchoe fedtschenkoi* (Fig. 2) was found to be a highly multifunctional enzyme producing a total of 13 compounds. Most of these, however, were minor side products (including dammarenyl-derived triterpenes eupha-7,24-dien-3β-ol (**11**) and α-amyrin (**20**)) and the product profile was dominated by two coeluting unassigned triterpenes (compounds **24a** and **24b**) (Fig. 4, Extended Data Figs. 3 and 4 and Supplementary Table 1). These two compounds were subsequently resolved and isolated by preparative high-performance liquid chromatography (HPLC) following infiltration of 30 *N. benthamiana* plants. Extensive one-dimensional and two-dimensional NMR analysis of the resulting fractions (Supplementary Figs. 29–36) revealed their structures to have the connectivity of pentacyclic triterpene scaffolds, proposed herein, and named kalanchoeol (**24a**) and spirokalan-choeol (**24b**) (Fig. 5). Spirokalanchoeol (**24b**) has a 6,6,6,6,5 scaffold containing an unusual spirocyclized D/E-ring junction. Although this basic ring architecture is present in some previously reported triterpenoid natural products such as cleistanone (produced by the plant species *Cleistanthus indochinensis*)^40^, the methyl group arrangement in the E ring of spirokalanchoeol (**24b**) is not. The presence of the methyl group at C19, which is vicinal to an intact pair of geminal methyl groups at C20, would appear to require a rarely invoked 1,3-alkyl shift before the ring contraction that we propose affords the spirocyclized D/E-ring junction (Fig. 5). The structure of the product kalanchoeol (**24a**) supports this proposed mechanism; it represents an alternative resolution of the C17 cation that would be generated from this proposed 1,3-alkyl shift, with C16 proton elimination instead of the ring contraction affording the D-ring alkene of kalanchoeol (**24a**). In contrast, the triterpene precursor of the cleistanone-type triterpenoids is proposed to form through D-ring expansion followed by a Wagner–Meerwein rearrangement (Fig. 5)^40^. The stereochemistry of kalanchoeol (**24a**) and spirokalanchoeol (**24b**) is presented in its most likely configuration based on the stereospecific rearrangements required from intermediatory dammarenyl-derived cations. The group L clade to which OSC16 belongs is unique to *Kalanchoe*. The C19 cation that precedes the 1,3-alkyl shift in the formation of kalanchoeol (**24a**) and spirokalanchoeol (**24b**) is shared with the reaction pathways of other previously characterized group L OSCs (Fig. 2) that produce known pentacyclic 6,6,6,6,6 triterpenes such as taraxerol, friedelin and glutinol (Figs. 1 and 5). A detailed phylogenetic tree showing the group L OSCs along with the triterpene products of OSC16 and other characterized group L enzymes is shown in Supplementary Fig. 37. Detailed computational and experimental investigations of the mechanism of action of OSC16 will form the basis of future studies.

### Broader insights into OSC function and diversification

**Extended Data Fig. 5:**
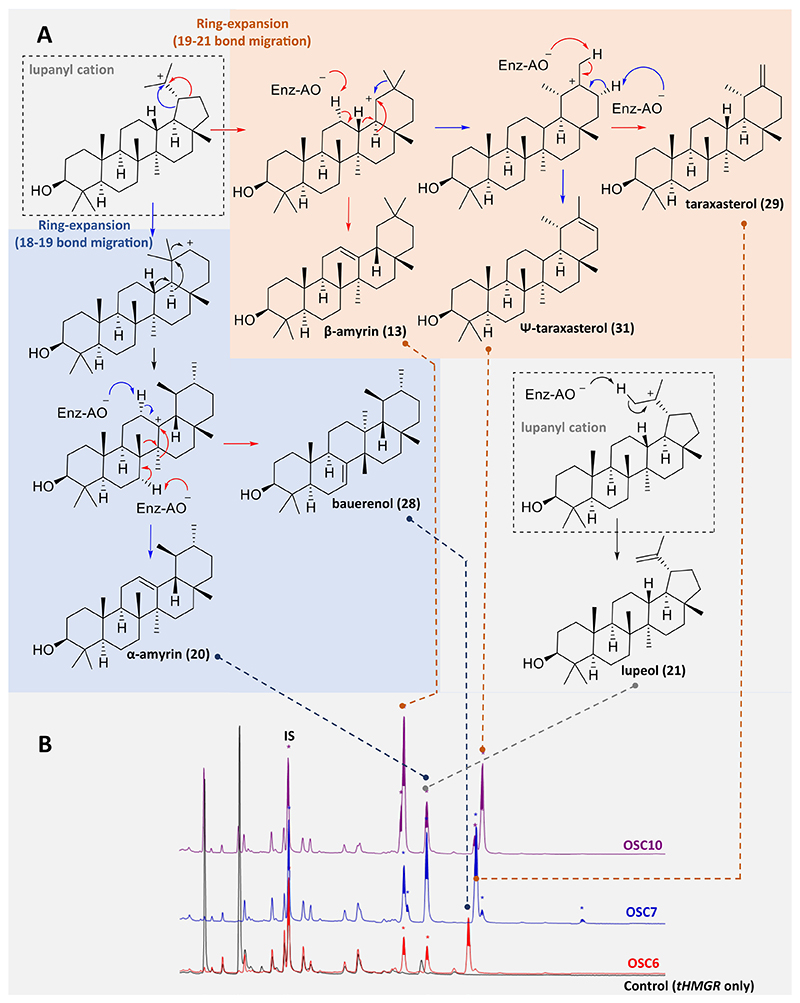
Products of OSCs 6, 7 and 10. **a**, The reaction pathway relationship between the triterpene products of OSC6, OSC7 and OSC10, and their shared intermediatory cation (the lupanyl cation). **b**, Representative Total Ion Chromatograms of extracts from *N. benthamiana* leaves expressing OSC6 (red), OSC7 (blue), and OSC10 (purple); black, control (*tHMGR* only). IS, internal standard (coprostanol, 100 ppm).

The products of all 16 OSCs characterized in this study are summarized in Supplementary Table 1, along with their species of origin and the specific names that we have assigned them. Our analyses revealed a wealth of additional insights into OSC function and diversification, of which several are highlighted here. For example, OSC6 and OSC7 both belong to group H, a phylogenetic group previously believed to be a functionally conserved group of committed lupeol synthases (Fig. 2). OSC10 belongs to group I, which contains a previously characterized near-committed α-amyrin synthase (Fig. 2). These three enzymes were all found to be mixed-amyrin synthases, producing β-amyrin (**13**) along with several other closely related products that all share the lupanyl cation (the terminal cation in the formation of lupeol) as a common intermediate in their respective reaction pathways (Extended Data Fig. 5). Thus, as for OSC16, the structural diversity seen in the product profiles of these OSCs represents an expansion of previously known cladal functionality.

OSC8 and OSC20 both produce the same monocyclic product, putatively assigned as achilleol A (**1**) (Fig. 4), and are rare examples of committed OSCs that produce a monocyclic product, the only other example being the near-committed *Arabidopsis thaliana* OSC CAM1 (ref. 41), which produces the monocyclic triterpene, camelliol C (Fig. 2). OSC8 and OSC20 are distantly related (group I and group N, from *Gossypium bardense* and *Brassica rapa*, respectively) demonstrating apparent independent evolution. OSC19 represents a first-in-class enzyme in the production of a committed 3β,20-dihydroxylupane synthase (**39**). This diol has only previously been reported as a minor side product of a highly multifunctional group K OSC (LUP1) from *A. thaliana*^42^. OSC19 is a group N OSC from *Tarenaya hassleriana* (a close sister species of *A. thaliana*). OSC11 (a group J OSC from *Pyrus* × *bretschneideri*) is a committed friedelin synthase. OSC11 may, therefore, be an example of independent evolution of friedelin synthase functionality compared to the previously characterized committed or near-committed friedelin synthases from *Kalanchoe daigremontiana* (group L)^43^, *Maytenus ilicifolia* (group M)^44^ and *Tripterygium wilfordii* (group M)^45^.

OSC1 from agarwood (*Aquillaria agallochum*, syn. *A. malaccensis*) produced >90% cucurbitadienol (**6**), effectively representing a committed enzyme (Extended Data Figs. 3 and 4)—a cucurbitadienol from outside the Cucurbitales. Known cucurbitadienol synthases form a conserved subset of group B OSCs that are closely related to eudicot cycloartenol synthases (Fig. 2). OSC1 is also located within this clade and, thus, may share a common evolutionary origin with the other previously characterized cucurbitadienol synthases. This suggests that the functional conservation of this clade likely extends to a broader range of species and inclusion may be highly predictive of cucurbitadienol synthase activity.

**Extended Data Fig. 6:**
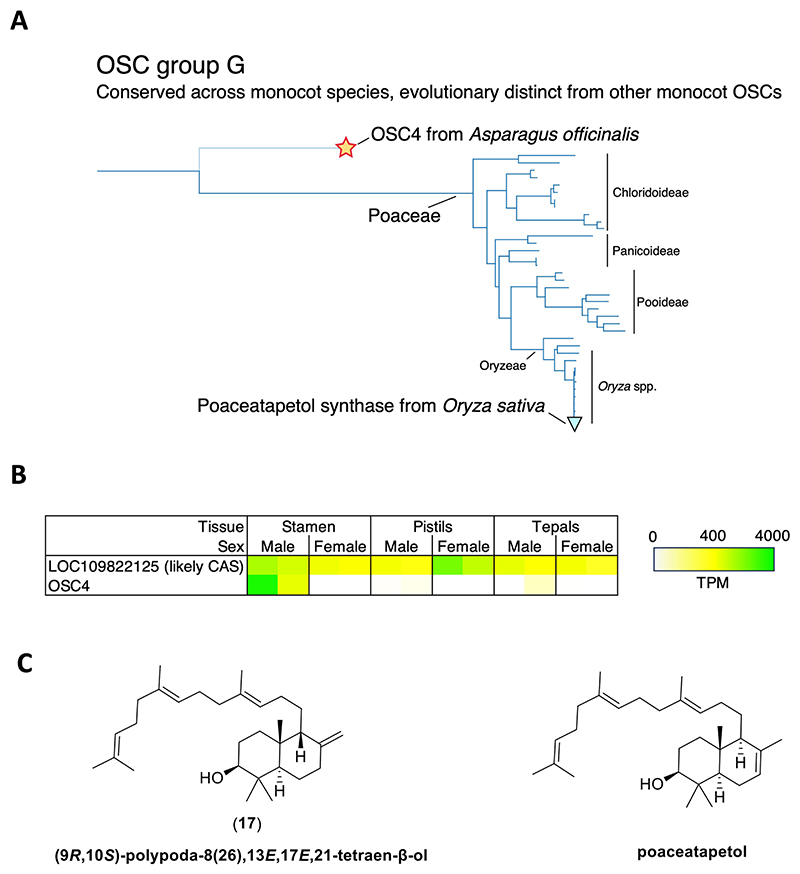
Production of a bicyclic poaceatapetol-like triterpene from *Asparagus officinalis*. **a**, OSC group G from Fig. 2., showing plant taxonomic clades where this OSC group was found, OSC4 from *A. officinalis* and the characterized poaceatapetol synthase from *O. sativa*. OSC4 produces a bicyclic poaceatapetol-like triterpene, (9R,10S)-polypoda-8(26),13E,17E,21-tetraen-β-ol (**17**). **b**, Heatmap of expression data from NCBI BioProject PRJNA435625 (Salmon TPM counts normalized with DESeq2)^54^ of the two OSC sequences found in the *A. officinalis* genome: OSC4 and a likely cycloartenol synthase. OSC4 has specific expression in the male stamens (unlike the expected, broad expression pattern of the likely CAS). This suggests a conserved function for group G OSCs as pollen specific compounds across wider monocot species than only the Poaceae. **c**, Structures of polypoda-8(26),13E,17E,21-tetraen-β-ol (**17**) and poaceatapetol.

The product of OSC4 (from the monocot *Asparagus officinalis*) was putatively assigned as the bicyclic triterpene (9*R*,10*S*)-polypoda-8(26),13*E*,17*E*,21-tetraen-β-ol (**17**), a closely related isomer of poaceata-petol (Extended Data Fig. 6), and is a committed OSC outside of the Poaceae that produces a bicyclic poaceatapetol-like triterpene. Previously reported poaceatapetol synthases from grass species are pollen specific. The gene encoding OSC4 is specifically expressed in the stamen of *A. officinalis*, suggesting a conserved biological role of poaceatapetol-like synthases within the monocots, extending beyond the Poaceae (Extended Data Fig. 6).

### Mutation of OSC9 reveals residues critical for product specificity

**Extended Data Fig. 7:**
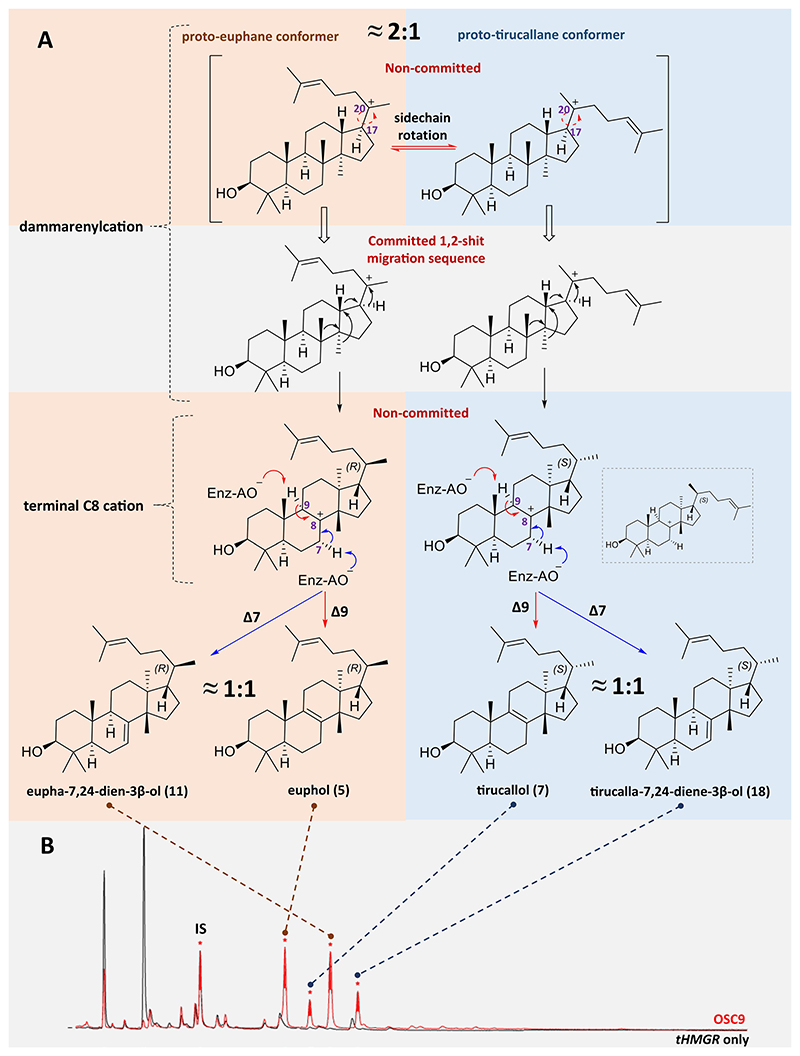
Products of OSC9. **a**, The reaction pathway relationship between the dammarenyl cation and the product profile of OSC9, highlighting the committed nature of the 1,2-shift migration sequence with variation arising only at the initiation and termination of this re-arrangement (differential sidechain rotation and position of final proton elimination). **b**, Representative Total Ion Chromatograms for leaf extract from *N. benthamiana* expressing OSC9 (red). Black, control (*tHMGR* only). IS, internal standard (coprostanol, 100 ppm).

**Fig. 6:**
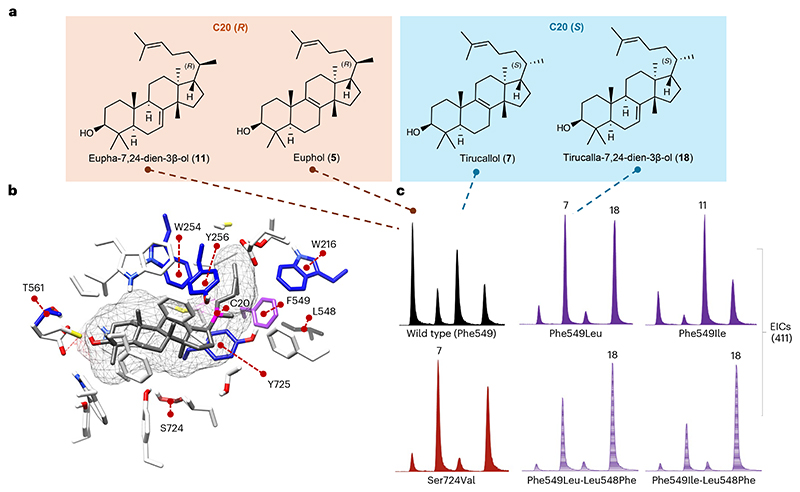
OSC9 product ratios change with substitution of key residues. **a**, Structures of eupha-7,24-dien-3β-ol (**11**), euphol (**5**), tirucallol (**7**) and tirucalla-7,24-diene-3β-ol (**18**). **b**, Lowest-energy (affinity = −16.5 kcal mol^−1^) flexible-residue docking model of the dammarenyl cation with the OSC9 homolog model (Supplementary Data 5, PDB file of the OSC9 homology model; Supplementary Data 6, PDBQT file of the lowest-energy binding model of the dammarenyl cation and OSC9 flexible residues). The position of the C20 cationic carbon and the residues targeted for substitution in this study are highlighted. **c**, Representative extracted ion chromatograms (EICs, selected ion 411) for the wild-type OSC9 enzyme and mutant variants (scaled to the largest peak) for key substitutions.

OSC9 from Japanese morning glory (*Ipomoea nil*) produces four tetracyclic triterpenes: euphol (**5**), eupha-7,24-dien-3β-ol (**11**), tirucallol (**7**) and tirucalla-7,24-diene-3β-ol (**18**) (Figs. 4 and 6a and Extended Data Figs. 3 and 4), with euphanes (**5** and **11**) being favored in an approximately 2:1 ratio over the tirucallanes (**7** and **18**) and the respective Δ9 (**5** and **7**) and Δ7 (**11** and **18**) alkene isomers of each class being produced in a roughly 1:1 ratio. It has traditionally been thought that the euphane and tirucallane scaffolds are derived from 1,2-shift migrations of the dammarenyl cation, with the differing stereochemical configuration at C20 resulting from prior differential side-chain rotation around the C17–C20 bond (Extended Data Fig. 7)^12^. The discovery that OSC9 produces both euphanes and tirucallanes supports the derivation of these scaffolds from the same intermediatory tetracyclic cation. The respective alkene isomers represent resolution of the same terminal C8 cation through proton elimination at either C7 or C9 (Extended Data Fig. 7). Therefore, OSC9 shows a committed sequence of 1,2-shift migrations, with different products being derived only from the initiation and termination of this cascade. Given the refined control of scaffold rearrangement observed, it is tempting to speculate that OSC9 represents an OSC captured in the process of evolving through the duplication and diversification of a committed euphane synthase ancestor to an enzyme that can produce the tirucallane scaffold (or vice versa).

**Extended Data Fig. 8:**
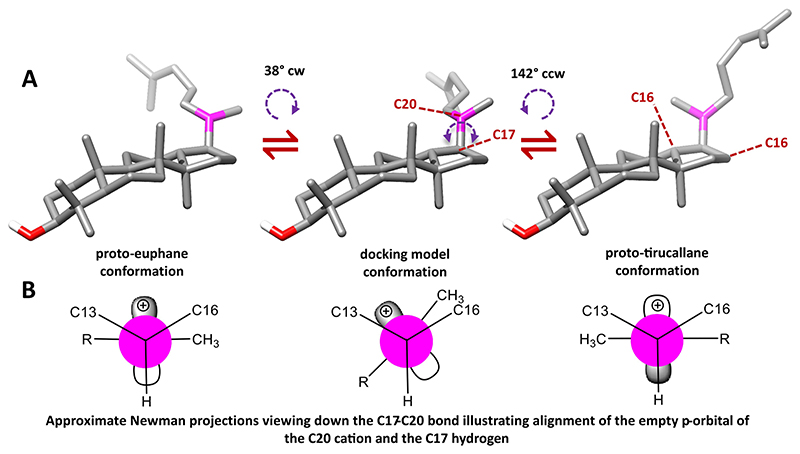
Proto-euphane and proto-tirucallane confirmations. **a**, The docking model conformation of the dammarenyl cation and the approximate sidechain rotation required around the C17-20 bond to access the proto-euphane or proto-tirucallane conformation allowing the first 1,2-hydride shift (cw = clockwise, ccw = counterclockwise). **b**, Approximate Newman projections viewing down the C17-C20 bond, for the conformations above in panel A, illustrating alignment of the empty p-orbital of the C20 cation and the C17 hydrogen (R=sidechain). C20 is colour coded between panels (light purple).

**Extended Data Fig. 9:**
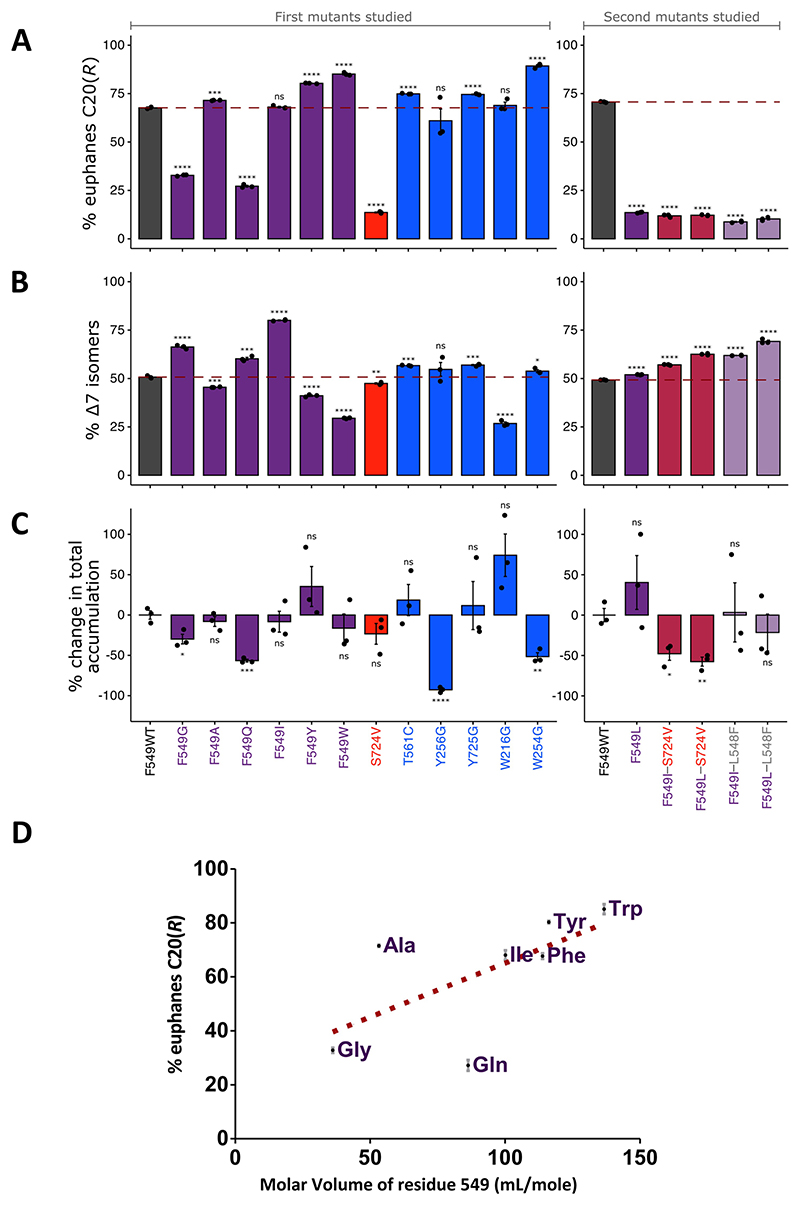
Euphane and tirucallane production resulting from mutations of OSC9. **a**, Mean euphane content as a percentage of total euphane and tirucallane content for each OSC product profile. **b**, Mean Δ7 isomer content as a percentage of total Δ9 and Δ7 isomer content for each OSC product profile. **c**, Percent change in total accumulation of euphanes and tirucallanes compared to the mean wildtype value (μg/mg). **d**, Linear regression of % euphane content and the molar volume (mL/mole) of different amino acids at residue 549 (Gly = 36.1, Ala = 53.2, Gln = 86.3, Ile= 100.1, Phe = 113.9, Tyr = 116.2, Trp = 136.7, Values from Zamyatnin, A. A.^44^ Error bars for all are standard error of the mean (three biological replicates). **** = p ≤ 0.0001, *** = p ≤ 0.001, ** = p ≤ 0.01, * = p ≤ 0.05, ns = p > 0.05) - independent two-tailed T-Tests. Full source data with p values available in Source Data for this figure. See Supplementary Data 4 – Supplementary Database for source Extracted Ion Chromatograms.

Because of the mechanistic importance of this product profile, we next investigated the control of euphane versus tirucallane scaffold production through docking-directed mutagenesis studies of OSC9. The positioning of the dammarenyl cation in the active site of OSC9 was explored by in silico flexible-residue docking to a homology model of the enzyme (Supplementary Data 5). Inspection of the lowest-energy binding model (Fig. 6b and Supplementary Data 6) revealed that the dammarenyl cation adopted a conformation closest to that required for the first hydride shift to result in the production of euphanes, requiring just 38° of clockwise side-chain rotation compared to 142° of counterclockwise rotation for the production of tirucallanes (Extended Data Fig. 8). This is consistent with a conformational equilibrium that favors euphanes. The amino acid sequence of OSC9 was next aligned with two known committed tirucallane synthases (Supplementary Fig. 38), tirucallol synthase (CrOSC) from *Capsella rubella*^35^ and tirucalla-7,24-diene-3β-ol synthase (AiOSC1) from *Azadirachta indica*^46^. Active-site amino acid residues were then selected for investigation on the basis of their position in the docking model and/or conserved sequence divergence from the tirucallane synthases (Fig. 6b and Supplementary Fig. 38). Residues F549, S724, T561, Y256, Y725, W216 and W254 were prioritized for mutational analysis. The rationale for this was as follows. Inspection of the docking model suggested that F549 occupied an area of the active site where steric effects could influence side-chain rotation and it was found to be substituted for residues of smaller molar volume in both the tirucallane synthases (leucine or isoleucine)^47^. As such, a series of OSC9 mutants differing in the molar volume of residue 549 were designed for investigation (F549G, F549A, F549Q, F549I, F549Y and F549W). Conserved active-site substitutes were observed at S724 and T561, being valine and cysteine, respectively, in both tirucallane synthases (S724V and T561C). Two tryptophan residues situated in the side-chain binding pocket were also individually substituted for glycine (W216G and W254G). All were selected to investigate any potential effect on euphane versus tirucallane production. The docking analysis also showed that the cationic carbon (C20) was sandwiched between two potentially stabilizing aromatic tyrosine residues that were conserved across all three enzymes. These were individually substituted for glycine to investigate their potential importance in favoring tetracyclic compounds (Y256G and Y725G). This initial series of 12 OSC9 mutants (F549G, F549A, F549Q, F549I, F549Y, F549W, S724V, T561C, Y256G, Y725G, W216G and W254G) was subjected to functional analysis. Mean euphane content as a percentage of total euphane and tirucallane content, mean Δ7 isomer content as a percentage of total Δ9 and Δ7 isomer content and the percentage change in total accumulation of euphanes and tirucallanes for each mutant compared to the wild type are shown in Extended Data Fig. 9.

The product profiles of mutants F549G, F549A, F549Q, F549I, F549Y and F549W revealed a potential positive relationship between the molar volume of residue 549 and percentage euphane content (Extended Data Fig. 9a,d). However, the smaller molar volume of isoleucine at this position (seen in the tirucallol synthase) was not sufficient to favor tirucallanes in OSC9. The greatest effect on percentage euphane content was observed with mutant S724V (Fig. 6c). This mutant clearly favored production of tirucallanes, with the percentage euphane content dropping from 67.7% (95% confidence interval (CI): 66.6% to 68.8%) to 13.6% (95% CI: 12.6% to 14.7%) (Extended Data Fig. 9a). This was a conserved substitution seen in both tirucallane synthases (Supplementary Fig. 38). However it is not clear why S724V favors tirucallanes from a three-dimensional (3D) structural perspective because this residue is not situated in the side-chain binding pocket and instead sits over the B/C-ring junction of the dammarenyl cation (Fig. 6b).

These initial results prompted the investigation of additional mutants. Broader inspection of the sequence alignment, allowing consideration of neighboring residues that did not directly occupy the active site in the docking model, revealed that residue 548 of OSC9 is leucine, whereas it is phenylalanine in both tirucallane synthases. Thus, the conserved difference between OSC9 and both tirucallane synthases may be better described as a switch in positioning of residue 548 and 549, rather than simply a single substitution at the latter (Supplementary Fig. 38). L548 sits behind the active-site-orientated face of F549 in the docking model (Fig. 6b). To investigate whether switching residues 548 and 549 had a different effect to single substitutions at residue 549, both tirucallol and tirucalla-7,24-diene-3β-ol synthase donor switch mutants were tested (F549I;L548F and F549L;L548F), along with the tirucalla-7,24-diene-3β-ol synthase donor single 549 substitution (F549L) that was not tested in the first set of mutants studied. Both switch mutants clearly favored production of tirucallanes, with the percentage euphane content dropping to 8.8% (95% CI: 7.6% to 9.9%), and 10.3% (95% CI: 8.2% to 12.3%) for F549I;L548F and F549L;L548F, respectively, compared to 70.7% (95% CI: 69.9% to 71.4%) for the wild-type enzyme (Extended Data Fig. 9a and Fig. 6c). The F549L mutant also displayed a similar drop in percentage euphane content to 13.6% (95% CI: 12.9% to 14.3%), which was not seen with the F549I single substitution. However, in the first round of substitutions, mutant F549I showed an increase in production of Δ7 isomers (Extended Data Fig. 9b and Fig. 6c). Thus, double mutants F549L;S724V, and F549I;S724V were also tested to probe a potential role of leucine versus isoleucine in the control of Δ7 versus Δ9 isomers. These both showed the expected favoring of tirucallanes attributed to the S724V substitution. However no differential effect in the production of Δ7 isomers was observed, with both F549L and F549I substitutions resulting in a small increase in Δ7 isomer production compared to the wild type (Extended Data Fig. 9). Taken together these results indicate that there are multiple substitutions that can independently lead to the favoring of tirucallane products. Both valine in place of serine at position 724 and a switch in positioning of phenylalanine with either leucine or isoleucine at positions 549 and 548 may be diagnostic of tirucallane synthases (Fig. 6c).

## Discussion

**Extended Data Fig. 10:**
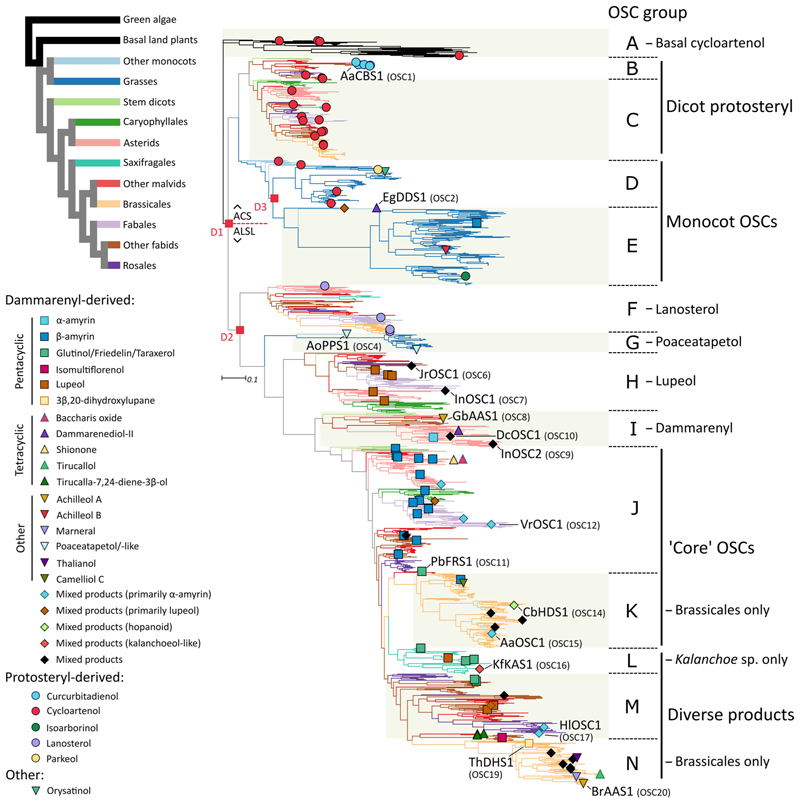
Phylogenetic tree shown in Fig. 2 with the functions of the newly characterised OSCs from the study included. See Fig. 2 and Supplementary Table 1.

The development of a scalable and robust high-throughput genome mining pipeline has made available direct access to the coding sequences of thousands of plant OSC genes, thereby providing a framework for functional characterization. Many of these genes were previously hidden by the lack of publicly available genome annotations. Large-scale phylogenetic analysis, incorporating previously functionally characterized OSCs, affords the opportunity for the targeted selection of enzymes for study. This potential was demonstrated through the functional screening carried out in this work. Functional analysis of just 20 OSCs (selected because they were either located in divergent branches of the phylogenetic tree or in clades that have not been well characterized) resulted in the discovery of triterpene scaffolds, product profiles within clades previously believed to be functional conserved, and committed OSCs that produce previously reported triterpenes for which the producing enzyme was not known or exemplars were rare (Supplementary Table 1). It revealed examples of the independent evolution of identical product profiles. Critically, it also led to the discovery of OSCs with product profiles that yield mechanistic insights into the control of specific reaction pathways. An updated version of the phylogenetic tree shown in Fig. 2 annotated with functional enzymatic information for the 16 functional OSCs characterized in this study can be found in Extended Data Fig. 10. Of the 170 previously characterized OSCs, 50 (that is, 29%) were multifunctional (14). In our study, we found that 12 of 16 OSCs (that is, 75%) were multifunctional. It is conceivable that the proportion of multifunctional OSCs within our study may have been skewed by the selection of divergent OSCs, although our sample size is too small to robustly make this conclusion. Analysis of a subset of previously characterized OSCs using our workflow gave results consistent with the literature, confirming that any discrepancy in the proportion of dedicated/multifunctional OSCs is unlikely to be because of the methods used (Supplementary Fig. 39). Multifunctional OSCs potentially represent plastic enzymes that are in the process of probing evolutionary niches through duplication and diversification.

The reliability of previously characterized cladal functions to predict the product specificity of related OSCs was found to be highly variable. However, chemistries appear to be predictable on the basis of expansion of reaction pathways that are already established with the clade. Again, this is consistent with a process of duplication and diversification. There is a pressing need for more extensive functional characterization of the OSC enzyme family. More examples of sequences of known function are required to allow comprehensive investigation of sequence–function relationships and improve the accuracy and specificity of automated functional gene annotation tools. It is clear that focusing functional characterization efforts based on the wider phylogeny of OSCs has the potential to greatly accelerate our understanding of how nature controls and exploits this complicated and highly plastic cyclization process. In summary, this study provides a framework for systematic investigation of triterpene metabolic diversification and underlying enzymatic mechanisms in the plant kingdom and, ultimately, for understanding the developmental, physiological and ecological roles of this vast array of structurally diverse specialized metabolites.

## Online content

Any methods, additional references, Nature Portfolio reporting summaries, source data, extended data, supplementary information, acknowledgements, peer review information; details of author contributions and competing interests; and statements of data and code availability are available at https://doi.org/10.1038/s41589-025-02034-8.

## Methods

### Alignments and phylogenetics

Unless otherwise stated, all alignments were carried out with MAFFT (version 7.394)^48^ using the global pairwise alignment model and a maximum of 1,000 iterations. Phylogenetic trees were generated with RaXML (version 8.2)^49^ using the PROTGAMMAAUTO model, with 100 runs and bootstraps.

### Genome mining for OSCs

A total of 599 publicly available Viridiplantae genomes representing 387 species were sourced from the NCBI genome database (https://www.ncbi.nlm.nih.gov/datasets/genome/), Phytozome (https://phytozome-next.jgi.doe.gov/) and CoGe (https://genomevolution.org/coge/). Summary data for these genomes are provided in Supplementary Data 1. In some cases, both RefSeq and GenBank genome assemblies were used, as preliminary mining showed that variations in annotation pipeline led to some OSCs being missed depending on the parameters of structural gene modeling. In cases where multiple genomes assemblies were present for a single species, the genome that yielded the highest number of high-quality OSCs was used. Characterized OSCs were obtained from the literature (summarized in Supplementary Data 2). These were aligned and used to make a profile for input to Selenoprofiles (version 3)^26^.

To adapt Selenoprofiles for functionality with plant genomes and to provide filtering to produce high-quality putative sequences, the nonstandard parameters used were as follows (with explanation in italics):

p2g_filtering = len(x.protein()) > 40 or x.coverage()> 0.3

*Perform an initial filtering pass using a minimum length of 40 aa and 30% coverage with the OSC profile*.

p2g_refiltering = x.awsi_filter(awsi=0.2)

*Refilter using an average weighted sequence identity of 0.2*.

exonerate_opt = –score 300–maxintron 20000

*pply a minimum exonerate score of 300 and an allowable intron length up to 20 kbp*.

genewise_opt = -splice flat

*Use GT/AG splice models*.

blast_filtering = x.evalue < 1e-5

*Filter results with a BLAST score* < *1 × 10*^−*5*^.

This mining approach was applied to all plant genomes available, including those with structural annotations. Wherever prior annotations overlapped with the generated putative annotations, the prior annotations were always selected. For HMMER (version 3.1b2)^50^ mining, the same alignment of the OSCs listed in Supplementary Data 2 was used as a custom pHMM and a bitscore cutoff of 500 was used to select putative OSC annotations. One genome per species was chosen for subsequent analysis on the basis of the number and quality of putative enzymes found. This resulted in the generation of 2,068 unique, nonoverlapping putative OSC sequences from 304 genomes representing 258 species.

Before alignment, high-quality putative protein sequences were filtered by requiring a minimum length of 650 aa and the removal of pseudogenes as flagged by Selenoprofiles (that is, if frameshifts or indels were required to generate the alignment of protein profile to nucleotides). In summary, the mining process produced 1,405 high-quality OSC sequences, as defined by a length of ≥650 aa and, using a custom profile of characterized OSCs, either a bitscore of >500 (for HMMER mining) or a filtered Selenoprofiles homolog result (according to the parameters above).

### OSC profiling

pHMMs of representative sequences within each OSC group were generated by selecting up to 100 representative samples across each clade followed by alignment and building with HMMER. These profiles were then used for on-the-fly characterization of OSC sequences by choosing the profile that most closely matched the OSC in question. A minimum alignment span of 450 aa was selected, which was found to maintain accuracy whilst allowing putative classification of partial sequences not included in the phylogenetic analysis. It should be noted that not all groups were monophyletic; thus, accuracy was reduced when attempting to assign proteins to specific groups on the basis of sequence similarity alone for these groups.

### BGC mining

plantiSMASH-release^36^ and its dependencies were installed according to the developer’s instructions (https://bitbucket.org/satriaphd/plantismash-release/src/master/). Genomes with structural annotations were put forward for BGC mining by plantiSMASH. Standard parameters were used, minus the maximum limit of 9,999 contigs. HTML and JavasScript outputs were parsed by Python. OSCs across the whole genome were defined by alignment to the Pfam profiles ‘SQHop_cyclase_C’ (PF13243) or ‘SQHop_cyclase_N’ (PF13249), with the highest-scoring sequence taken forward where multiple isoforms were present in the annotation.

### Gene synthesis

OSCs 1–20 were selected manually, choosing those that appeared to be of interest on the basis of phylogenetic placement and branch length (Supplementary Table 1). Where possible, public RNA-sequencing data were screened to validate the expression of the OSC in the source plant. The native coding sequences of each gene flanked by the 5′ sequence (GGGGACAAGTTTGTACAAAAAAGCAGGCTTA) and 3′ sequence (TACCCAGCTTTCTTGTACAAAGTGGTCCCC) were synthesized as double-stranded linear gene fragments (eBlocks gene fragments service, Integrated DNA Technologies). OSC9 mutants: The amino acid sequence of each mutant was used to generate a coding sequence which was codon-optimized for plant expression and gene synthesis. These were synthesizedflankedbythe 5′ sequence(GGGGACAAGTTTGTACAAAAAA-GCAGGCTTA) and 3′ sequence (TACCCAGCTTTCTTGTACAAAGTGGTCCCC) as clonal genes (pTwist, Amp High Copy, Twist Bioscience).

### Cloning

The synthesized gene products were cloned into the pDONR207 vector using BP clonase II enzyme mix (Invitrogen) according to the manufacturer’s instructions. These entry vectors were verified for the correct gene insert by Sanger sequencing (Eurofins Genomics). Subsequently, these were cloned into pEAQ-HT-DEST1 (ref. 37) using LR clonase II enzyme (Invitrogen) according to the manufacturer’s instructions. These expression constructs were transformed into chemically competent *A. tumefaciens* (strain LBA4404) by flash-freezing in liquid nitrogen.

### Transient expression in *N. benthamiana*

Transient expression was carried out as described previously^38,39^. *A. tumefaciens* strains were streaked from glycerol stock cultures onto selective Luria–Bertani (LB) agar plates (50 μg ml^−1^ rifampicin, 50 μg ml^−1^ kanamycin and 100 μg ml^−1^ streptomycin) and incubated overnight at 28 °C. These cultured streaks were used to individually inoculate 10 ml of selective LB medium (50 μg ml^−1^ rifampicin, 50 μg ml^−1^ kanamycin and 100 μg ml^−1^ streptomycin) and incubated overnight at 28 °C with shaking. Cells were pelleted by centrifugation and resuspended in freshly prepared MMA buffer (10 mM 2-(*N*-morpholino)-ethanesulphonic acid pH 5.6 (KOH), 10 mM MgCl_2_, 150 μM 3′5′-dimethoxy-4′-acetophenone) at an optical density at 600 nm of 0.2. Each prepared suspension was mixed with an equal volume of one carrying the expression construct for tHMGR. For each condition, three leaves of 6-week-old *N. benthamiana* plants were fully infiltrated by hand using a needless syringe. Following infiltration, the plants were grown for an additional 5 days under greenhouse conditions (25 °C with 16 h of lighting). The leaves were then individually isolated and freeze-dried.

### Extraction and GC–MS analysis

Each freeze-dried leaf was ground into a fine powder. Approximately 20 mg of leaf powder was transferred to a 1.5-ml Eppendorf tube and the weight transferred recorded. The leaf powder was suspended in 300 μl of ethyl acetate containing coprostanol as an IS (the concentration of coprostanol was 100 ppm for the initial functional screening and 200 ppm for the analysis of the OSC9 mutagenesis experiments). The suspensions were heated at 55 °C with sonication by floating the Eppendorf tubes in a sonicating water bath. After 1 h, the leaf powder was pelleted by centrifugation. The clarified supernatant was transferred to an HPLC vial for direct analysis by GC–MS. GC parameters were as follows: injection, 1 μl; pulsed split (20:1 split ratio), 30 psi; inlet temperature, 250 °C; column, Phenomenex Zebron ZB5-HT Inferno; carrier gas, He; flow rate, 1.2 ml min^−1^; temperature gradient, 0 min (170 °C), 2 min (170 °C), 8.5 min (300 °C) and 20 min (300 °C); instrument, Agilent 7890B. MS parameters were as follows: ionization, EI (70 eV); detector, Agilent 5977A; scans, 7.2 scans per s at 60–800 *m*/*z*. Data collection was carried out in MassHunter Workstation (version 10.0) Estimation of abundance relative to the IS was calculated as follows: (area analyte/area IS) × concentration IS (μg mg^−1^ dry weight). For the functional screening experiments, this was calculated from integration of TIC peaks at half peak height (to allow untargeted analysis). For the targeted analysis of the relative quantities of euphanes and tirucallanes between OSC9 mutants, this was calculated from integration of full peak areas from generated extracted ion chromatograms, 411 for compounds **5, 7, 11** and **18** and 388 for the IS (coprostanol).

### Isolation and identification of 3β,20-dihydroxylupane (39) in the product profile of OSC19

Through isolation and NMR analysis, the chromatographic peaks at relative retention times of 1.68 and 3.57 min in the product profile OSC19 were found to both correspond to 3β,20-dihydroxylupane (**39**), with these chromatographic peaks representing degradation artifacts during GC–MS analysis. Transient expression of OSC19, as described above, was repeated in 60 leaves. The isolated and freeze-dried leaves were ground and the resulting dry leaf powder was subjected to batchwise pressurized solvent extraction with ethyl acetate (instrument, Büchi SpeedExtractor E-914; extraction cell size, 120 ml; cycles, three cycles at 100 °C and 130 bar of pressure; hold times, cycle 1 for 0 min and cycles 2 and 3 for 5 min; dispersant, one part by volume quartz sand). Combined extracts were concentrated to dryness by rotatory evaporation under vacuum and dissolved in a minimum volume of ethanol. To this, 50 ml of basic ion-exchange resin (Ambersep 900 OH) was added and the mixture was shaken at room temperature. Every 30 min, an additional 50 ml of basic ion-exchange resin was added until the extract changed from green to murky orange in color. The extract was collected by filtration through a short column of diatomaceous earth and subjected to repeated automated flash chromatography using a Biotage Isolera instrument. The first flash chromatography proceeded with the following parameters: loading method, dry-loaded on silica gel; column, 10-g SnapUltra silica Biotage cartridge; solvent A, hexane; solvent B, ethyl acetate; gradient, 0% B to 100% B over 720 ml; flow rate, 36 ml min^−1^, with 20-ml fractions. The second flash chromatography proceeded with the following parameters: loading method, dry-loaded on silica gel; column, 10-g SnapUltra silica Biotage cartridge; solvent A, hexane; solvent B, ethyl acetate; gradient: 0% B to 50% B over 720 ml; flow rate, 36 ml min^−1^, with 20-ml fractions. The third flash chromatography proceeded with the following parameters: loading method, dry-loaded on silica gel; column, 10-g SnapUltra silica Biotage cartridge; solvent A, hexane; solvent B, ethyl acetate; gradient, 0% B to 100% B over 720 ml; flow rate, 36 ml min^−1^, with 20-ml fractions. Fractions were monitored by GC–MS. The chromatographic peaks at relative retention times of 1.68 and 3.57 min were inseparable. A sample containing these chromatographic peaks was isolated in a trace amount as a white solid and subjected to ^1^H-NMR analysis in CDCl_3_ (600 MHz). This showed the sample to contain one triterpene that matched the literature for 3β,20-dihydroxylupane (**39**) (Supplementary Fig. 8).

### Confirmation of the presence of bauerenol (28) and taraxasterol (29) in the product profiles of OSC6 and OSC7 through a metabolomic NMR approach

The EI mass spectra of **28** and **29** were consistent with the known triterpenes bauerenol and taraxasterol, respectively (Supplementary Figs. 26 and 27). The suspected presence of the compounds bauerenol (**28**) and taraxasterol (**29**) were confirmed in the product profiles of OSC6 and OSC7, respectively, by a metabolomic NMR approach. For each OSC, transient expression was carried out using six leaves. The isolated and freeze-dried leaf material was ground and the resulting dry leaf powder (approximately 600 mg) was extracted with 20 ml of ethanol at 55 °C with sonication for 99 min. Next, 10 ml of basic ion-exchange resin (Ambersep 900 OH) was added and the mixture was shaken at 60 °C until the extract changed from green to orange in color (approximately 30 min). The extract was filtered and dried onto 1 g of silica gel (40–75-μm particle size, 70-Å pore size) by rotatory evaporation under vacuum before elution with 10 ml of 15% ethyl acetate in hexane. The resulting eluent was treated with 1 g of decolorizing charcoal and recovered by filtration. Solvent was removed by evaporation under a stream of nitrogen to give a colorless oil. The oil was dissolved in 500 μl of CDCl_3_ and concentrated under of stream of nitrogen three times. Finally, the recovered oil was dissolved again in 500 μl of CDCl_3_ and subjected to a 32-scan distortionless enhancement by polarization transfer (DEPT)-edited heteronuclear single quantum coherence (HSQC) NMR experiment (^1^H, 600 MHz; ^13^C, 125 MHz). Characteristic correctly phased HSQC cross-peaks corresponding to the ^1^H and ^13^C chemical shifts for the alkene of each compound were identified, along with other identifiable signals (Supplementary Figs. 9 and 10).

### Isolation and identification of kalanchoeol (24a) and spirokalanchoeol (24b) in the product profile of OSC16

Transient expression of OSC16 was carried out using 90 leaves. The isolated and freeze-dried leaves were ground and the resulting dry powder subjected to batchwise pressurized solvent extraction with ethyl acetate (instrument, Büchi SpeedExtractor E-914; extraction cell size, 120 ml; cycles, three cycles at 100 °C and 130 bar of pressure; hold times, cycle 1 for 0 min and cycles 2 and 3 for 5 min; dispersant, one part by volume quartz sand). Combined extracts were concentrated to dryness by rotatory evaporation under vacuum and dissolved in a minimum volume of ethanol. To this, 50 ml of basic ion-exchange resin (Ambersep 900 OH) was added and the mixture shaken at room temperature. Every 30 min, an additional 50 ml of basic ion-exchange resin was added until the extract changed from green to murky orange in color. The extract was collected by filtration through a short column of diatomaceous earth and subjected to repeated automated flash chromatography (using a Biotage Isolera instrument). The first flash chromatography proceeded with the following parameters: loading method, dry-loaded on silica gel; column, 10-g SnapUltra silica Biotage cartridge; solvent A, hexane; solvent B, ethyl acetate; gradient, 0% B to 100% B over 720 ml; flow rate, 36 ml min^−1^, with 20-ml fractions. The second flash chromatography proceeded with the following parameters: loading method, dry-loaded on silica gel; column, 10-g SnapUltra silica Biotage cartridge; solvent A, hexane; solvent B, ethyl acetate; gradient: 0% B to 25% B over 720 ml; flow rate, 36 ml min^−1^, with 20-ml fractions. The third flash chromatography proceeded with the following parameters: loading method, dry-loaded on silica gel; column, 10-g SnapUltra silica Biotage cartridge; solvent A, hexane; solvent B, ethyl acetate; gradient, 0% B to 25% B over 720 ml; flow rate, 36 ml min^−1^, with 20-ml fractions. Fractions were monitored by GC–MS. A 28 mg sample enriched in **24a** and **24b** was isolated as a pale-yellow oil. This sample was dissolved in 1 ml of tetrahydrofuran and subjected to preparative HPLC (Instrument, Agilent Technologies Infinity system equipped with a 1290 Infinity II fraction collector and a 1290 Infinity II preparative pump; column, 250 × 21.2-mm Luna 5-μm C18(2), 100 Å (Phenomonex); flow rate, 25 ml min^−1^; gradient, isocratic 100% acetronitrile containing 0.1% formic acid; run time, 30 min; injection volume, 1 ml; fraction size, 21 ml). Fractions were monitored by GC–MS. Under these conditions, **24a** and **24b** separated and were collected in different fractions. After solvent removal under a steam of nitrogen, both were isolated in trace amounts as white solids. These were directly dissolved in CDCl_3_ and subjected to extensive NMR analysis (^1^H, ^13^C, correlation spectroscopy, DEPT-135, DEPT-edited HSQC and heteronuclear multiple bond correlation) (Supplementary Figs. 29–36).

### In silico docking

A homology model of OSC9 was generated using the Phyre2 server^51^ operating in normal mode. The top-ranked model was selected for further study (Supplementary Data 5). The template structure for this model was the OSC human lanosterol synthase in complex with lanosterol (Protein Data Bank (PDB) 1W6K) (98% alignment coverage, 38% sequence identity). Next, lanosterol from PDB 1W6K was combined with the OSC9 model. OSC9 residues within 5 Å of the transplanted lanosterol were identified (F126, W216, W254, Y256, C257, T260, E368, T408, F409, W414, V479, A481, C482, V530, F549, I552, T561, W609, Y615, S724, Y725, M726, L731 and Y733). These were selected as flexible residues for subsequent in silico docking using AutoDock Vina^52^. A 3D model of the dammarenyl cation was built and then geometry-optimized by molecular mechanics (force field, MMFF94s; number of steps, 500; algorithm, steepest descent; convergence, 10^−7^) using Avogadro (version 1.2.0)^53^. The rigid and flexible receptor files of OSC9 and the ligand file of the dammarenyl cation for use with Autodock Vina (version 1.1.2) in flexible mode were prepared using the accompanying AutoDock Tools package following the default parameters. Docking was performed using the following configuration parameters: center *x* = 31.09, center *y* = 70.677, center *z* = 6.968, size *x* = 30, size *y* = 30, size *z* = 30, exhaustiveness = 24, seed = 166819220, energy range = 10 and num modes = 10. Inspection of the lowest-energy binding model (affinity = −16.5 kcal mol^−1^) showed the dammarenyl cation to be correctly orientated in the active site compared to lanosterol in PDB 1W6K and, thus, it was selected for use in this study (Supplementary Data 6).

### Reporting summary

Further information on research design is available in the Nature Portfolio Reporting Summary linked to this article.

## Data availability

The 16 functional OSC sequences described in the paper were submitted to NCBI GenBank with the following accession codes: OSC1 (*Aquilaria agallochum*), PV548881; OSC2 (*Elaeis guineensis*), PV548882; OSC4 (*A. officinalis*), PV548883; OSC6 (*J. regia*), PV548884; OSC7 (*I. nil*), PV548885; OSC8 (*G. bardense*), PV548886; OSC9 (*I. nil*), PV548887; OSC10 (*Daucus carota*), PV548888; OSC11 (*Pyrus* × *bretschneideri*), PV548889; OSC12 (*Vigna radiata*), PV548890; OSC14 (*Capsella bursa-pastoris*), PV548891; OSC15 (*Arabis alpina*), PV548892; OSC16 (*K. fedtschenkoi*), PV548893; OSC17 (*H. lupulus*), PV548894; OSC19 (*T. hassleriana*), PV548895; OSC20 (*B. rapa*), PV548896. The set of 1,405 mined full-length OSCs is provided in Supplementary Data 7. Data are available from the corresponding authors upon request. Source data are provided with this paper.

## Extended Data

## Supplementary Material

The online version contains supplementary material available at https://doi.org/10.1038/s41589-025-02034-8.

Extended Data Fig. 1

Extended Data Fig. 2

Extended Data Fig. 3

Extended Data Fig. 4

Extended Data Fig. 5

Extended Data Fig. 6

Extended Data Fig. 7

Extended Data Fig. 8

Extended Data Fig. 9

Extended Data Fig. 10

SI Data S1 & S2

SI Data S3

SI Data S4

Source Data Ext Fig. 3

Source Data Ext Fig. 9

## Figures and Tables

**Fig. 1:**

Generation of diverse triterpene scaffolds by OSCs through cyclization of the linear precursor, 2,3-oxidosqualene. **a**, The protosteryl–dammarenyl cation dichotomy in triterpene reaction pathways and their relationship to lanosterol and euphol. **b**, Examples of structural diversity originating from E-ring expansion of the dammarenyl cation, showing the relationship between the final scaffolds and the shared intermediatory carbocations. **c**, Carbon numbering and ring nomenclature used in this Article. The red asterisk in **b** indicates a cation referred to in Fig. 5.
