# Supplementary figures and images for: Large-scale mining of plant genomes unlocks the diversity of oxidosqualene cyclases
Source: Nat Chem Biol. Author manuscript; Available in PMC 2026 Feb 3. (PMC12858397; doi:10.1038/s41589-025-02034-8)

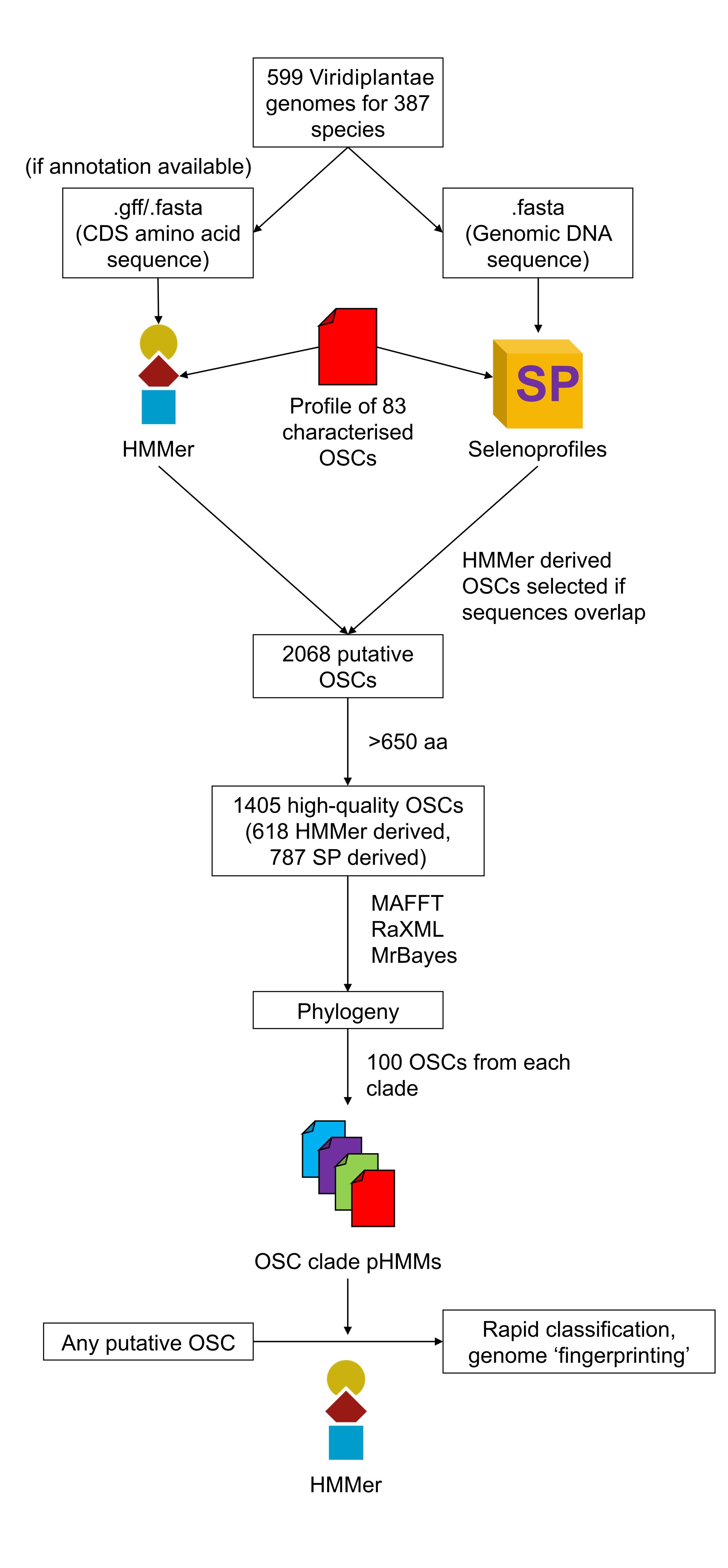

Supplement: Extended Data Fig. 1 [file EMS212204-supplement-Extended_Data_Fig__1.jpg]

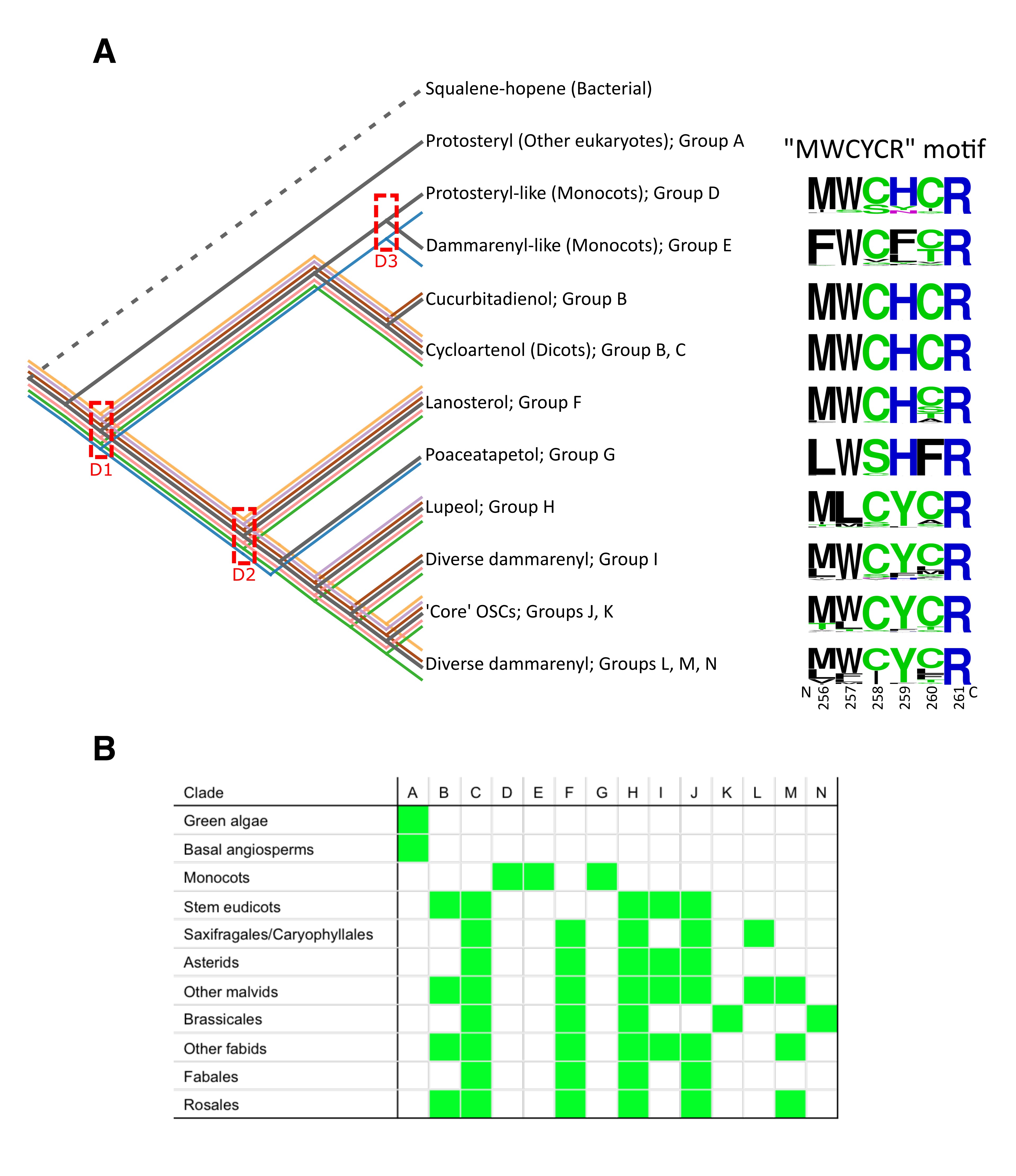

Supplement: Extended Data Fig. 2 [file EMS212204-supplement-Extended_Data_Fig__2.jpg]

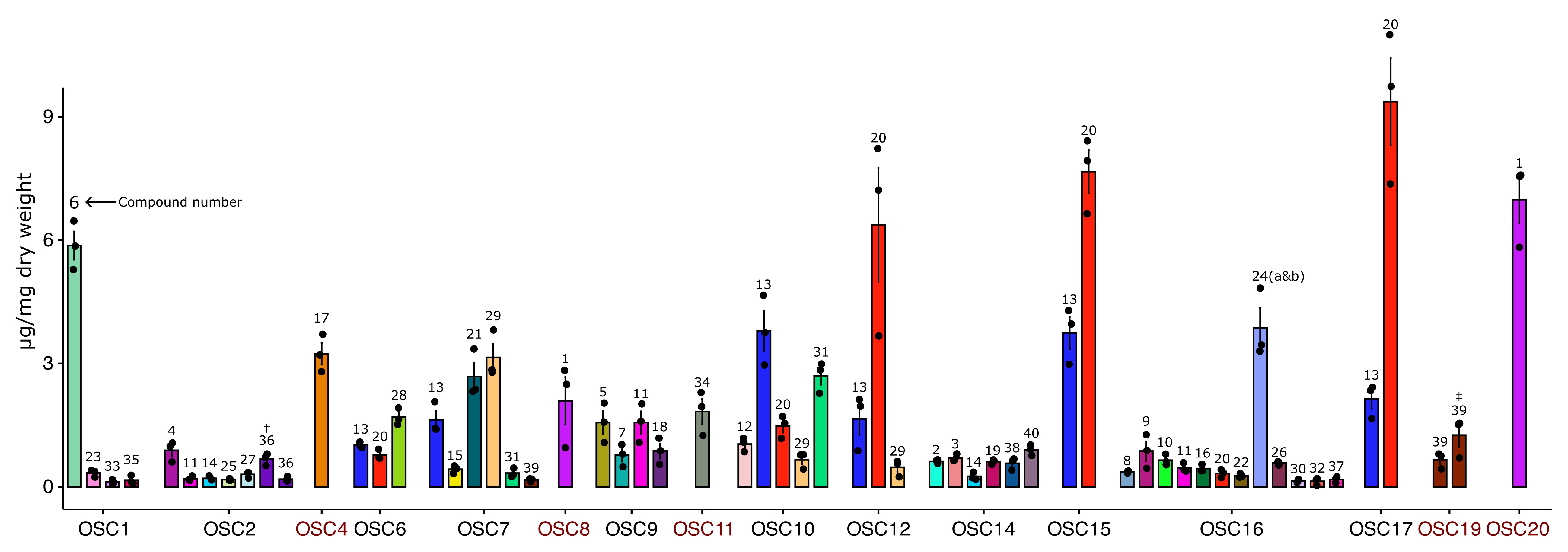

Supplement: Extended Data Fig. 3 [file EMS212204-supplement-Extended_Data_Fig__3.jpg]

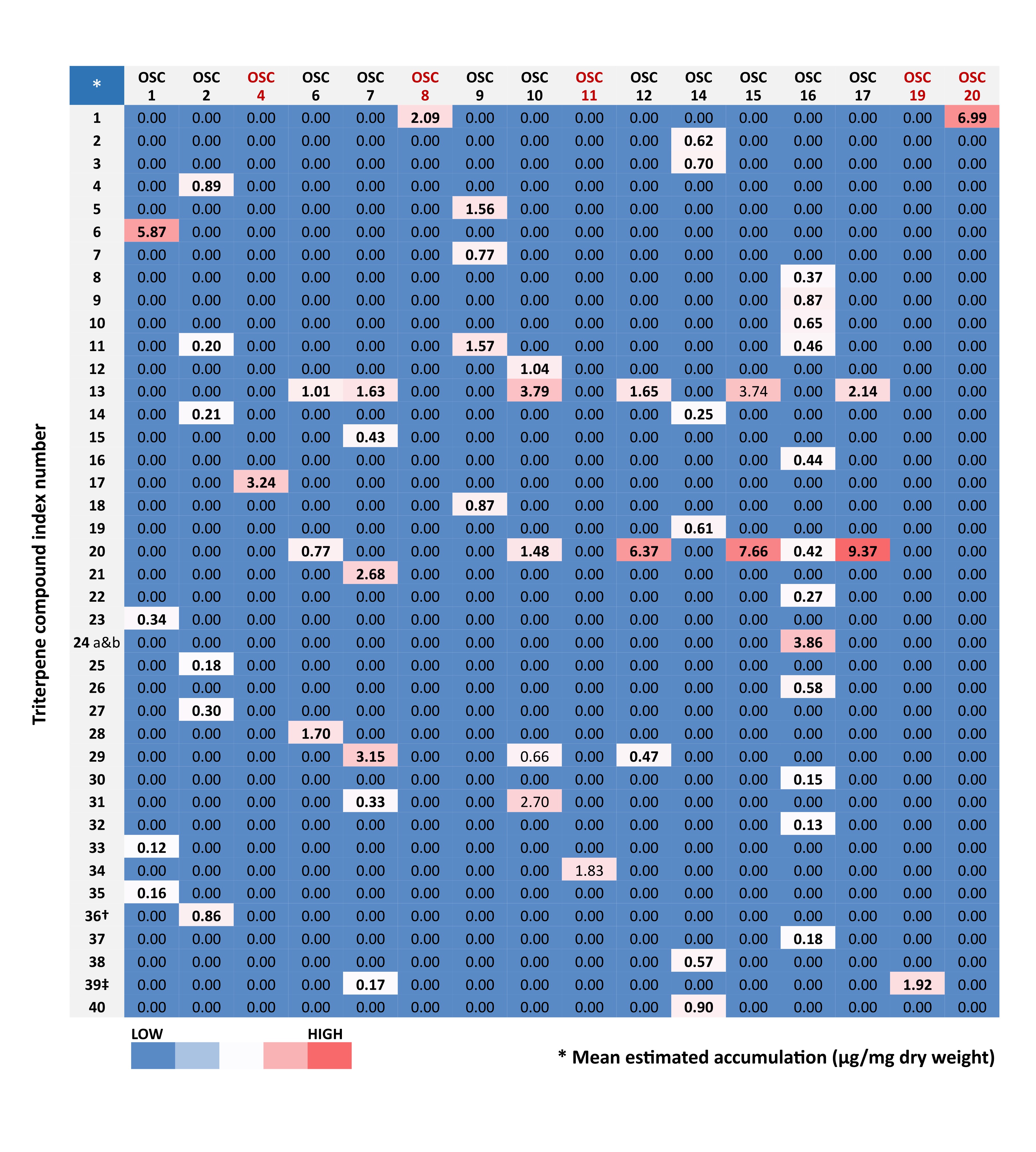

Supplement: Extended Data Fig. 4 [file EMS212204-supplement-Extended_Data_Fig__4.jpg]

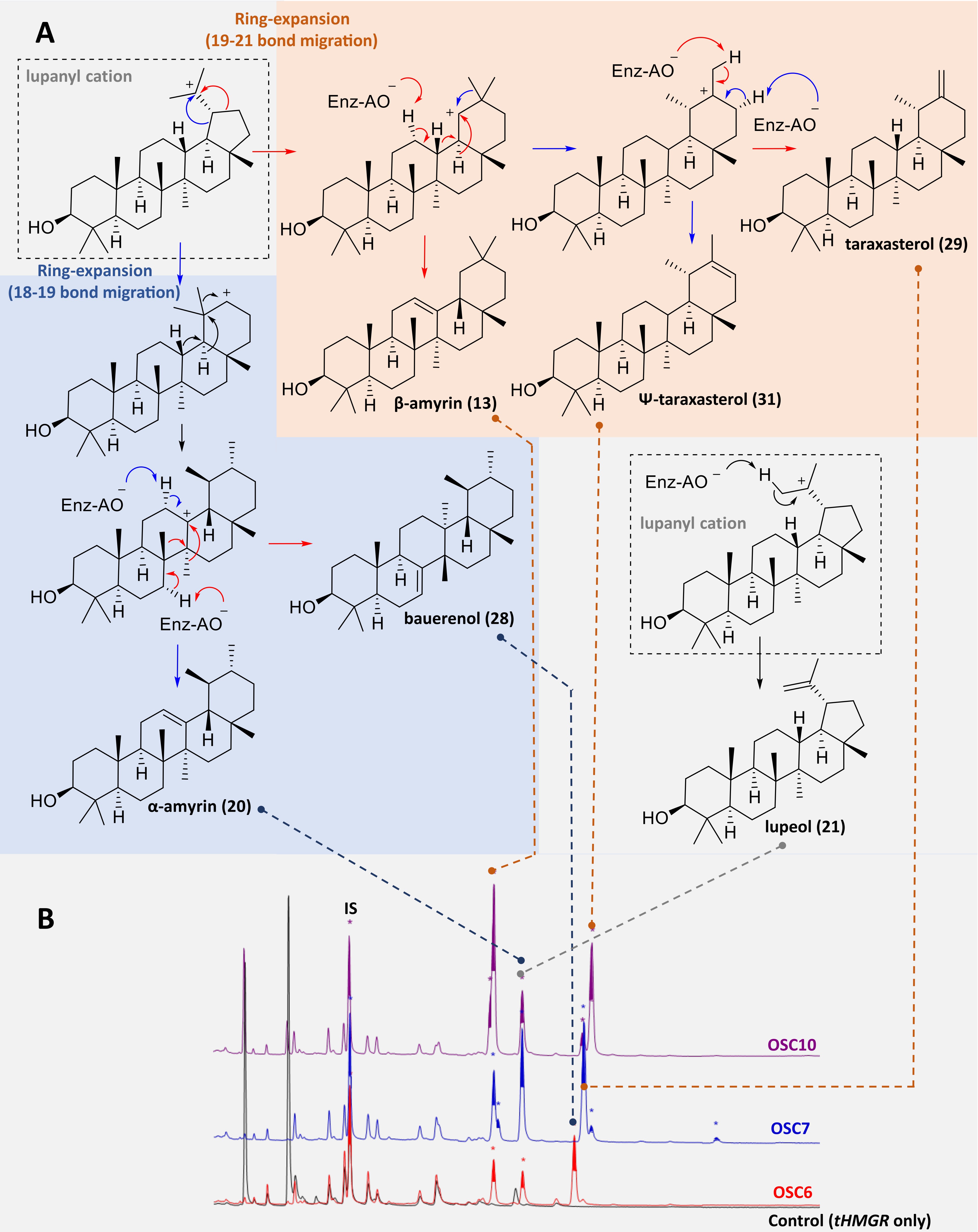

Supplement: Extended Data Fig. 5 [file EMS212204-supplement-Extended_Data_Fig__5.jpg]

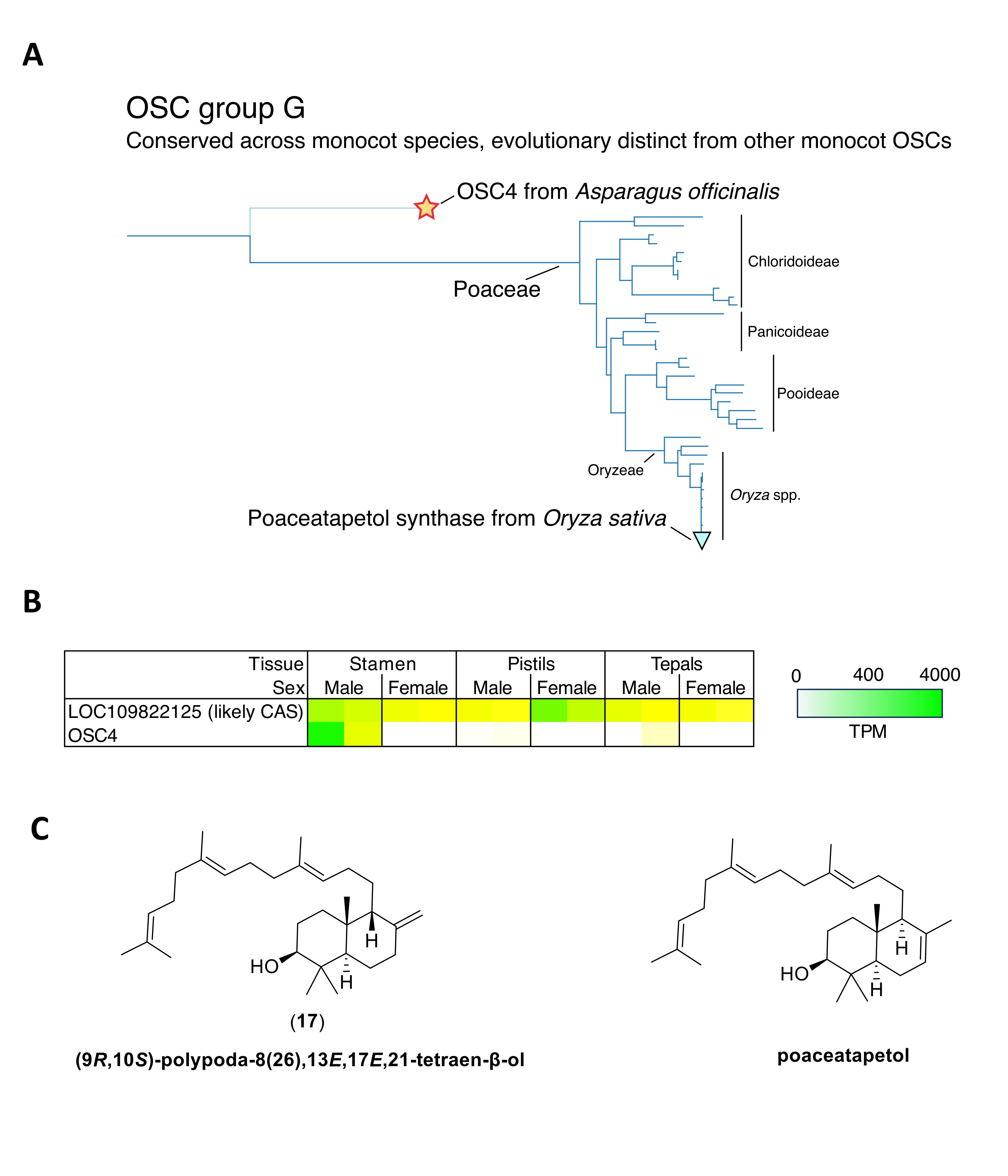

Supplement: Extended Data Fig. 6 [file EMS212204-supplement-Extended_Data_Fig__6.jpg]

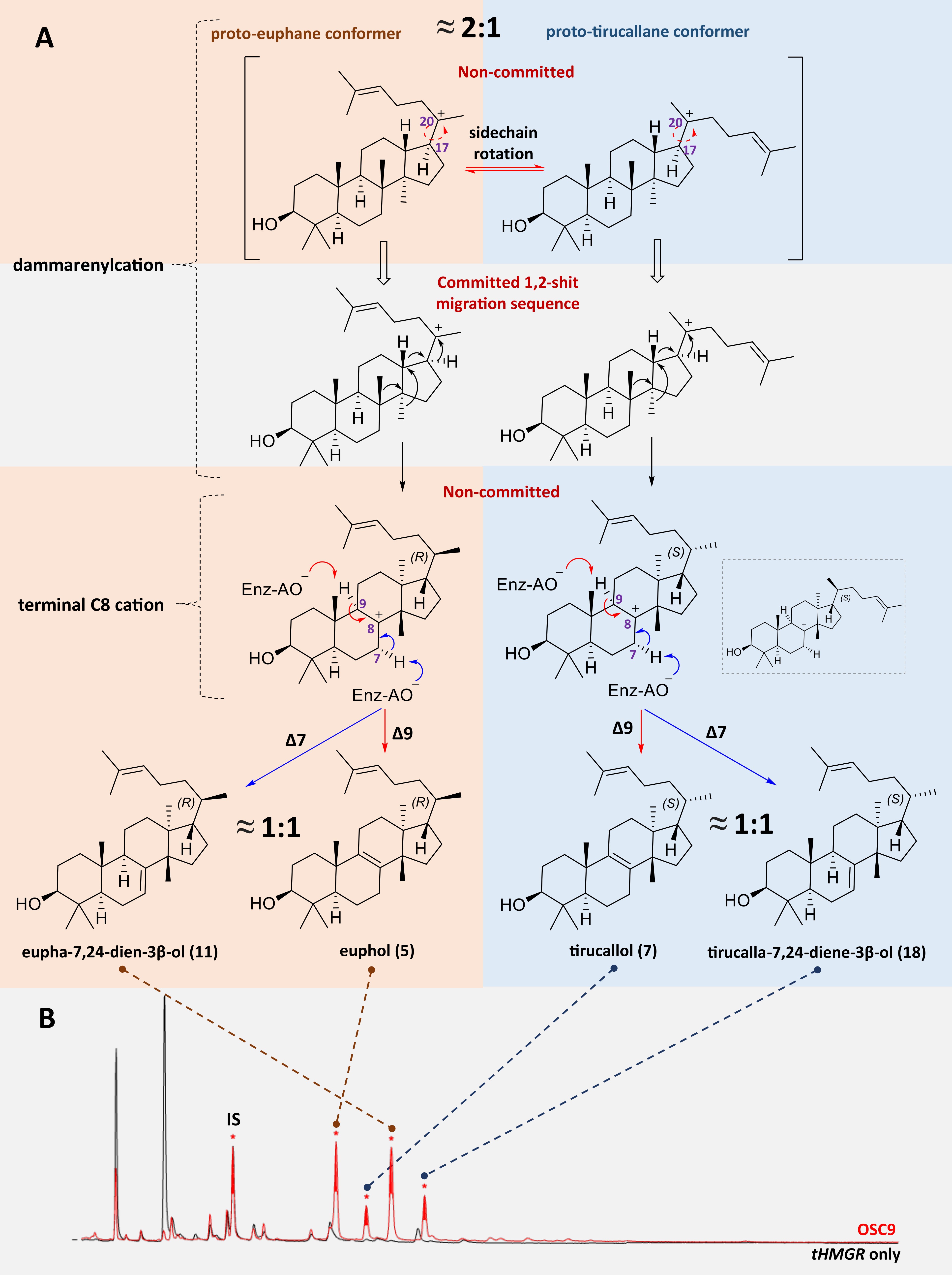

Supplement: Extended Data Fig. 7 [file EMS212204-supplement-Extended_Data_Fig__7.jpg]

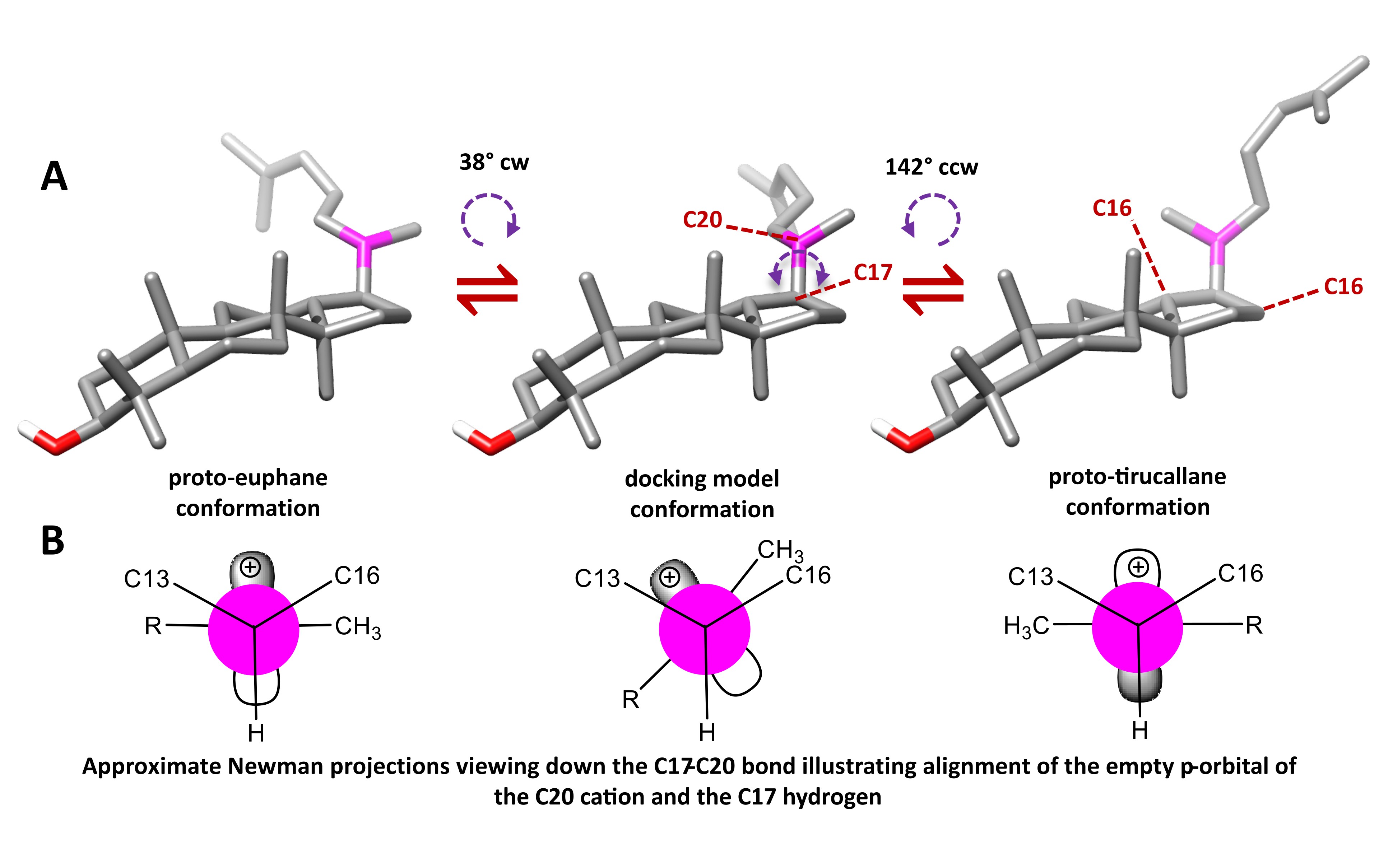

Supplement: Extended Data Fig. 8 [file EMS212204-supplement-Extended_Data_Fig__8.jpg]

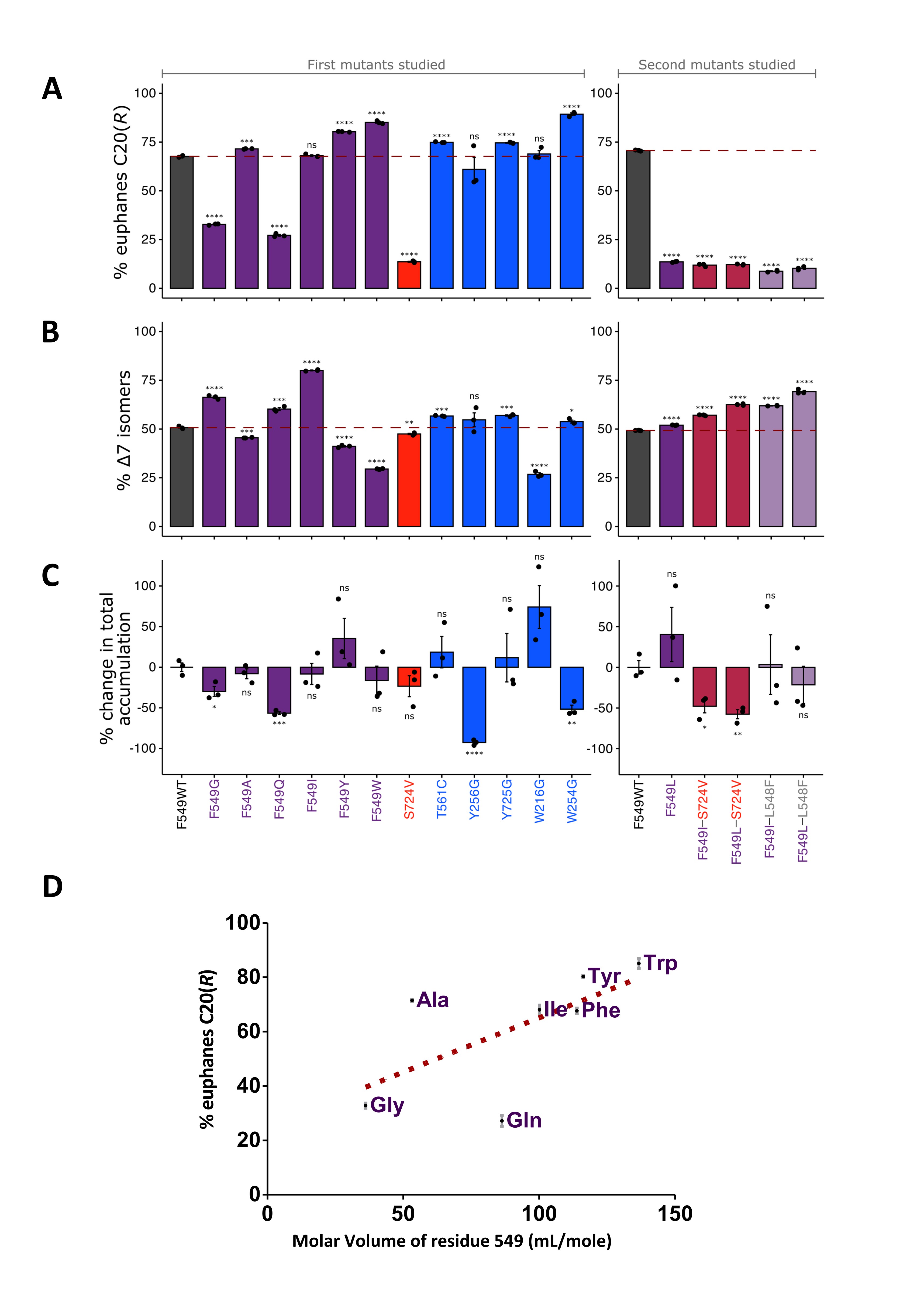

Supplement: Extended Data Fig. 9 [file EMS212204-supplement-Extended_Data_Fig__9.jpg]

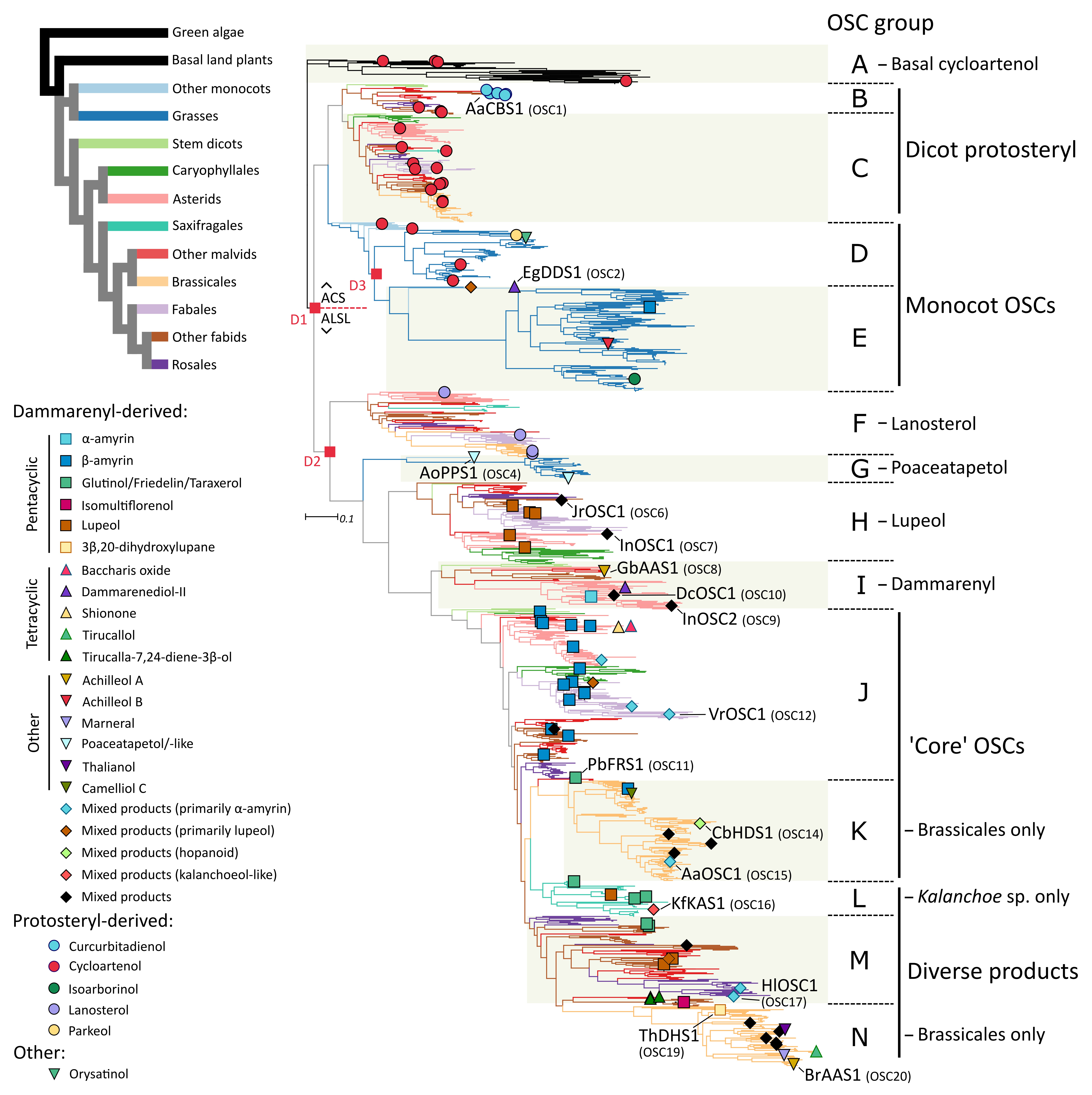

Supplement: Extended Data Fig. 10 [file EMS212204-supplement-Extended_Data_Fig__10.jpg]
