# Supplementary material for: Large-scale mining of plant genomes unlocks the diversity of oxidosqualene cyclases
Source: Nat Chem Biol. 2025 Oct 6;22(2):249–59. doi: 10.1038/s41589-025-02034-8 (PMC12858397; doi:10.1038/s41589-025-02034-8)
Supplement: Supplementary file 1 [file 41589_2025_2034_MOESM1_ESM.pdf]

nature chemical biology

Supplementary information

https://doi.org/10.1038/s41589-025-02034-8

Large-scale mining of plant genomes unlocks the diversity of oxidosqualene cyclases

In the format provided by the authors and unedited

Table of Contents

Supplementary Fig. 1 | Order-level taxonomy of Viridiplantae species used in this study.

Supplementary Fig. 2 | The structures of compounds named in Fig. 2.

Supplementary Fig. 3 | OSC 'fingerprinting' for species across the Viridiplantae.

Supplementary Fig. 4 | Examples of OSC 'fingerprinting' in different plant clades.

Supplementary Fig. 5 | Phylogenetic tree showing the mean copy number of OSCs per species for the children at each node.

Supplementary Fig. 6 | Enrichment of different OSC groups in biosynthetic gene clusters (BGCs).

Supplementary Fig. 7 | Alignment of selected OSC sequences.

Supplementary Fig. 8 | ^{1}H NMR spectrum of a compound (**39**) sample isolated from large-scale expression of OSC19 (600 MHz, $CDCl_3$).

Supplementary Fig. 9 | DEPT-Edited-HSQC spectra confirming presence of baurenol.

Supplementary Fig. 10 | DEPT-Edited-HSQC spectra confirming presence of taraxasterol.

Supplementary Fig. 11 | GC-MS comparison of OSC1 product profile and that of expression of known cucurbitadienol synthase (CPQ).

Supplementary Fig. 12 | GC-MS comparison of OSC2 product profile and that of expression of known dammarenediol II synthase.

Supplementary Fig. 13 | GC-MS comparison of OSC6 product profile with authentic standards.

Supplementary Fig. 14 | GC-MS comparison of OSC7 product profile with authentic standards.

Supplementary Fig. 15 | GC-MS comparison of OSC9 product profile with authentic standards.

Supplementary Fig. 16 | GC-MS comparison of OSC10 product profile with authentic standards.

Supplementary Fig. 17 | GC-MS comparison of OSC11 product profile with an authentic standard.

Supplementary Fig. 18 | GC-MS comparison of OSC12 product profile with authentic standards.

Supplementary Fig. 19 | GC-MS comparison of OSC14 product profile with an authentic standard.

Supplementary Fig. 20 | GC-MS comparison of OSC15 product profile with authentic standards.

Supplementary Fig. 21 | GC-MS comparison of OSC17 product profile with authentic standards.

Supplementary Fig. 22 | Putative assignment of (**1**).

Supplementary Fig. 23 | Putative assignment of (**4**).

Supplementary Fig. 24 | Putative assignment of (**11**).

Supplementary Fig. 25 | Putative assignment of (**17**).

Supplementary Fig. 26 | EI mass spectrum of bauerenol (**28**) from this work.

Supplementary Fig. 27 | Putative assignment of (**31**).

Supplementary Fig. 28 | Putative assignment of (**40**).

Supplementary Fig. 29 | ^{13}C (150 MHz) & ^{1}H (600 MHz) δ assignments spirokalanchoeol (**24b**).

Supplementary Fig. 30 | ^{1}H spectrum spirokalanchoeol (**24b**).

Supplementary Fig. 31 | ^{13}C & DEPT-135 spectrum spirokalanchoeol (**24b**).

Supplementary Fig. 32 | DEPT-edited-HSQC spectrum spirokalanchoeol (**24b).**

Supplementary Fig. 33 | ^{13}C (150 MHz) & ^{1}H (600 MHz) δ assignments of kalanchoeol (**24a**).

Supplementary Fig. 34 | ^{1}H spectrum kalanchoeol (**24a**).

Supplementary Fig. 35 | ^{13}C & DEPT-135 spectrum kalanchoeol (**24a**)

Supplementary Fig. 36 | DEPT-edited-HSQC spectrum kalanchoeol (**24a).**

Supplementary Fig. 37 | Phylogeny of OSC16 (KfKAS).

Supplementary Fig. 38 | Multiple sequence alignment of the amino acid sequences for OSC9 and functional relatives.

Supplementary Fig. 39 | Functional analysis of 8 previously characterised OSCs using the workflow in this paper.

Supplementary Table 1 | Summary of selected OSCs from genome mining detailed this study and their products.

Supplementary References

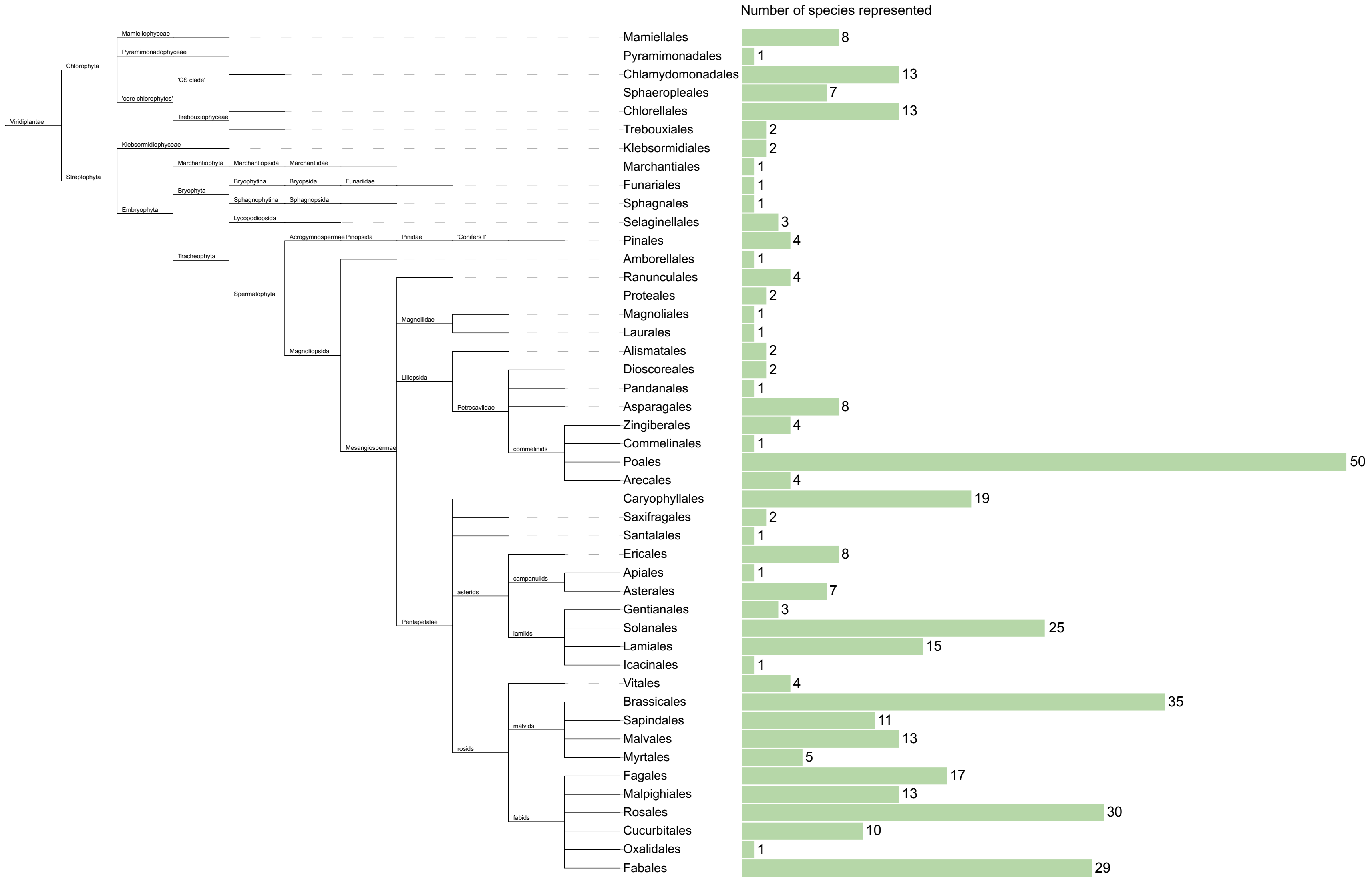

Supplementary Fig. 1 | Order-level taxonomy of Viridiplantae species used in this study. Bar charts represent the number of species present from each order. Species with multiple representative genomes are counted as one.

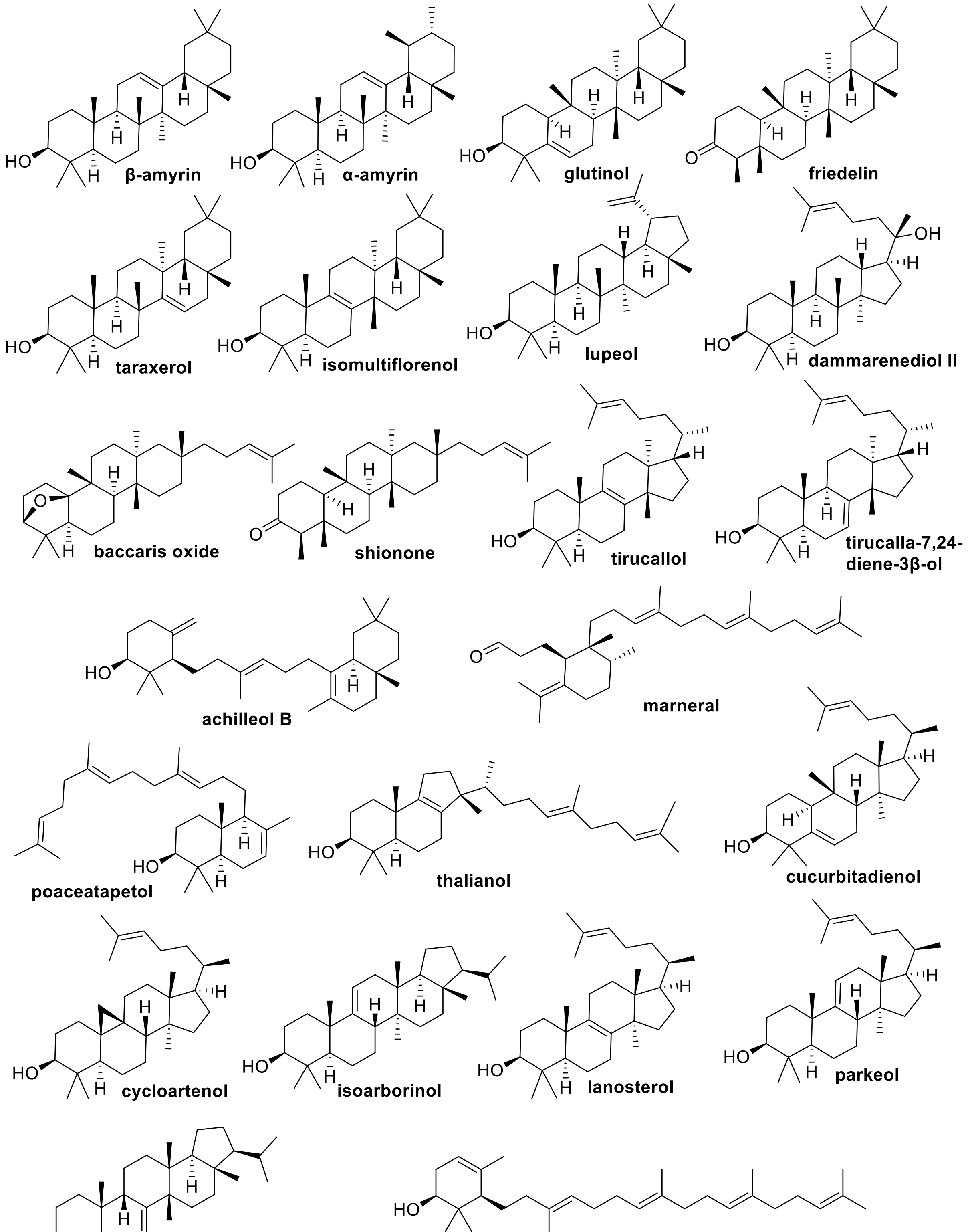

Supplementary Fig. 2 | The structures of compounds named in Fig. 2.

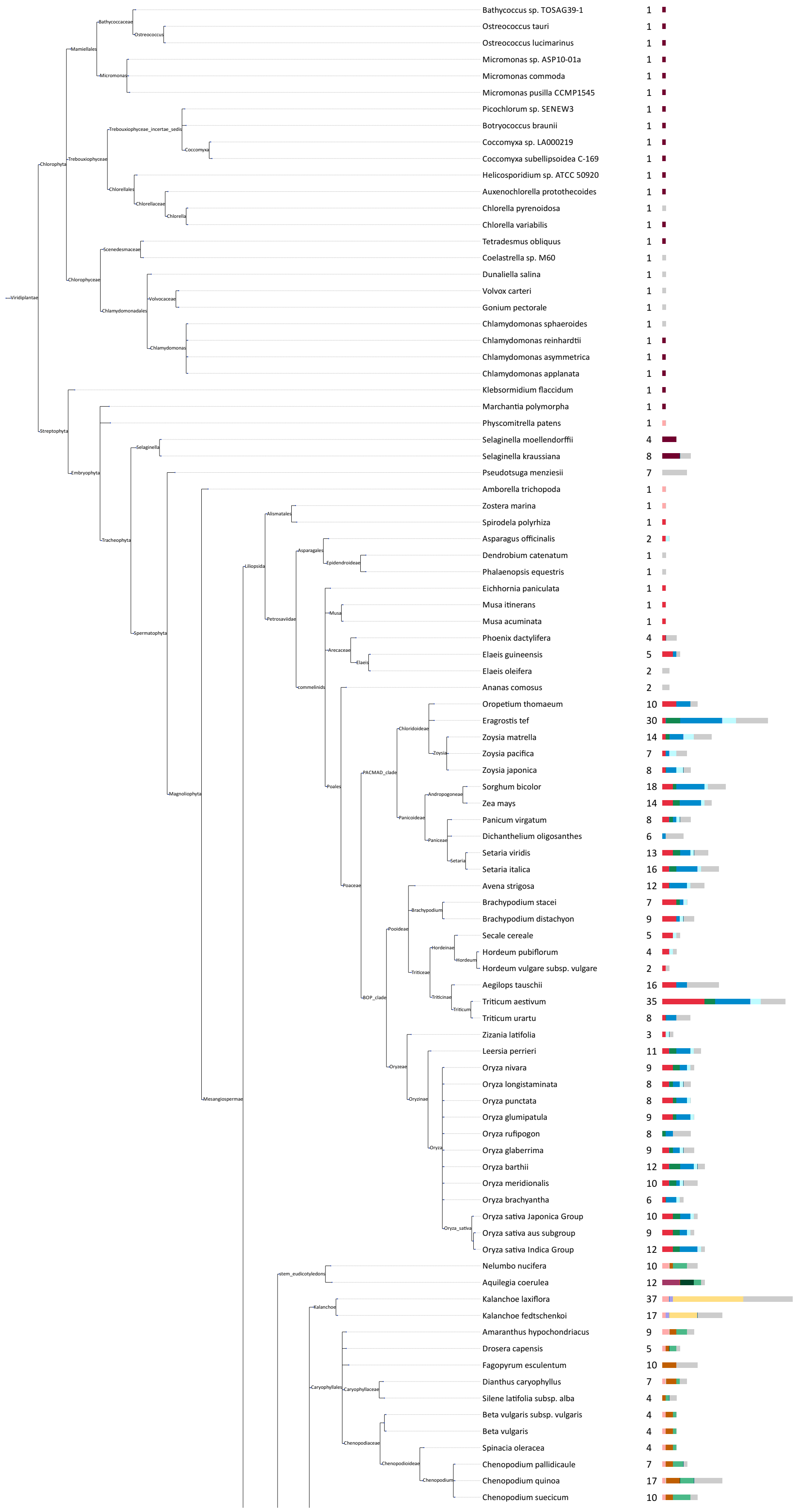

Supplementary Fig. 3 (continued below)

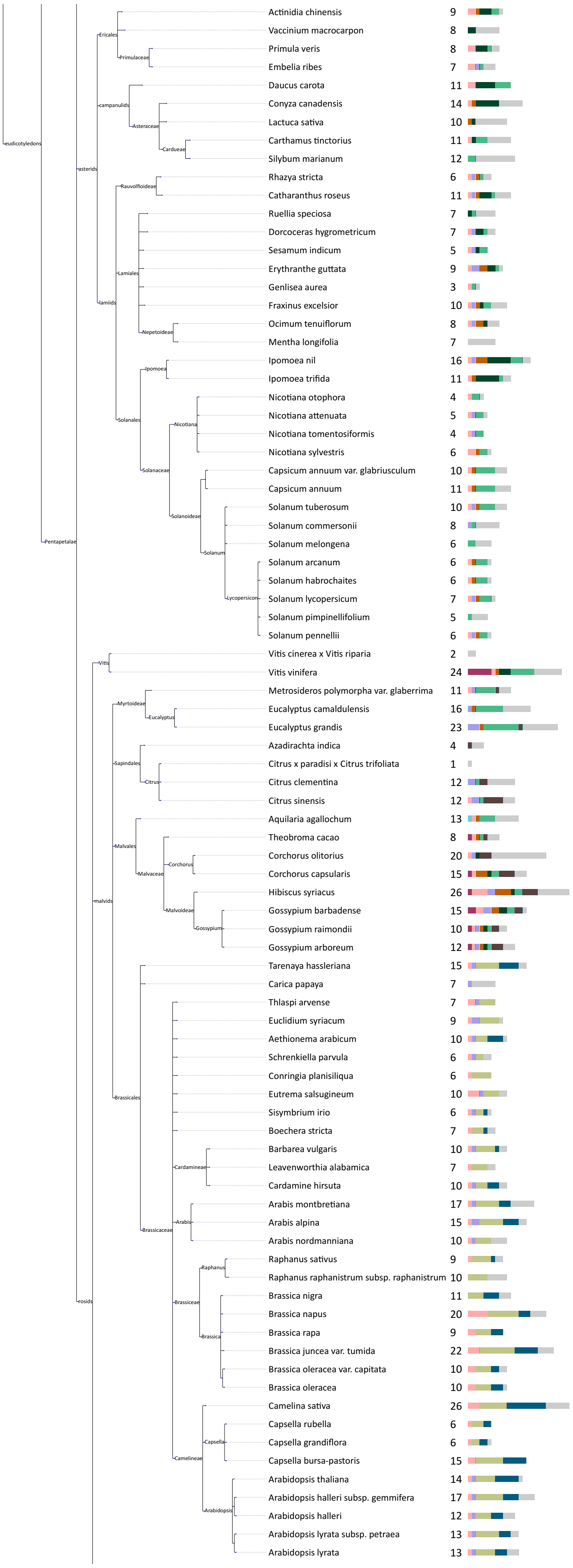

Supplementary Fig. 3 (continued below)

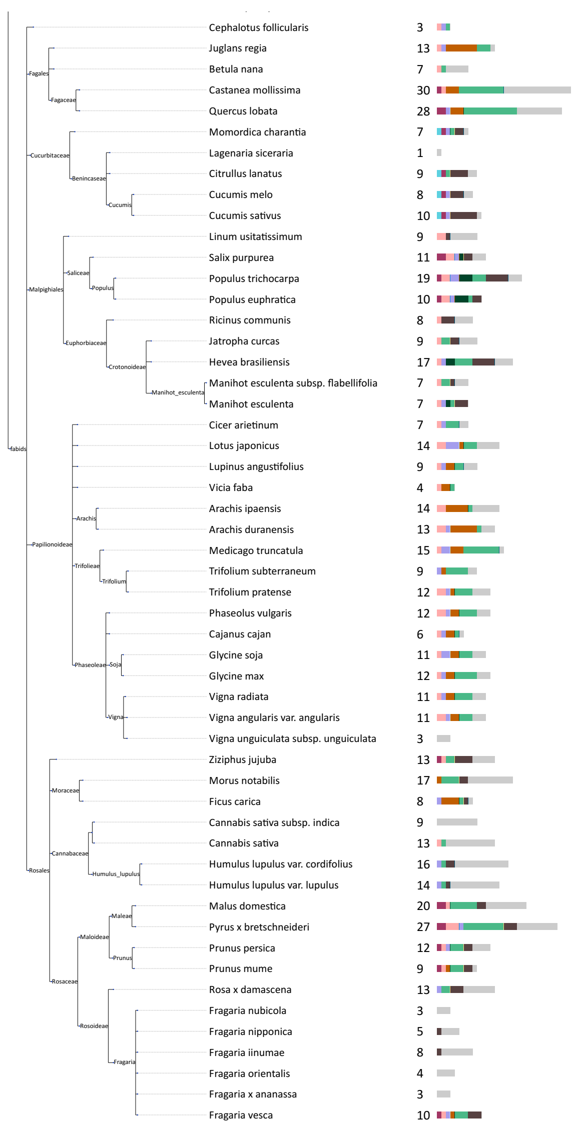

Supplementary Fig. 3 | OSC 'fingerprinting' for species across the Viridiplantae. Relationships are represented according to NCBI taxonomy (www.ncbi.nlm.nih.gov/Taxonomy/Browser). Total number of OSCs are shown, and a bar chart representing the OSC groups is given with colours as in Fig. 2.

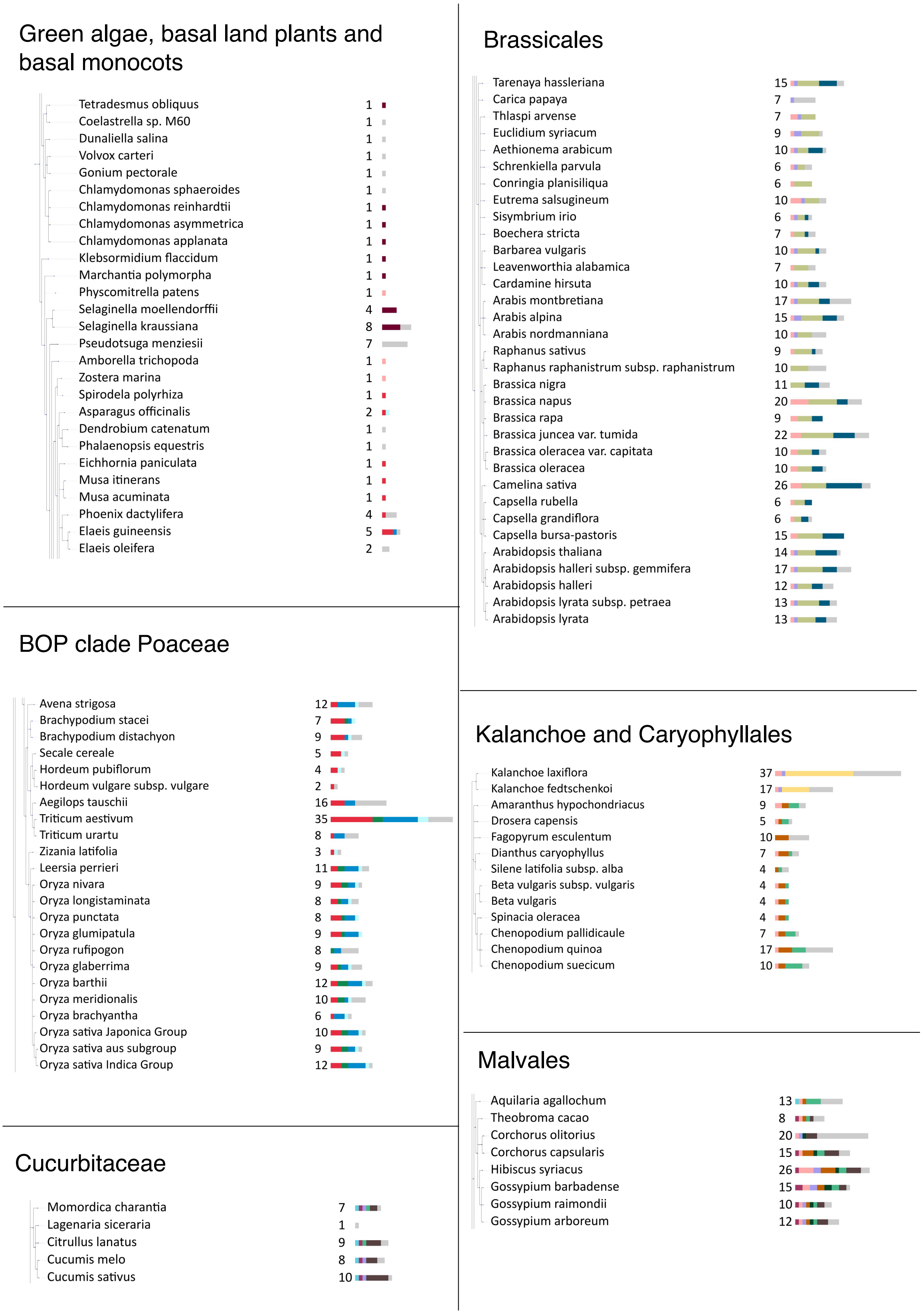

Supplementary Fig. 4 | Examples of OSC 'fingerprinting' in different plant clades. The figure shows how distinctive the OSC complement can be to a given taxonomic group. Cases where there are notably low/poor OSC counts/annotation are likely the result of poorer quality source genome assemblies (e.g. *Lagenaria siceraria*). Colours are as in Fig. 2.

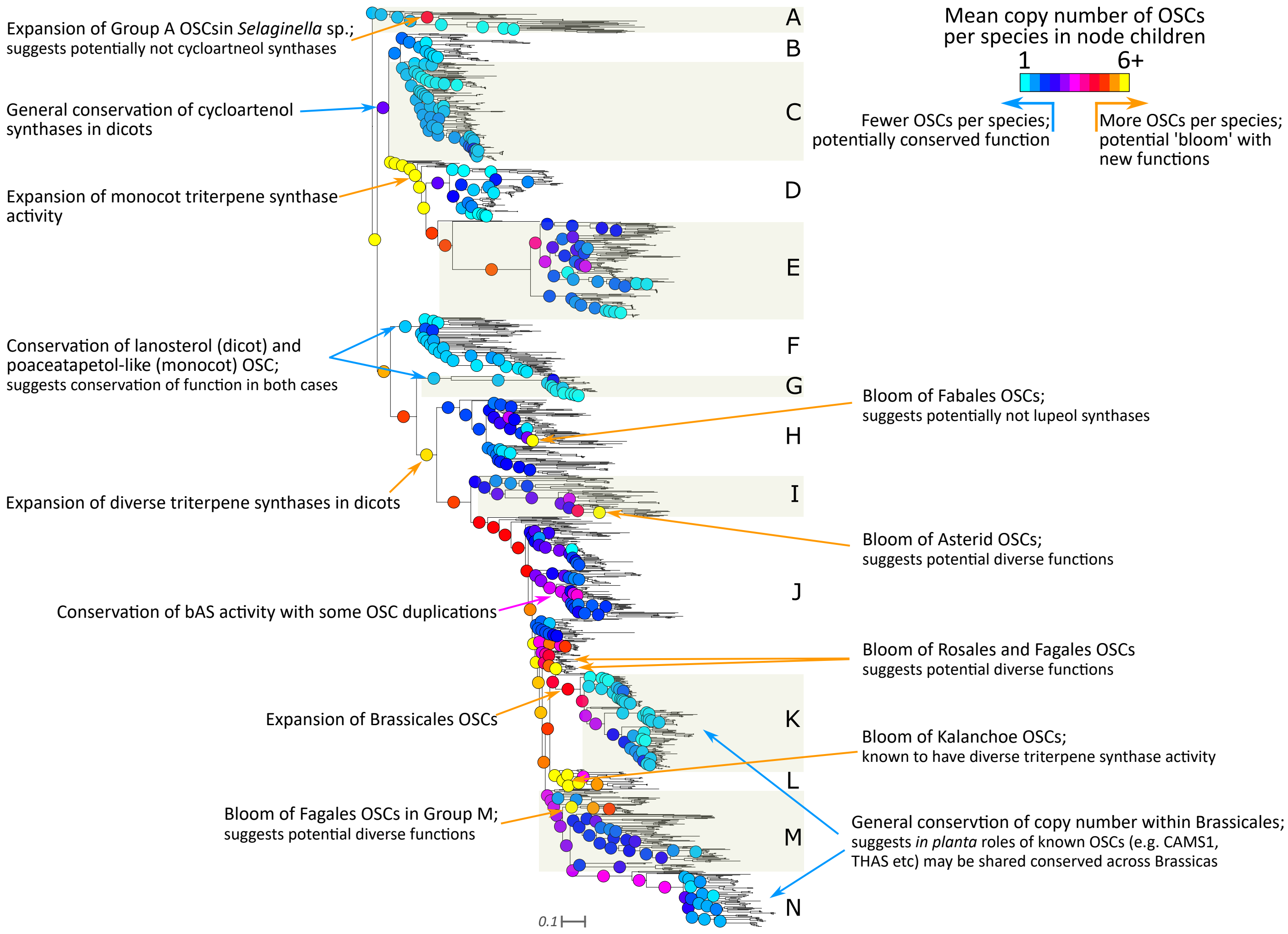

Supplementary Fig. 5 | Phylogenetic tree showing the mean copy number of OSCs per species for the children at each node (for nodes of >8 leaves). Phylogeny displayed as in Fig. 2. Large clades which have a conserved low number of OSCs per species are indicative of those clades with a broadly conserved function (e.g. group F lanosterol synthases). Cases where there are a large number of OSCs per species are indicative of potential diversifications of function. Examples of both of these are labelled, with a mean copy number of approximately four or greater being considered for a 'bloom' event.

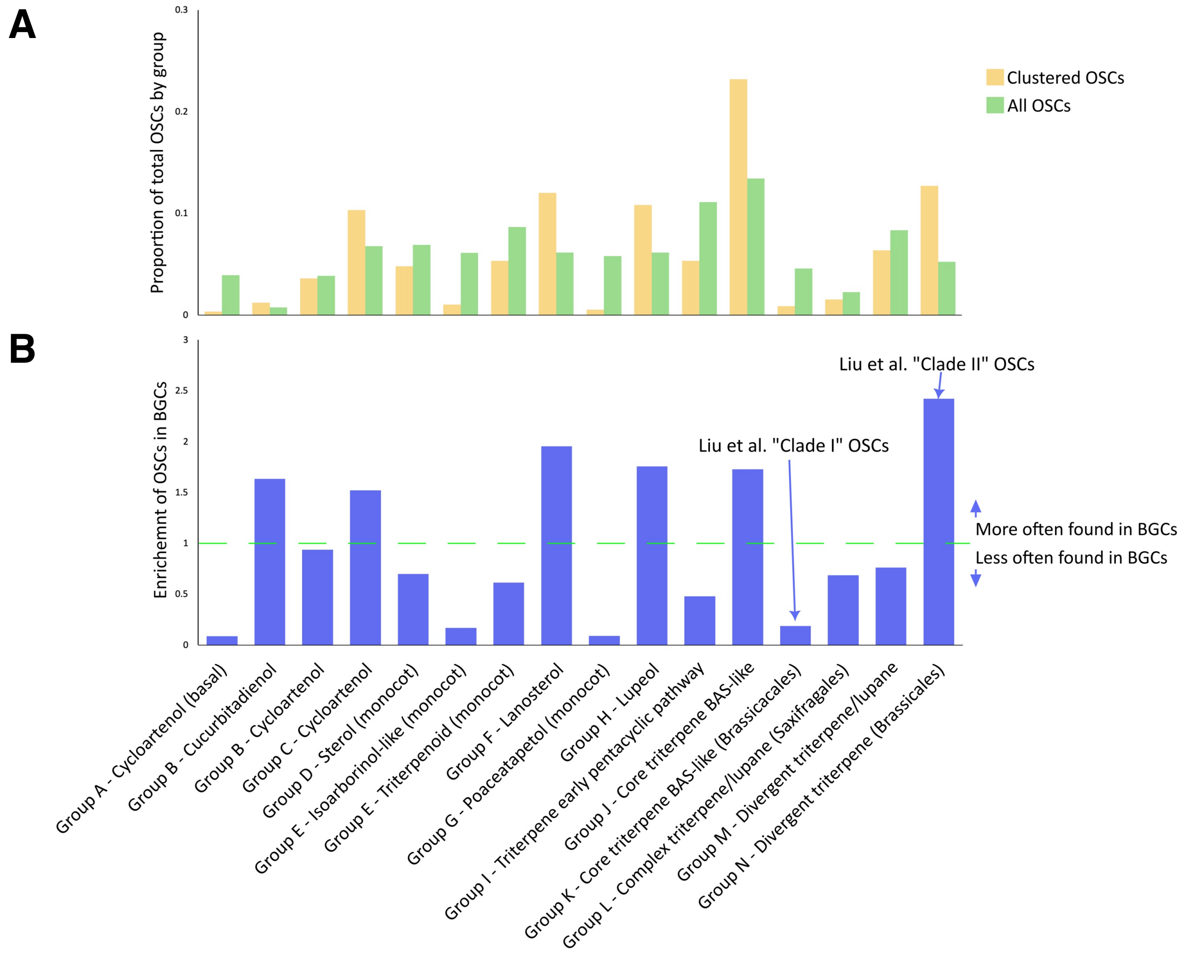

Supplementary Fig. 6 | Enrichment of different OSC groups in biosynthetic gene clusters (BGCs). **A)** Relative proportions of OSCs found in BGCs (by OSC group), and proportion of OSCs (by group) regardless of clustering. For each OSC family, the relative proportion which were present in plantiSMASH-release (https://bitbucket.org/satriaphd/plantismash-release/src/master/) BGCs out of the total set of clustered OSCs was calculated (yellow). The relative proportion by OSC group was calculated for all OSCs regardless of clustering (green). **B)** The ratio of these was then calculated and is presented. A ratio of >1 means the OSC family is enriched in BGCs. Previous work by Liu et al.[1] noted that the two main Brassicales OSC clades were markedly different in their pattern of forming part of gene clusters. This is confirmed in these data, and shows that other OSC groups have similar strong associations or disassociations with gene clustering.

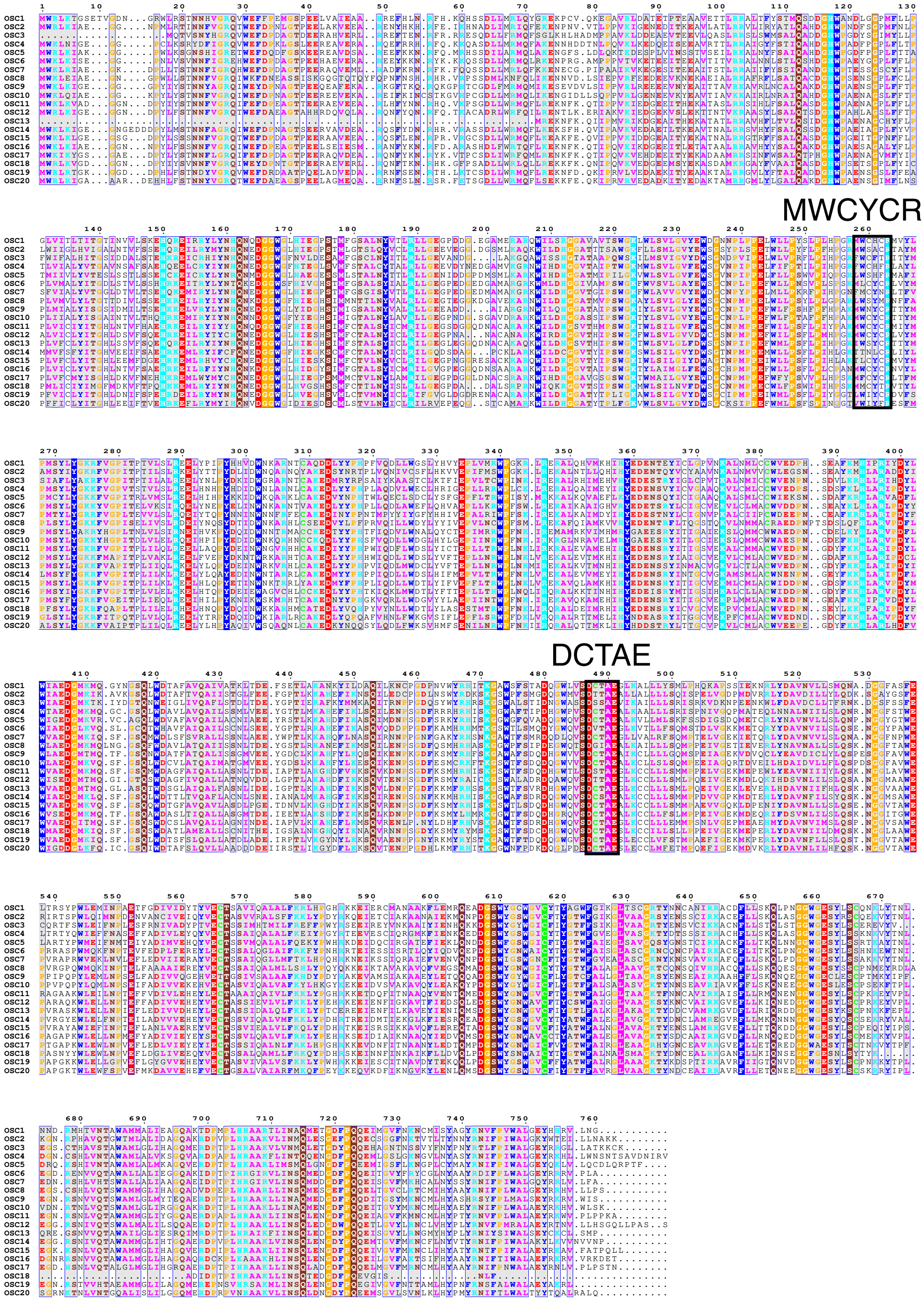

Supplementary Fig. 7 | Alignment of selected OSC sequences. Conserved regions are highlighted. Image generated with ESPript 3.0 (https://espript.ibcp.fr/ESPript). The DCTAE and M(W/L)C(Y/H)CR motifs[2] are shown.

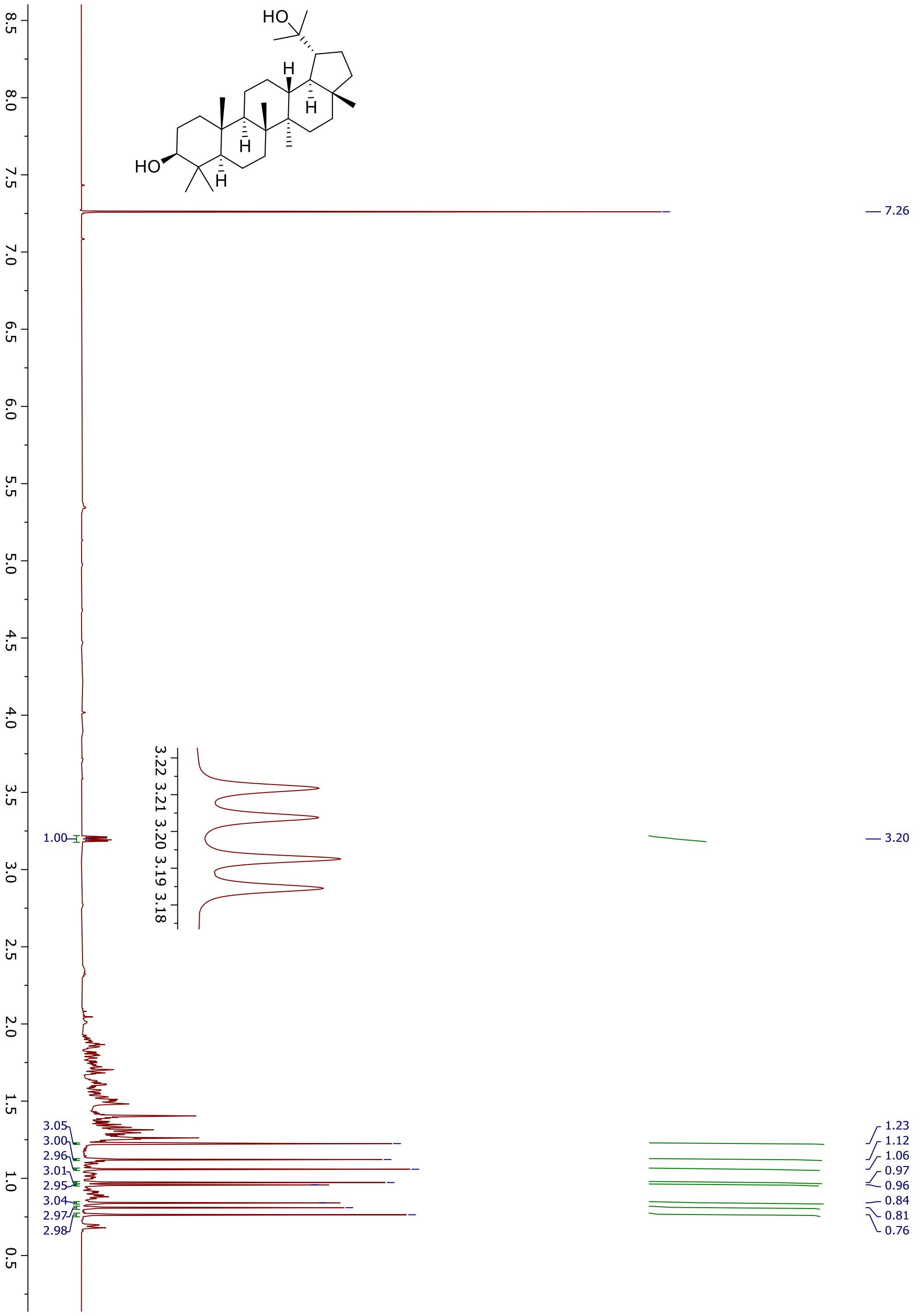

Supplementary Fig. 8 | ^{1}H NMR spectrum of a compound **39** sample isolated from large-scale expression of OSC19 (600 MHz, $CDCl_3$). GC-MS peaks at relative retention times (RRT) 1.68 and 3.57 min were inseparable. The sample shows the presence of just one triterpene which matches the literature for 3β,20-dihydroxylupane. Literature NMR Values[3]: ^{1}H NMR (500 MHz, $CDCl_3$) δ 3.20 (1H, dd, *J* = 11.3, 4.9, H-3), 1.23 (3H, s, H-29/30), 1.12 (3H, s, H-29/30), 1.06 (3H, s, H-26), 0.97 (3H, s, H-23), 0.96 (3H, s, H-27), 0.84 (3H, s, H-25), 0.81 (3H, s, H-28), 0.76 (3H, s, H-24). Compound **39** degrades during GC-MS analysis to give two peaks at relative retention times 1.68 and 3.57 min. The peak at RRT 1.68 matches lupeol (**21**), consistent with partial dehydration of 39 during vaporization. Peak at RRT 3.57, produces EI spectrum similar to lupeol (**21**) consistent with dehydration of surviving 39 during ionization.

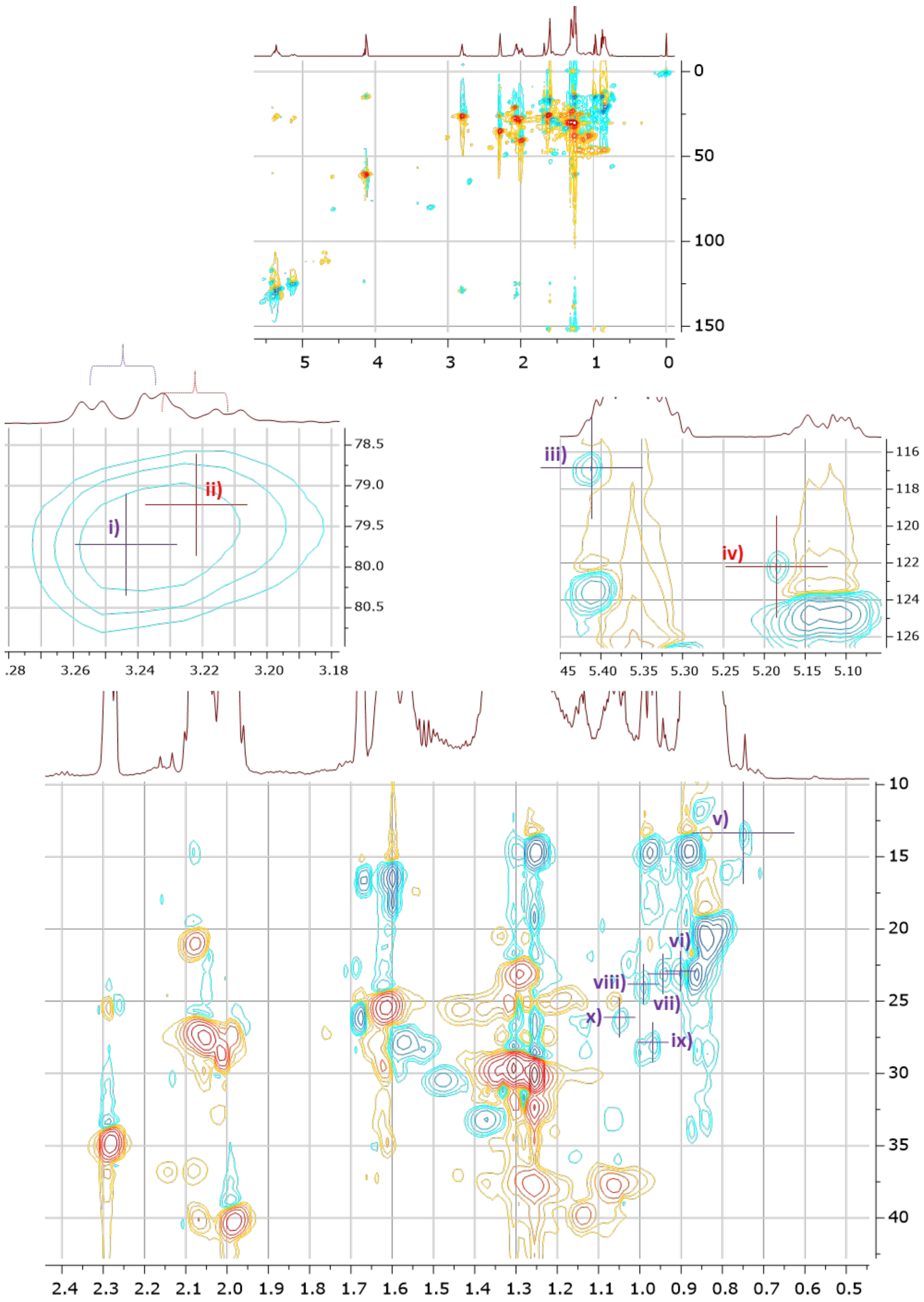

Supplementary Fig. 9 | DEPT-Edited-HSQC spectra confirming presence of baurenol. The EI mass spectrum of (**28**) was consistent with the known triterpene bauerenol (Supplementary Fig. 26). A metabolomic NMR approach was used to confirm the presence of bauerenol (**28**) in the product profile of OSC6. The figure shows sections of a DEPT-Edited-HSQC spectra of an enriched extract from a 6 leaf-scale expression (See Methods). Characteristic correctly phased DEPT-Edited-HSQC cross-peaks corresponding to the ^{1}H and ^{13}C chemical shifts for the alkene of each compound were identified, along with other identifiable signals. DEPT-Edited-HSQC and characteristic NMR signals identified from cross-peaks (600/150 MHz $CDCl_3$): i) bauerenol C3,^{1}Hδ 3.2449, ^{13}Cδ 79.68 (Lit: 3.24, 79.4). ii) β-amyrin C3, ^{1}Hδ 3.2228, ^{13}Cδ 79.29 (Lit: 3.22, 79.0). iii) bauerenol C7 ^{1}Hδ 5.4109, ^{13}Cδ 116.85 (Lit: 5.41, 116.5). iv) β-amyrin C12, ^{1}Hδ 5.1833, ^{13}Cδ 122.17 (Lit: 5.18, 121.7). v) bauerenol C25, ^{1}Hδ 0.7435, ^{13}Cδ 13.32 (Lit: 0.74, 13.1). vi) bauerenol C30,^{1}Hδ 0.9003, ^{13}Cδ 22.96 (Lit: 0.90, 22.7). vii) bauerenol C27,^{1}Hδ 0.9418, ^{13}Cδ 23.05 (Lit: 0.94, 22.8). viii) bauerenol C26,^{1}Hδ 0.9907, ^{13}Cδ 24.00 (Lit: 0.99, 23.8). ix) bauerenol C24, ^{1}Hδ 0.9652, ^{13}Cδ 27.93 (Lit: 0.96, 27.6). x) bauerenol C29, ^{1}Hδ 1.0454 ^{13}Cδ 26.17 (Lit: 1.04, 25.8). Literature values for baurenol.[4] Literature values for β-amyrin.[5]

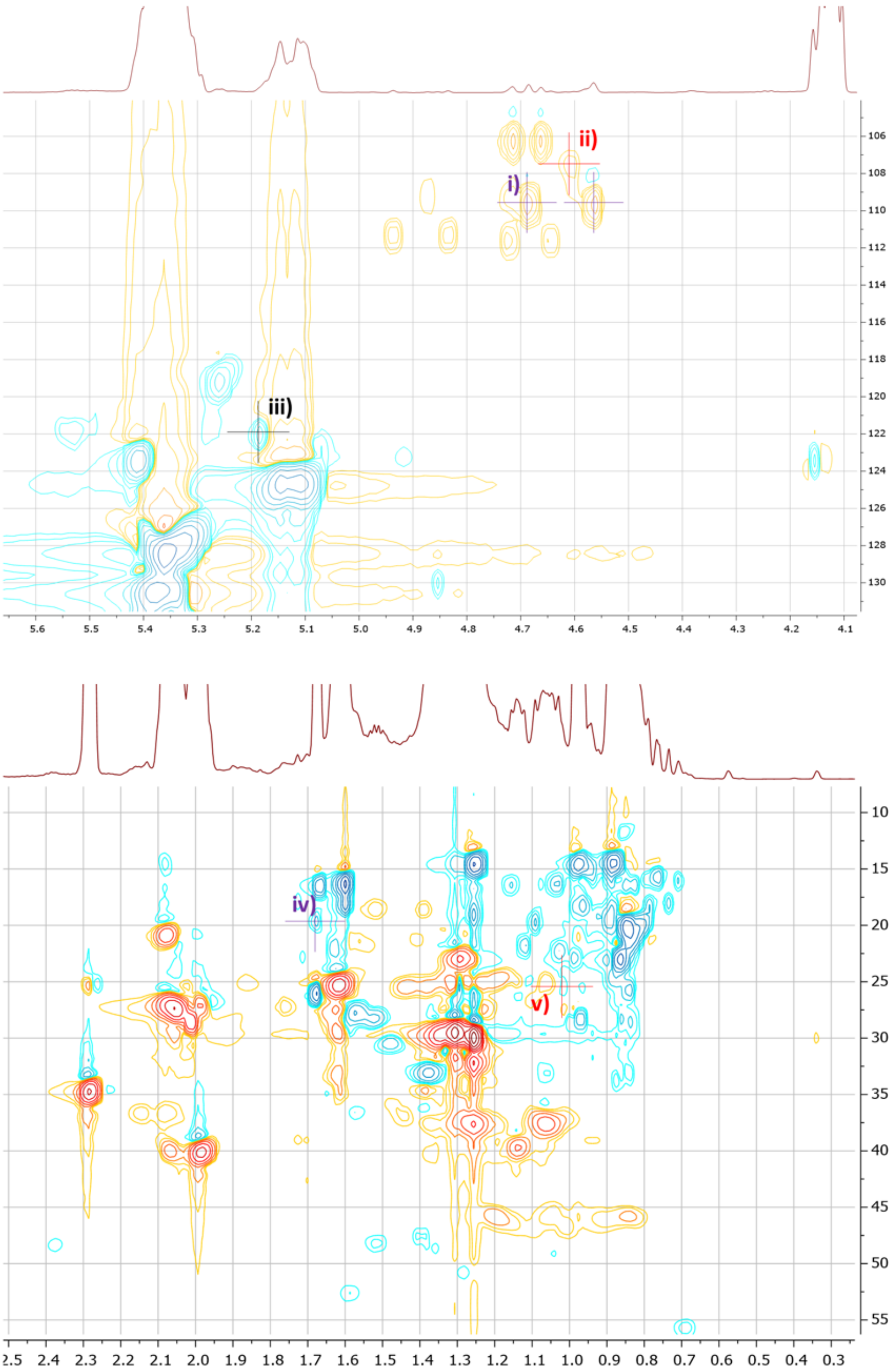

Supplementary Fig. 10 | DEPT-Edited-HSQC spectra confirming presence of taraxasterol. The EI mass spectrum of (**29**) was consistent with the known triterpene taraxasterol (Supplementary Fig. 27). A metabolomic NMR approach was used to confirm the presence of taraxasterol (**29**) in the product profile of OSC7. The figure shows sections of a DEPT-Edited-HSQC spectra of an enriched extract from a 6 leaf-scale expression (See methods). Characteristic correctly-phased DEPT-Edited-HSQC cross-peaks corresponding to the ^{1}H and ^{13}C chemical shifts for the alkene of each compound were identified, along with other identifiable signals. DEPT-Edited-HSQC and characteristic NMR signals identified from cross-peaks (600/150 MHz $CDCl_3$): i) Lupeol, C29, ^{1}Hδ 4.6863, 4.5617, ^{13}Cδ 109.67 (Lit: 4.69, 4.57, 109.3). ii) taraxasterol C30, ^{1}Hδ 4.6080, ^{13}Cδ 107.41 (Lit: 4.61, 107.15). iii) β-amyrin, C12, ^{1}Hδ 5.1840, ^{13}Cδ 122.04 (Lit: 5.18, 121.7). iv) Lupeol, C30, ^{1}Hδ 1.6804, ^{13}Cδ 19.64 (Lit: 1.69, 19.3). v) taraxasterol C29, ^{1}Hδ 1.0157, ^{13}Cδ 25.56 (Lit: 1.02, 25.54). Literature values for lupeol and taraxasterol.[6] Literature values for β-amyrin.[5]

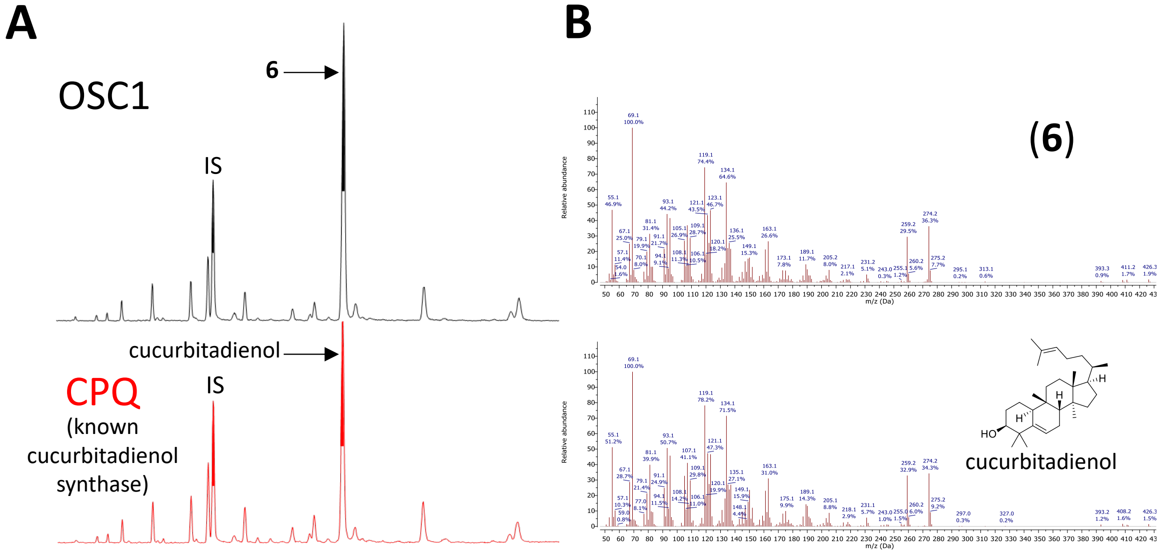

Supplementary Fig. 11 | GC-MS comparison of OSC1 product profile and that of expression of known cucurbitadienol synthase (CPQ) from *Cucumis sativus* (Gene: Csa6G088690, known as CsBi).[7] Main product matches retention time and EI spectrum of that of CPQ known to be cucurbitadienol (**6**). **A)** Total Ion Chromatogram, **B)** EI Spectrum. IS = internal standard, coprostanol.

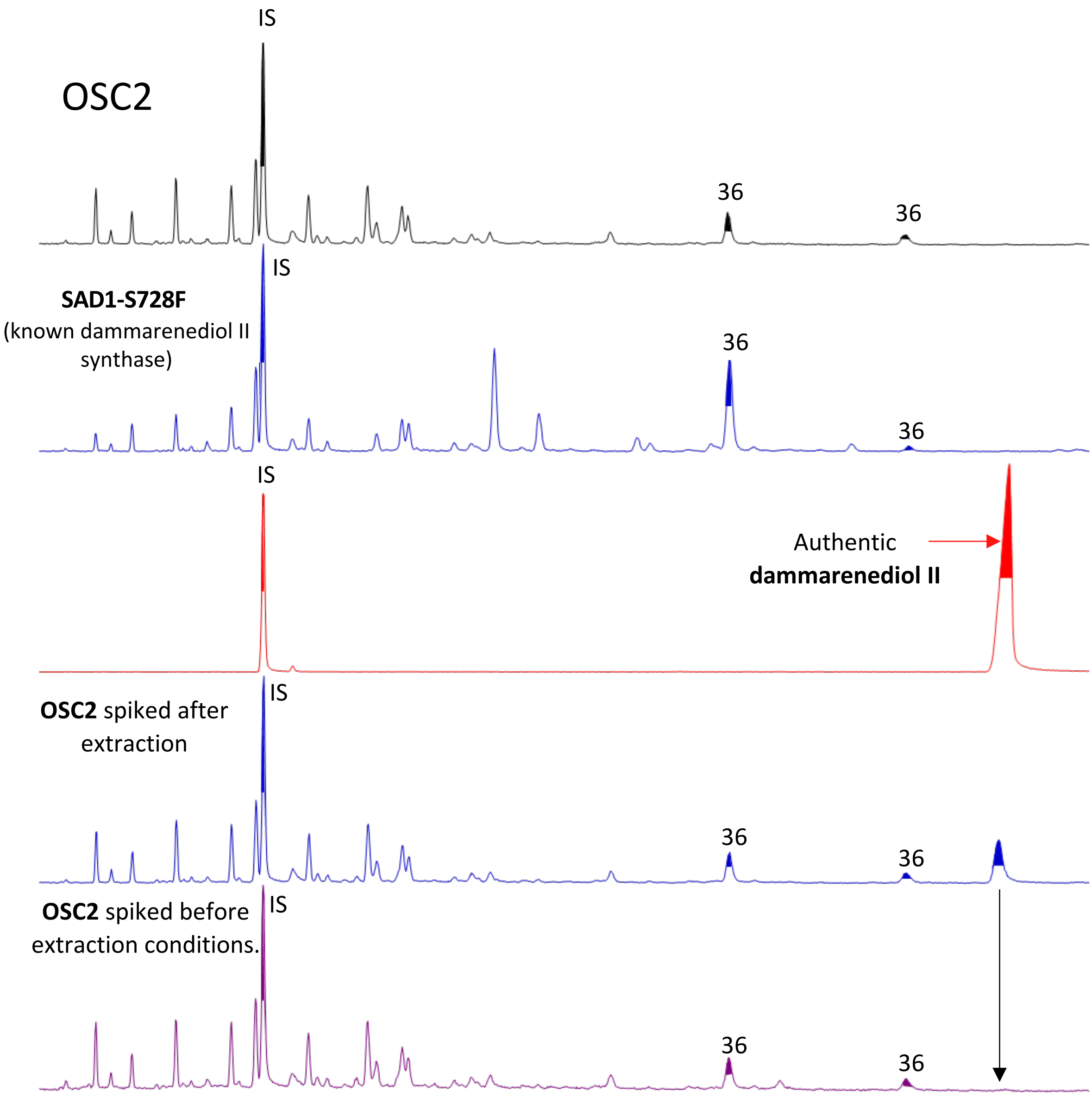

Supplementary Fig. 12 | GC-MS comparison of OSC2 product profile and that of expression of known dammarenediol II synthase (SAD1-S728F)[8]. The two peaks for (**36**) at RRT 2.82 min and 3.89 min are degradation artifacts of the extraction method, which are also seen with expression SAD-S728F. When spiked after extraction with a commercial standard of dammarenediol II (from ChemFaces), dammarenediol II is visible in the chromatogram. However, if spiked before extraction, the spiked dammarenediol II degrades during extraction and is not visible in the chromatogram.

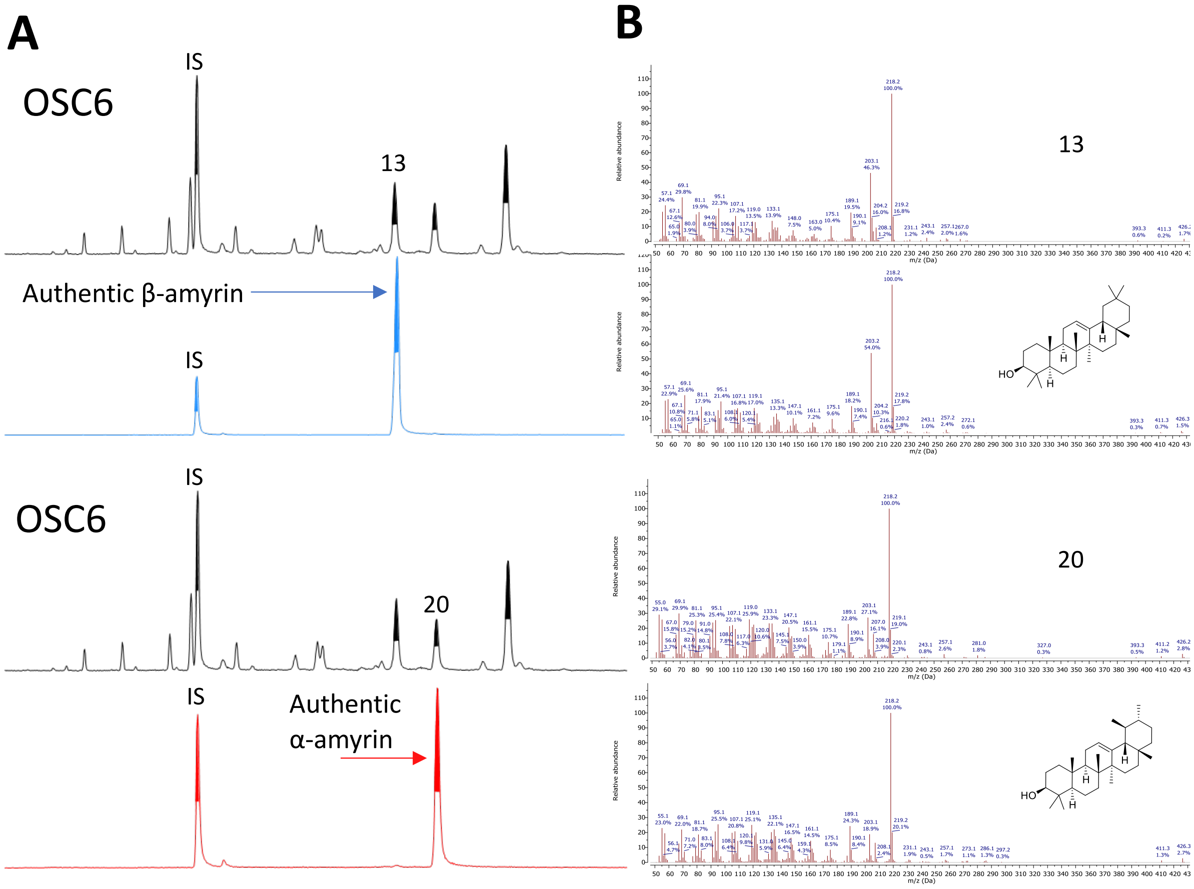

Supplementary Fig. 13 | GC-MS comparison of OSC6 product profile with authentic standards of β-amyrin (from Extrasynthese) and α-amyrin (Extrasynthese). **A)** Total Ion Chromatogram, **B)** EI Spectrum. IS = internal standard, coprostanol.

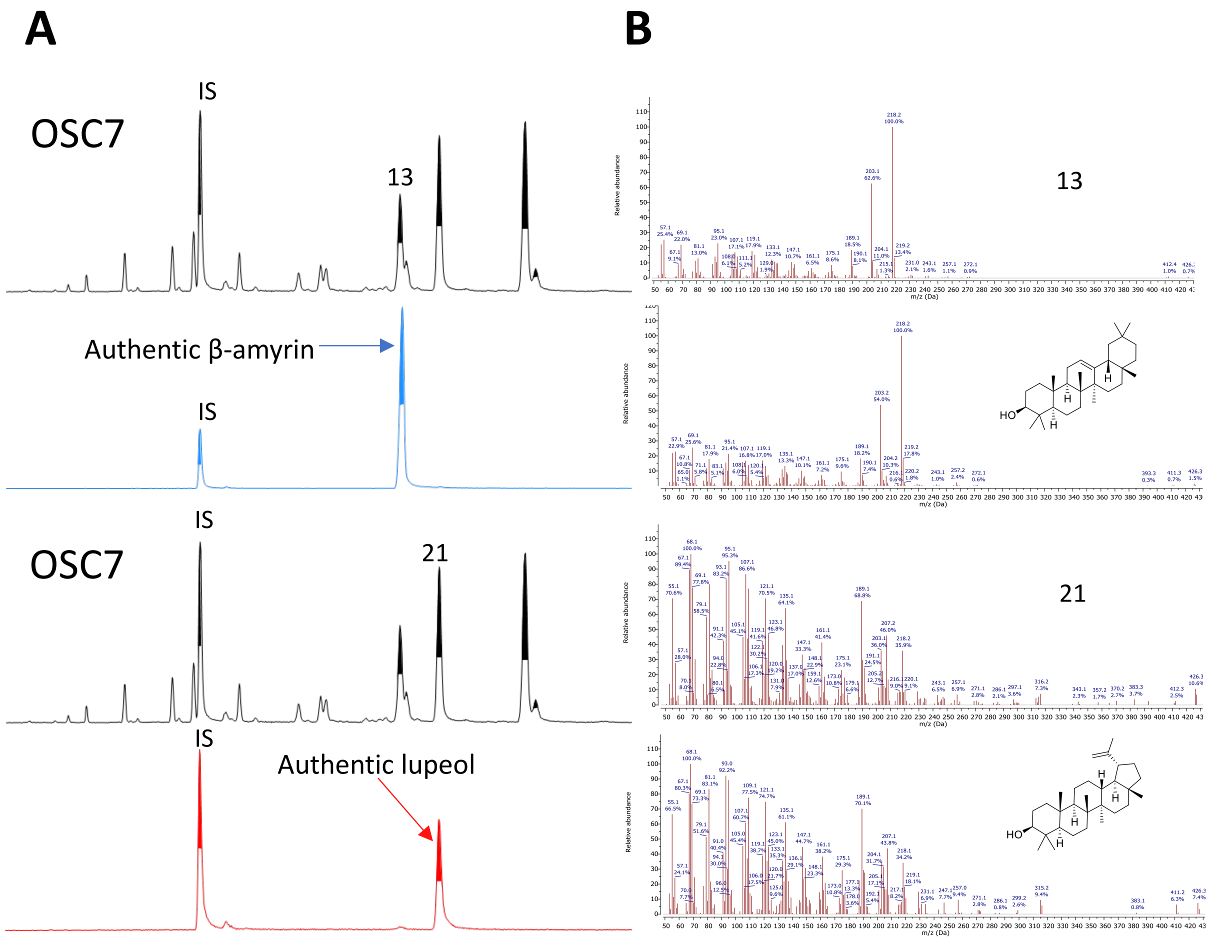

Supplementary Fig. 14 | GC-MS comparison of OSC7 product profile with authentic standards of β-amyrin (Extrasynthese) and lupeol (Extrasynthese). **A)** Total Ion Chromatogram, **B**) EI Spectrum. IS = internal standard, coprostanol.

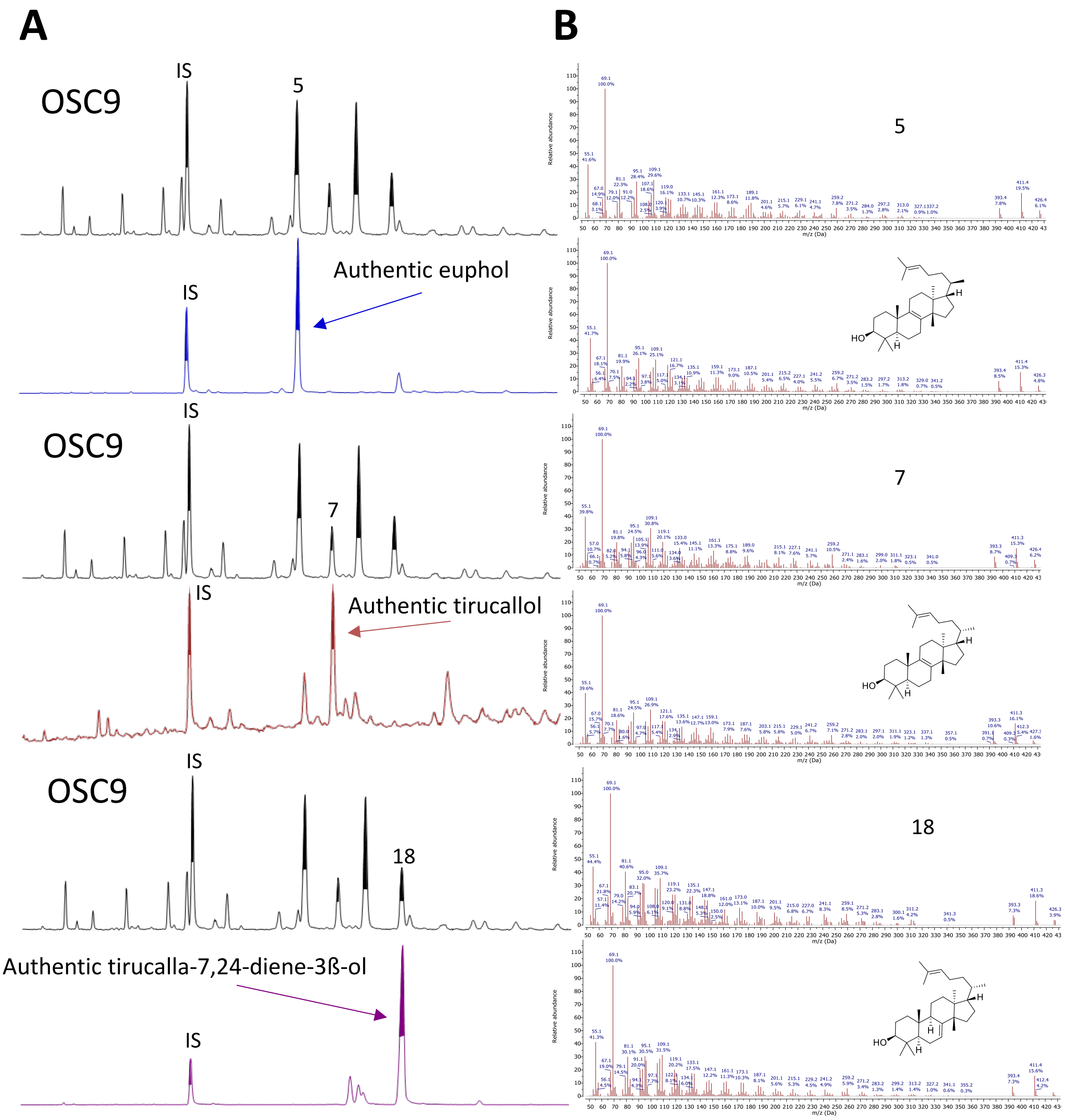

Supplementary Fig. 15 | GC-MS comparison of OSC9 product profile with authentic standards of euphol (Extrasynthese), tirucalla-7,24-diene-3β-ol (Hodgson et al)[9], and a semi-crude sample of tirucallol (Liu et al)[1]. **A)** Total Ion Chromatogram, **B**) EI Spectrum. IS = internal standard, coprostanol.

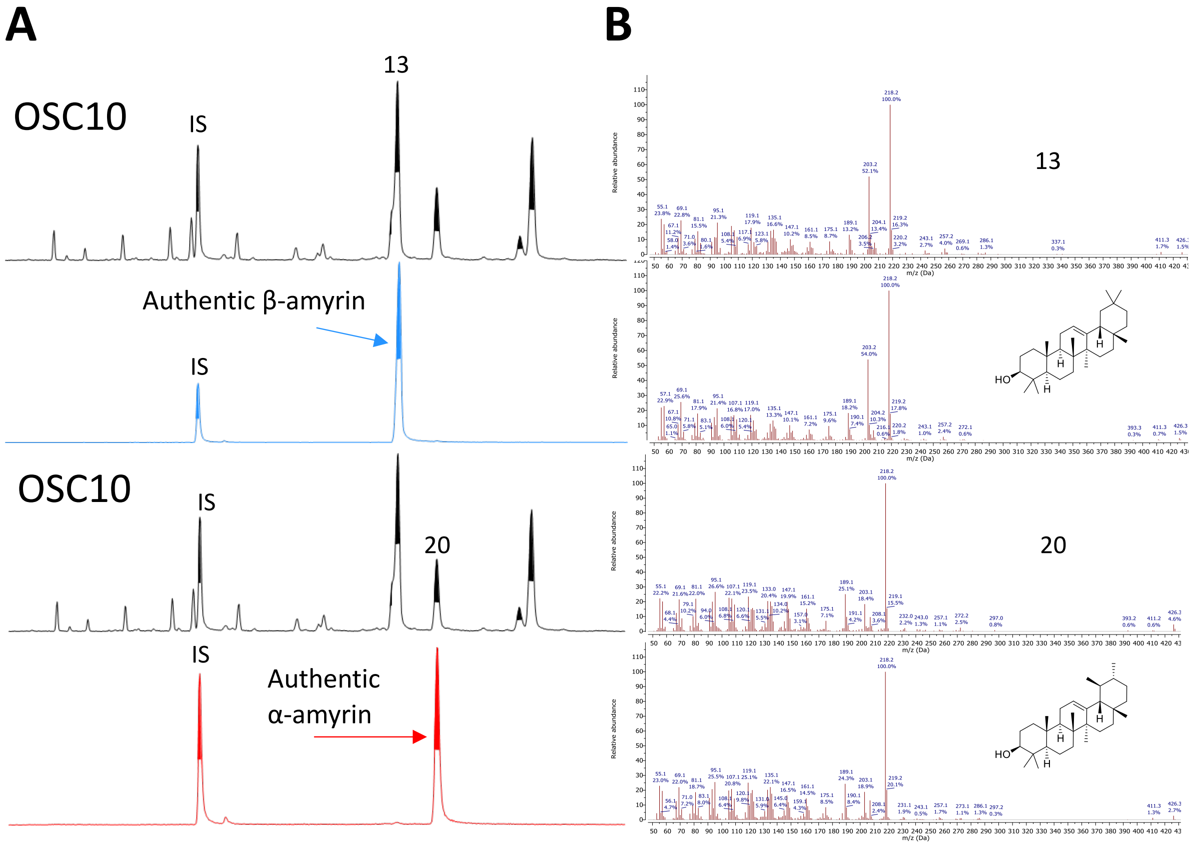

Supplementary Fig. 16 | GC-MS comparison of OSC10 product profile with authentic standards of β-amyrin (Extrasynthese) and α-amyrin (Extrasynthese). **A)** Total Ion Chromatogram, **B)** EI Spectrum. IS = internal standard, coprostanol.

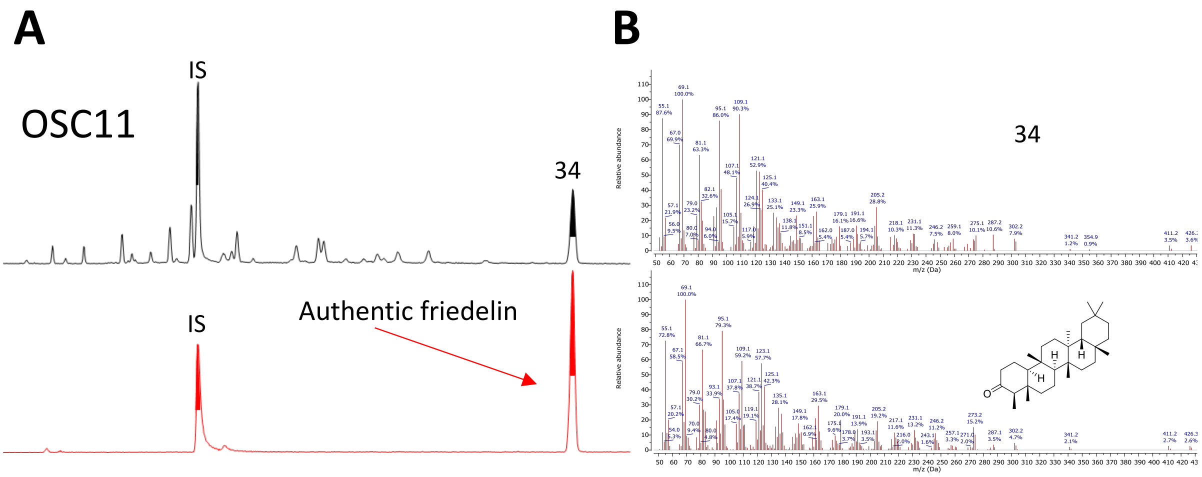

Supplementary Fig. 17 | GC-MS comparison of OSC11 product profile with an authentic standard of friedelin (Extrasynthese). **A)** Total Ion Chromatogram, **B)** EI Spectrum. IS = internal standard, coprostanol.

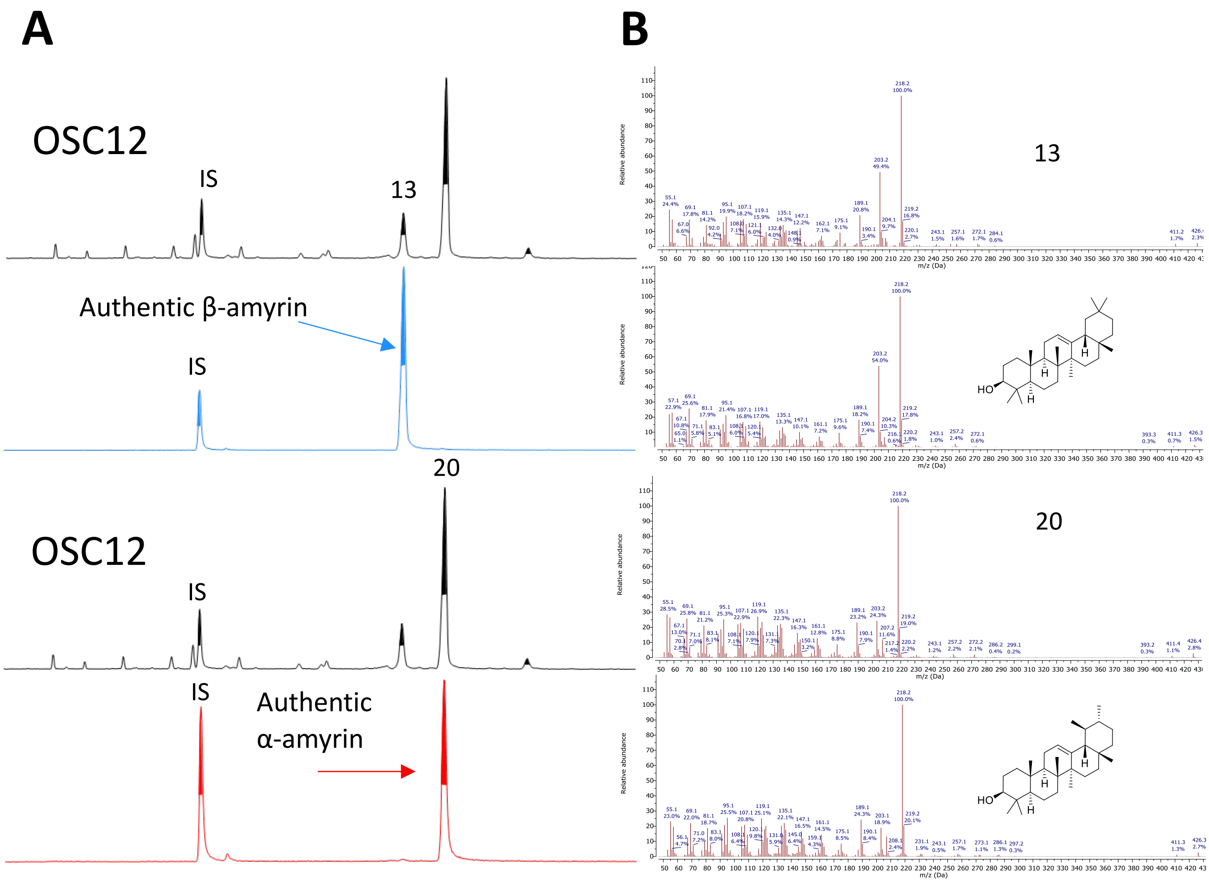

Supplementary Fig. 18 | GC-MS comparison of OSC12 product profile with authentic standards of β-amyrin (Extrasynthese) and α-amyrin (Extrasynthese). **A)** Total Ion Chromatogram, **B)** EI Spectrum. IS = internal standard, coprostanol.

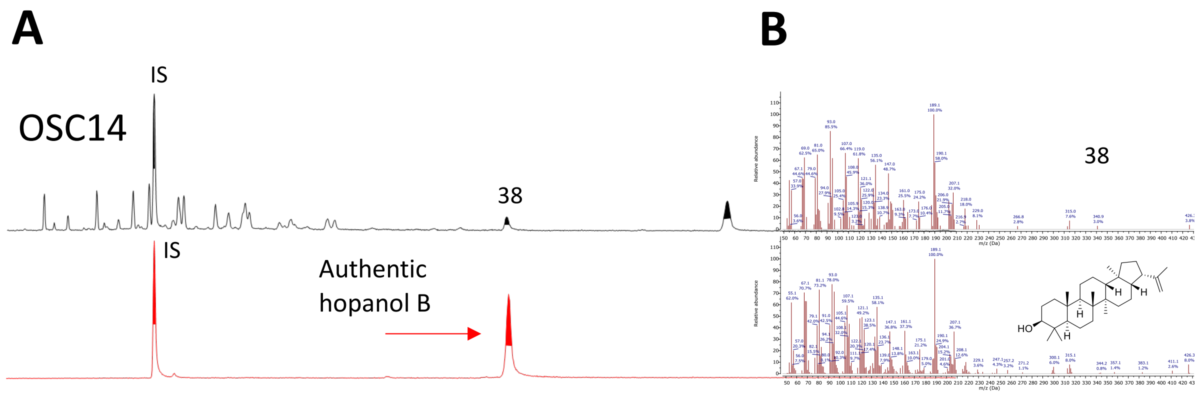

Supplementary Fig. 19 | GC-MS comparison of OSC14 product profile with an authentic standard of hopanol B. **A)** Total Ion Chromatogram, **B)** EI Spectrum. IS = internal standard, coprostanol.

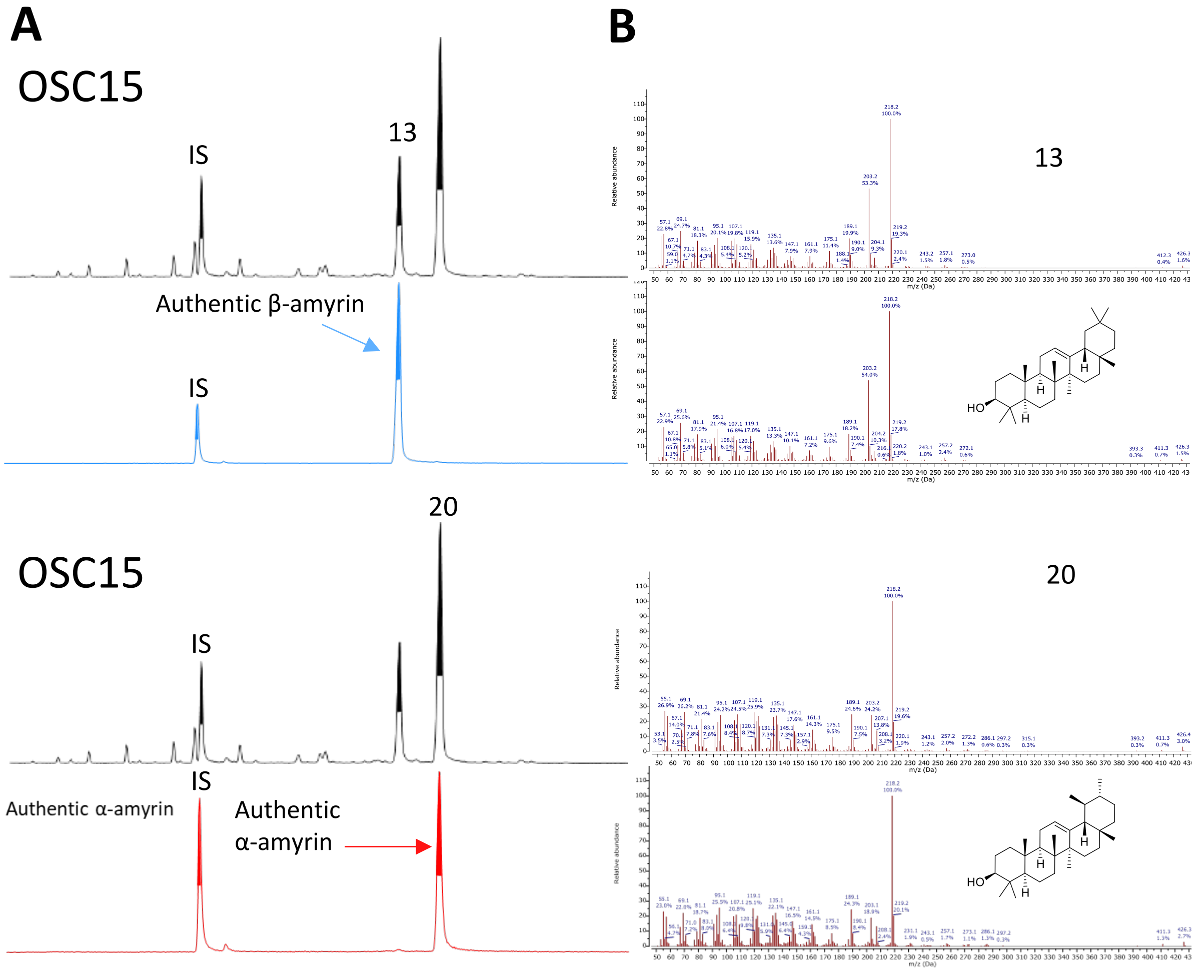

Supplementary Fig. 20 | GC-MS comparison of OSC15 product profile with authentic standards of β-amyrin (Extrasynthese) and α-amyrin (Extrasynthese). **A)** Total Ion Chromatogram, **B)** EI Spectrum. IS = internal standard, coprostanol.

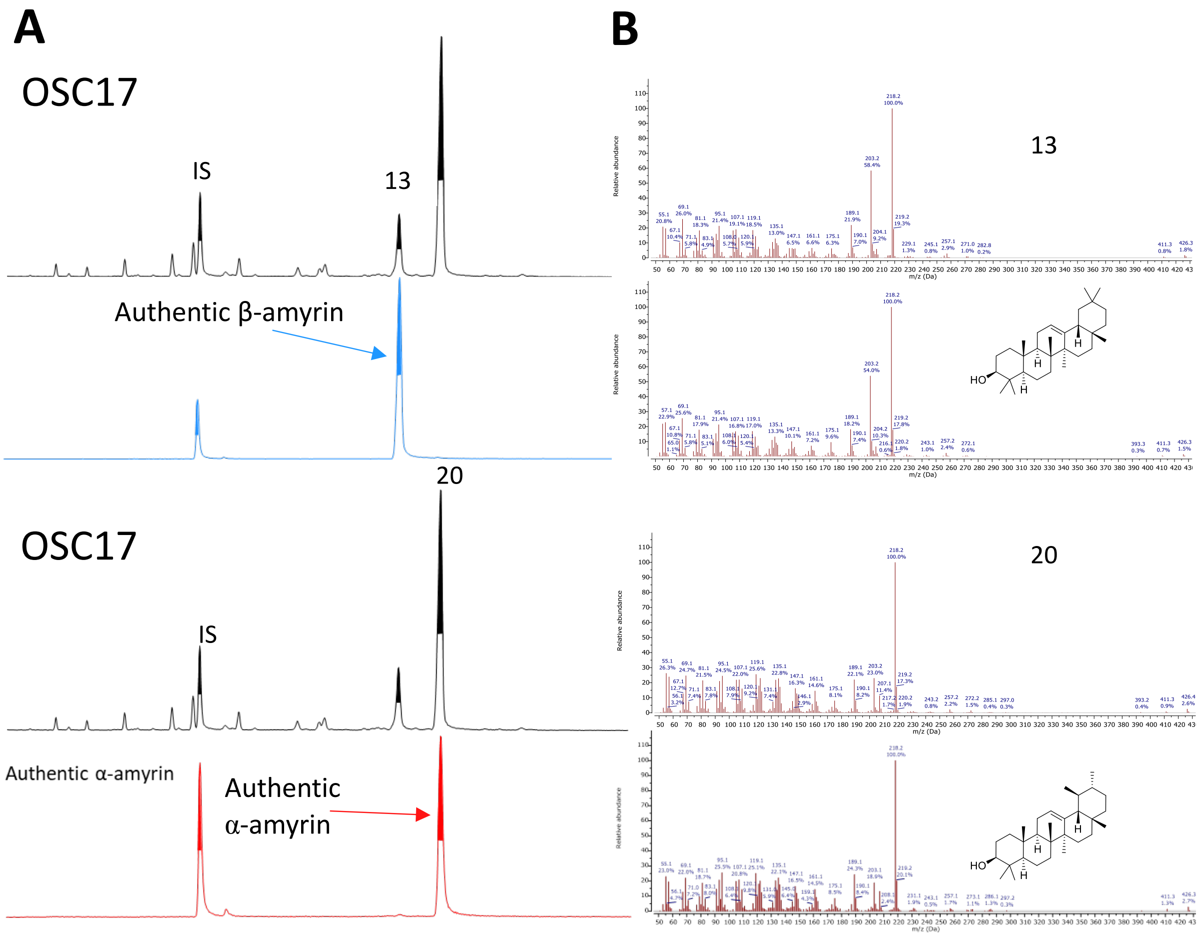

Supplementary Fig. 21 | GC-MS comparison of OSC17 product profile with authentic standards of β-amyrin (Extrasynthese) and α-amyrin (Extrasynthese). **A)** Total Ion Chromatogram, **B)** EI Spectrum. IS = internal standard, coprostanol.

A

1 = RRT -0.14 min

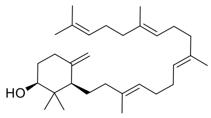

achilleol A-like

EI spectrum of Compound (1) from analysis of OSC8

B

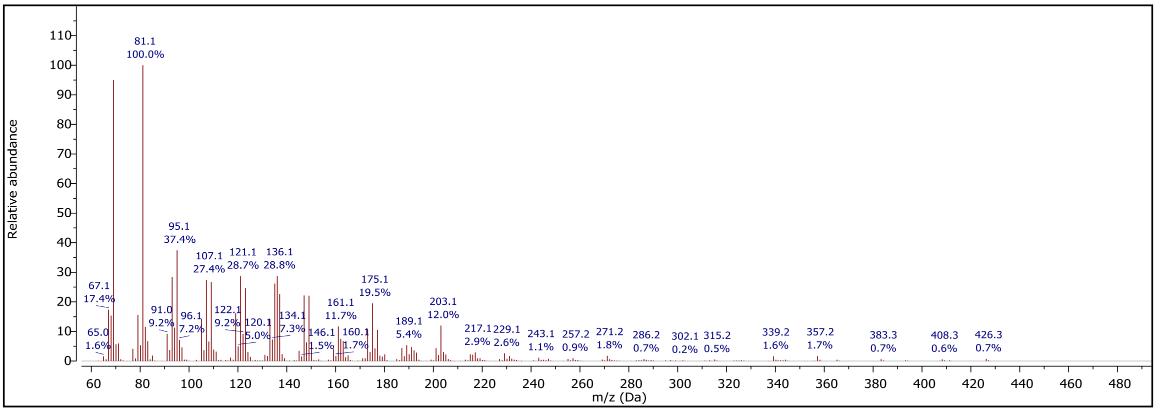

Literature EI spectrum of achilleol A

C

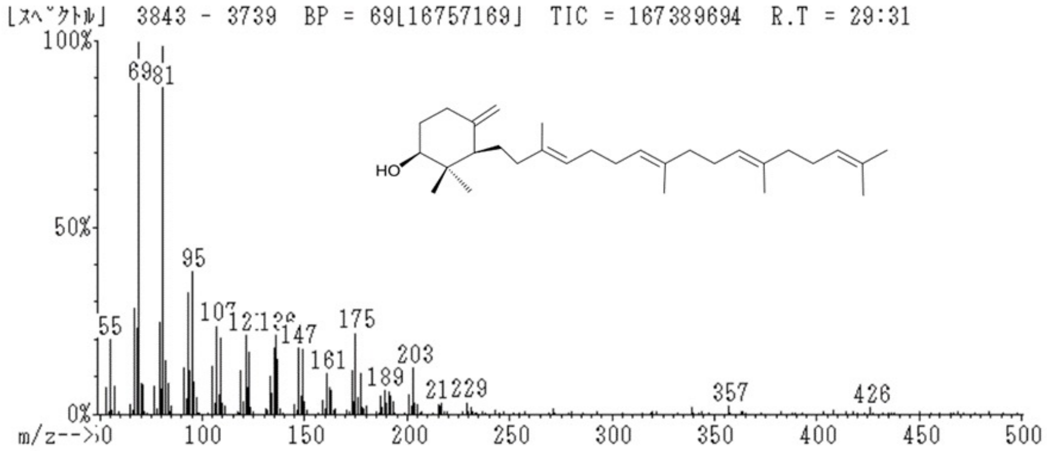

Supplementary Fig. 22 | Putative assignment of (**1**). **A)** Compound (**1**) [RRT -0.14 min] was putatively assigned as achilleol A-like. RRT = Relative Retention Time (Relative to internal standard, coprostanol). Compound (1) has a short retention time and EI fragmentation pattern consistent with a monocyclic triterpene. **B)** The EI spectrum displays a fragmentation pattern similar to that previously reported for achilleol A. Base peaks at 81 and 69 are particularly characteristic. **C)** Literature spectrum of achilleol A (found as compound 12 in supporting information of the reference). [10]

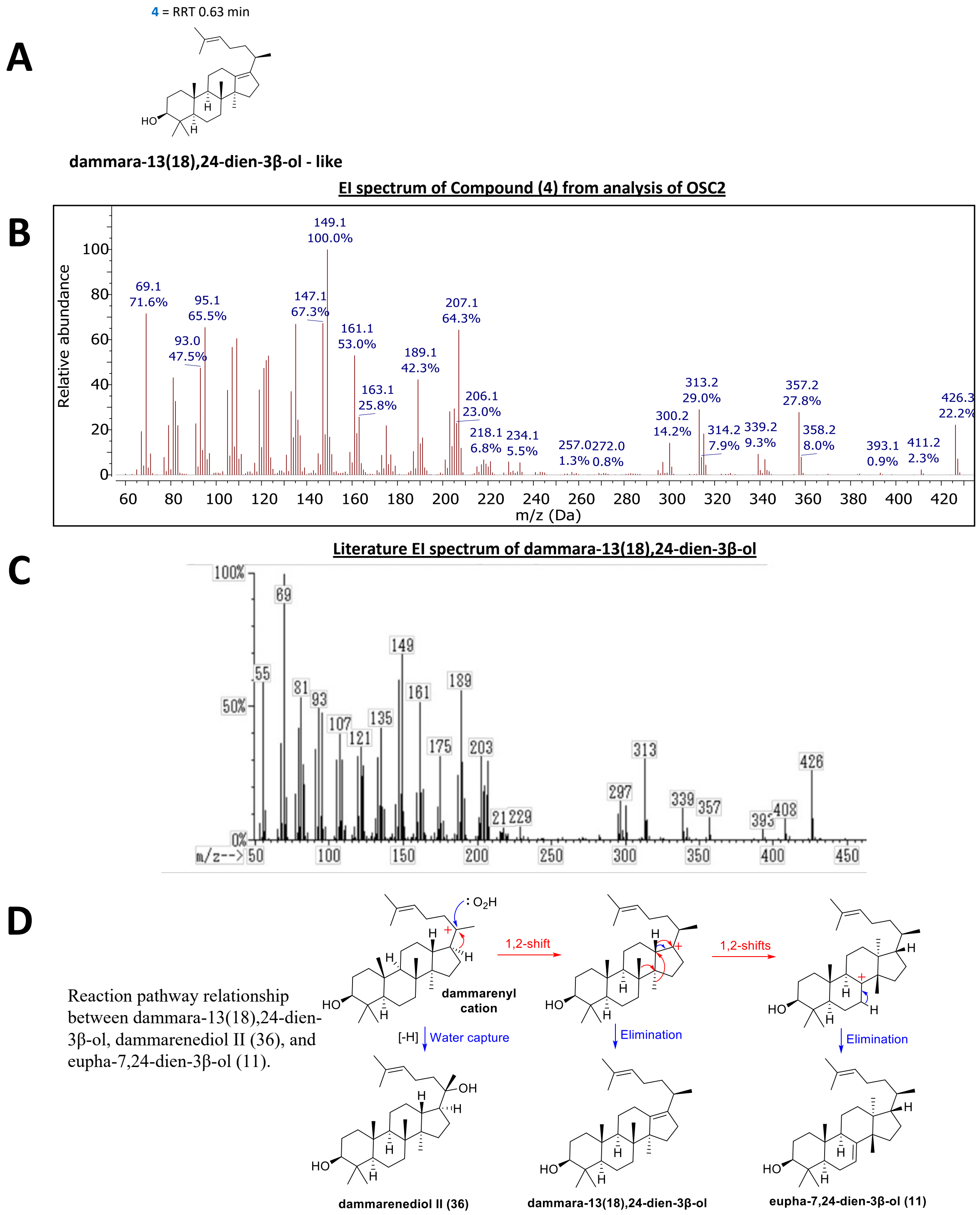

Supplementary Fig. 23 | Putative assignment of (**4**). **A)** Compound (**4**) [RRT 0.63 min] was putatively assigned as dammara-13(18),24-dien-3β-ol – like. RRT = Relative Retention Time (Relative to internal standard, coprostanol). Compound (**4**) is produced by OSC2 which also produces the tetracyclic triterpenes dammarenediol II (**36**), and eupha-7,24-dien-3β-ol (**11**). Compound (**4**) is rationalisable as an elimination product from share intermediatory cations in the reaction pathway between dammarenediol II (**36**) and eupha-7,24-dien-3β-ol (**11**) (see **D**). **B)** The EI spectrum of compound (**4**) displays a fragmentation pattern similar to that previously reported for dammara-13(18),24-dien-3β-ol. **C)** Literature spectrum of dammara-13(18),24-dien-3β-ol.[11] **D)** The reaction pathway relationship between dammara-13(18),24-dien-3β-ol, dammarenediol II (**36**), and eupha-7,24-dien-3β-ol (**11**).

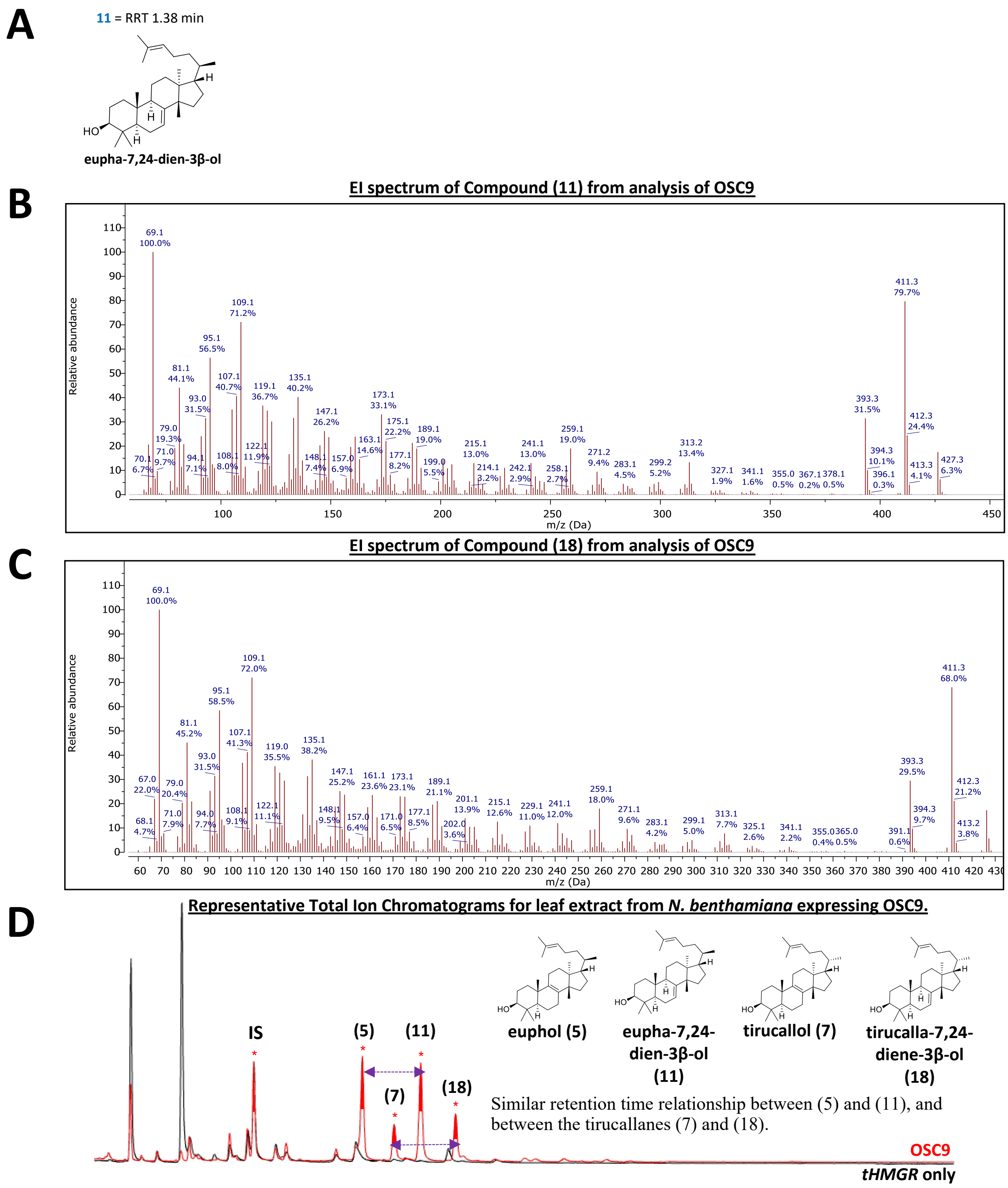

Supplementary Fig. 24 | Putative assignment of (**11**). **A)** Compound (**11**) [RRT 1.38 min] was putatively assigned as eupha-7,24-dien-3β-ol. RRT = Relative Retention Time (Relative to internal standard, coprostanol). Compound (**11**) is produced by OSC9, which also produces the closely related tetracyclic isomers euphol (**5**), tirucallol (**7**), and tirucalla-7,24-diene-3β-ol (**18**). **B)** Compound (**11**) has an identical EI Spectrum to **C)** tirucalla-7,24-diene-3β-ol (**18**) (representative EI spectrums from this work). Furthermore, it has a similar difference in retention time relationship to euphol (**5**), as tirucalla-7,24-diene-3β-ol (**18**) has to its respective isomer tirucallol (**7**) (See **D**). Tirucalla-7,24-diene-3β-ol is the Δ7 isomer of tirucallol. Thus, compound (**11**) was strongly inferred to be the equivalent Δ7 isomer of euphol (eupha-7,24-dien-3β-ol). **D)** Annotated representative Total Ion Chromatograms for leaf extract from N. benthamiana expressing OSC9 (Red). Black, control (tHMGR only). IS, internal standard (coprostanol, 100 ppm).

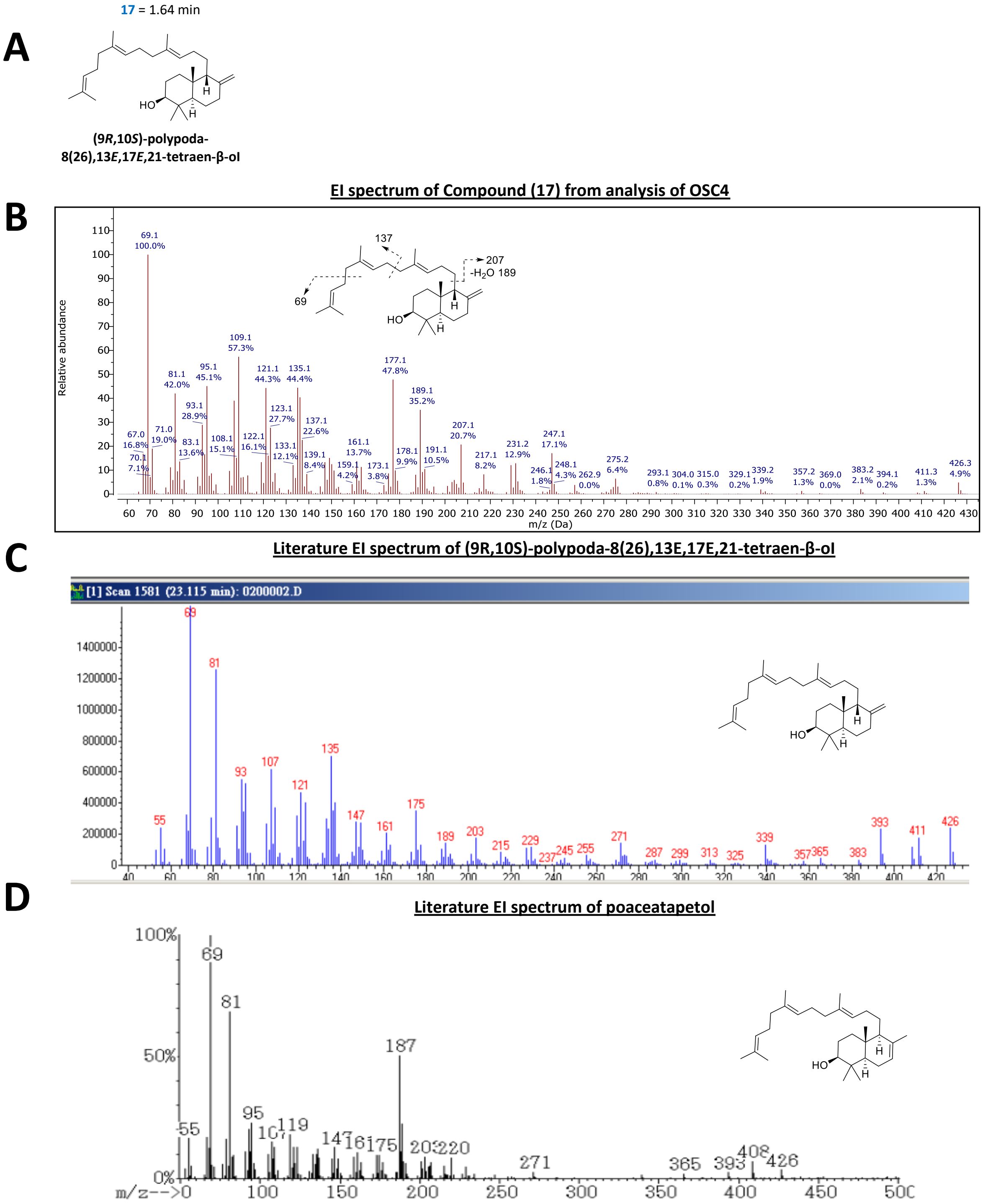

Supplementary Fig. 25 | Putative assignment of (**17**). **A)** Compound (**17**) [RRT 1.64 min] was putatively assigned as a bicyclic triterpene. RRT = Relative Retention Time (Relative to internal standard, coprostanol). **B)** EI spectrum of compound (**17**) (representative EI spectrum from this work). Base peak of 69 in the and a fragment at 137 suggested a partially cyclised triterpene and fragments 189 and 207 were consistent with sidechain cleavage of a bicyclic triterpene. The retention time was also greater then would be expected for a monocyclic triterpene. **C)** Literature spectrum of (9R,10S)-polypoda-8(26),13E,17E,21-tetraen-β-ol (Found in the supporting information of the reference)[12]. **D)** Literature spectrum of poaceatapetol (found in the supporting information of the reference)[10]. The EI spectrum from OSC4 was found to be similar to that reported for (9R,10S)-polypoda-8(26),13E,17E,21-tetraen-β-ol, and this was a closer match to that reported for related isomer poaceatapetol.

A

28 = RRT 2.18 min

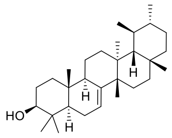

bauerenol

B

EI spectrum of Compound (28) from analysis of OSC6

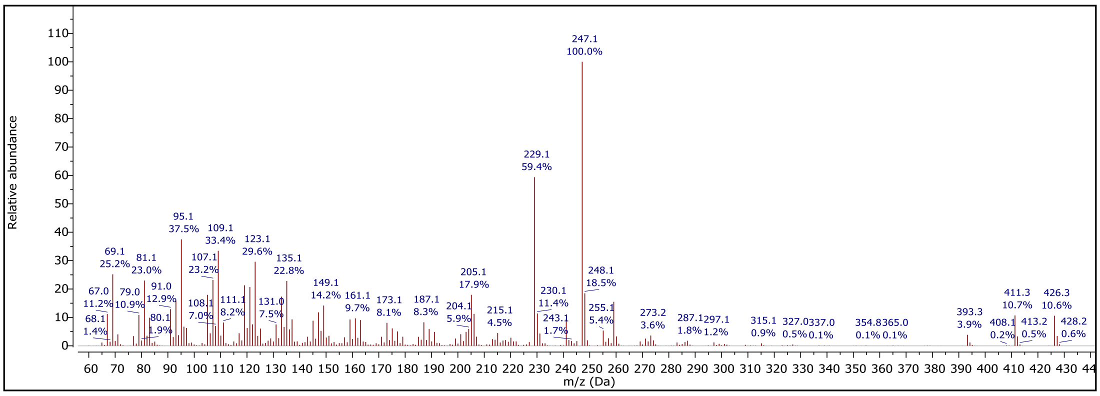

Spectrum of (28) is consistent with the EI spectrum of bauerenol as reported in the literature: EI, m/z (relative intensity): 426 (3), 411 (21), 393 (6), 273 (3), 259 (18), 247 (100), 229 (47), and 205 (18).

Supplementary Fig. 26 | A) Compound (**28**) was identified as baurenol. RRT = Relative Retention Time (Relative to internal standard, coprostanol). **B)** EI mass spectrum of bauerenol (**28**) from this work. The EI spectrum of (**28**) is consistent with that reported in the literature for bauerenol[13].

A

31 = RRT 2.35 min

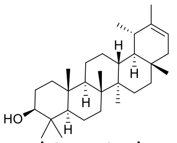

ψ-taraxasterol

B

EI spectrum of Compound (31) from analysis of OSC10

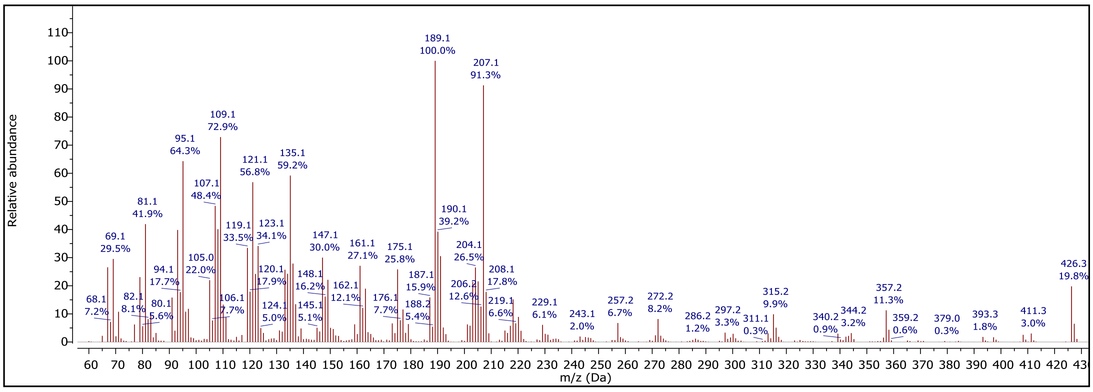

EI spectrum of Compound (29) from analysis of OSC7

C

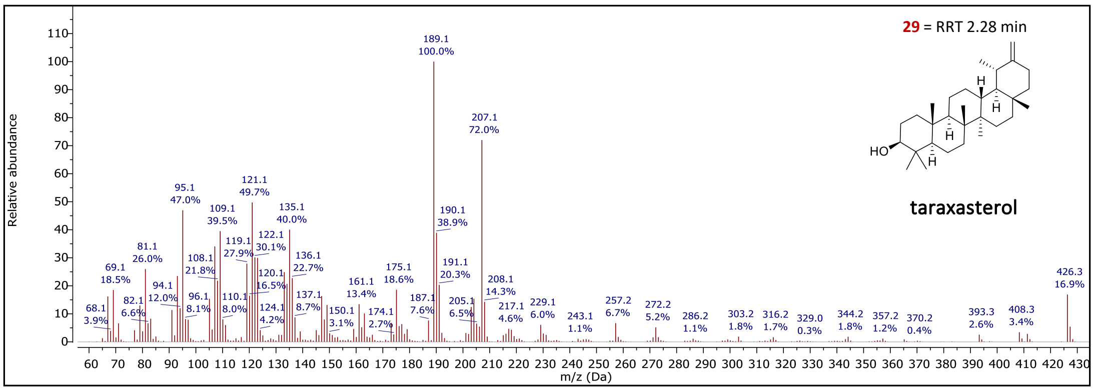

Spectrum of (29) is also consistent with the EI spectrum of taraxasterol as reported in the literature: EI, m/z (relative intensity): 426 (14), 207 (57), 189 (100), 135 (51), 121 (74), 109 (57), 107 (62), 95 (70), 93 (47), 81 (48), 67 (43).

Supplementary Fig. 27 | Putative assignment of (**31**). **A)** Compound (**31**) [RRT 2.35 min] was putatively assigned as ψ-taraxasterol. RRT = Relative Retention Time (Relative to internal standard, coprostanol). **B)** EI spectrum for (**31**) (representative from this work). **C)** EI spectrum for compound (**29**) (representative from this work) confirmed as related isomer taraxasterol in analysis of OSC7 (Supplementary Fig. 10). Compound (**31**) was found to have a very similar EI spectrum and close retention time to compound (**29**) and the literature spectrum of taraxasterol (found in the supporting information of the reference)[14].

A

40 = RRT 5.31 min

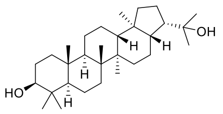

3β,20-dihydroxyhopane

B

EI spectrum of Compound (40) from analysis of OSC14

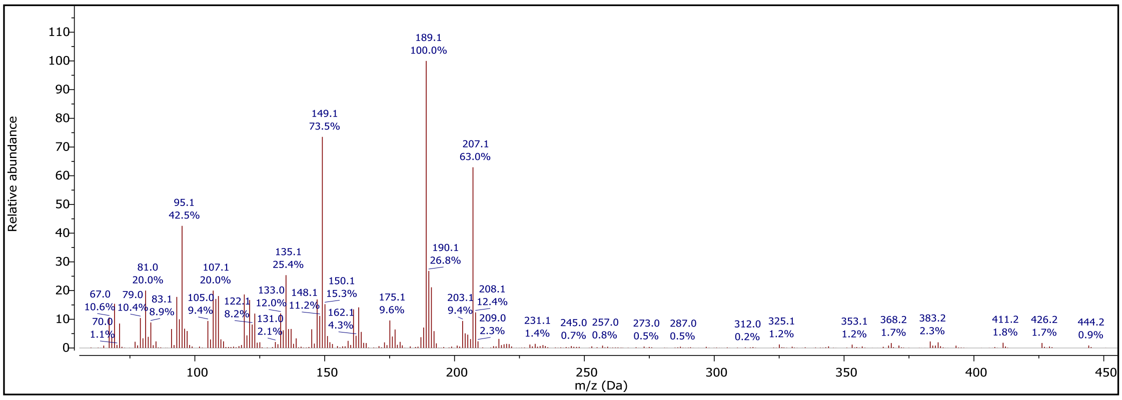

C

Detection of a fragment of 59 m/z using a wider mass range scan for compound (40) suggesting the second hydroxyl group is at C20

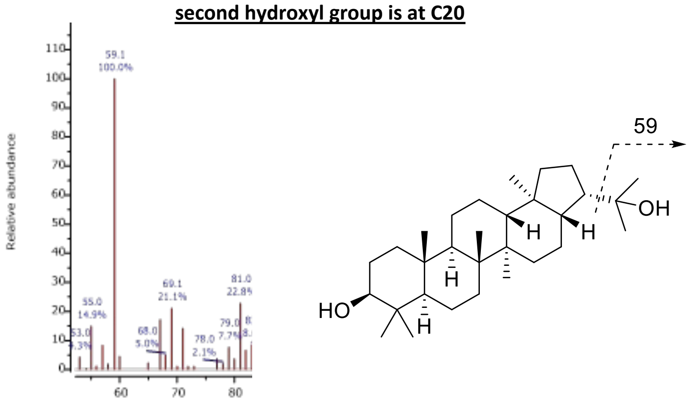

Supplementary Fig. 28 | Putative assignment of (**40**). **A)** Compound (**40**) [RRT 5.31 min] was putatively assigned as 3β,20-dihydroxyhopane. RRT = Relative Retention Time (Relative to internal standard, coprostanol). Compound (**40**) is produced by OSC14 which also produces hopanol B (**38**). **B)** Representative EI spectrum of compound (**40**) (from this work). The late retention time of (**40**) and its EI mass spectrum were consistent with a diol derivative of hopanol B compound (similar scaffold fragments ions to hopanol B but with a parent ion of 444). **C)** The EI spectrum was subsequently reacquired scanning a wider mass range, which allowed detection of a fragment of 59. This fragment suggested the second hydroxyl was at C20, because this would be consistent with fragmentation of the isopropane sidechain plus OH.

Carbon numbering scheme and selected COSY, HMBC

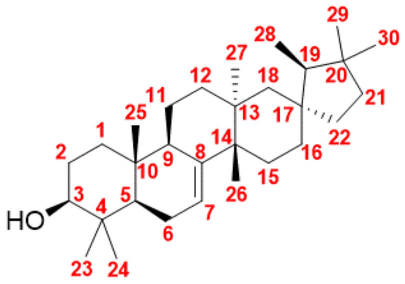

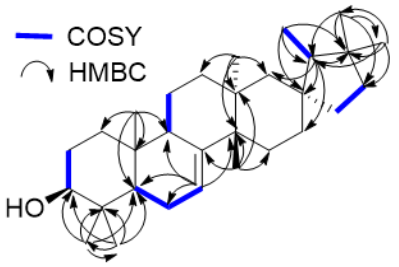

Carbon #	^{13}C δ (150 MHz)	^{1}H δ (600 MHz)	Carbon #	^{13}C δ (150 MHz)	^{1}H δ (600 MHz)
**8**	144.73	/	**13**	34.50	/
**7**	116.41	5.37 (1H, dd, *J*= 6.9, 3.2)	**12**	34.44	1.38 (1H, m)<br>1.34 (1H, m)
**3**	79.28	3.24 (1H, dd, *J*= 11.4, 4.0)	**30**	31.96	0.92 (3H, s)
**19**	55.41	1.09 (1H, m)	**15**	29.63	1.49 (1H, m)<br>1.26 (1H, m)
**5**	50.60	1.32 (1H, m)	**27**	27.73	1.03 (3H, s)
**18**	49.60	1.54 (1H, m)<br>0.78 (1H, m)	**2**	27.70	1.65 (1H, m)<br>1.59 (1H, m)
**9**	48.09	2.32 (1H, m)	**24**	27.54	0.97 (3H, s)
**17**	45.35	/	**29**	26.65	0.83 (3H, s)
**21**	40.44	1.49 (2H, m)	**16**	24.84	1.50 (1H, m)<br>1.22 (1H, m)
**14**	39.82	/	**6**	24.11	2.16 (1H, m)<br>1.98 (1H, m)
**4**	38.93	/	**26**	23.64	0.93 (3H, s)
**20**	37.89	/	**11**	16.45	1.57 (1H, m)<br>1.50 (1H, m)
**22**	37.46	1.83 (1H, m)<br>1.31 (1H, m)	**23**	14.65	0.86 (3H, s)
**1**	36.85	1.65 (1H, m)<br>1.11 (1H, m)	**25**	12.91	0.75 (3H, s)
**10**	35.33	/	**28**	8.96	0.78 (3H, d, *J*= 7.4)

Supplementary Fig. 29 | ^{13}C (150 MHz) & ^{1}H (600 MHz) δ assignments spirokalanchoeol (**24b**) $CDCl_3$ [referenced to TMS]. Assignments were made via a combination of ^{1}H, ^{13}C, COSY, DEPT-135, DEPT-edited HSQC, HMBC. Where signals overlap ^{1}H δ is reported as the centre of the respective HSQC crosspeak. Multiplicities are described as, s = singlet, d = doublet, dd = doublet of doublets, dt = doublet of triplets, t = triplet, q = quartet, quint = quintet, tquin = triplet of quintets, m = multiplet, br = broad, appt = apparent.

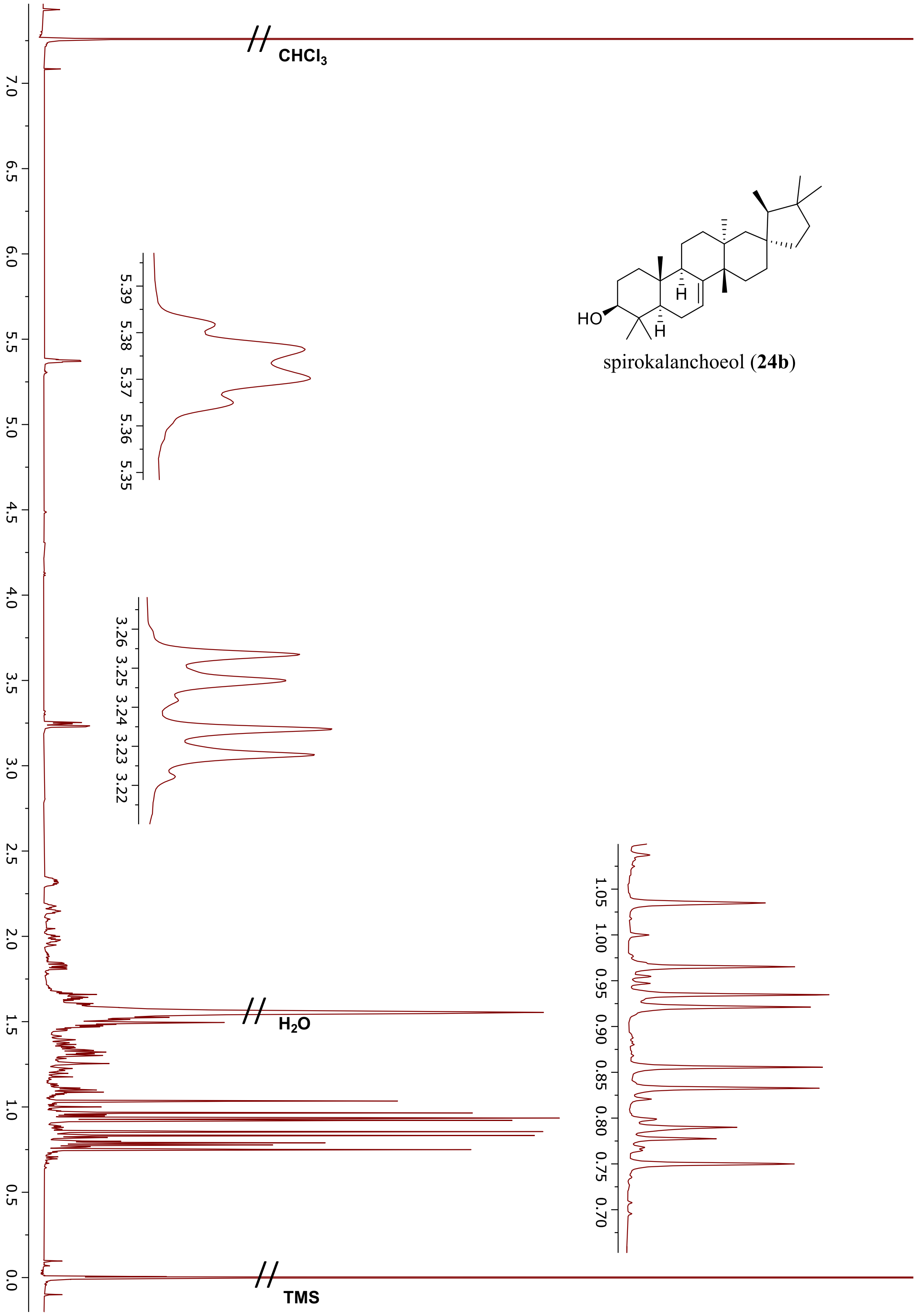

Supplementary Fig. 30 | ^{1}H spectrum spirokalanchoeol (**24b**) [600 MHz, $CDCl_3$ referenced to TMS]. Units: ppm.

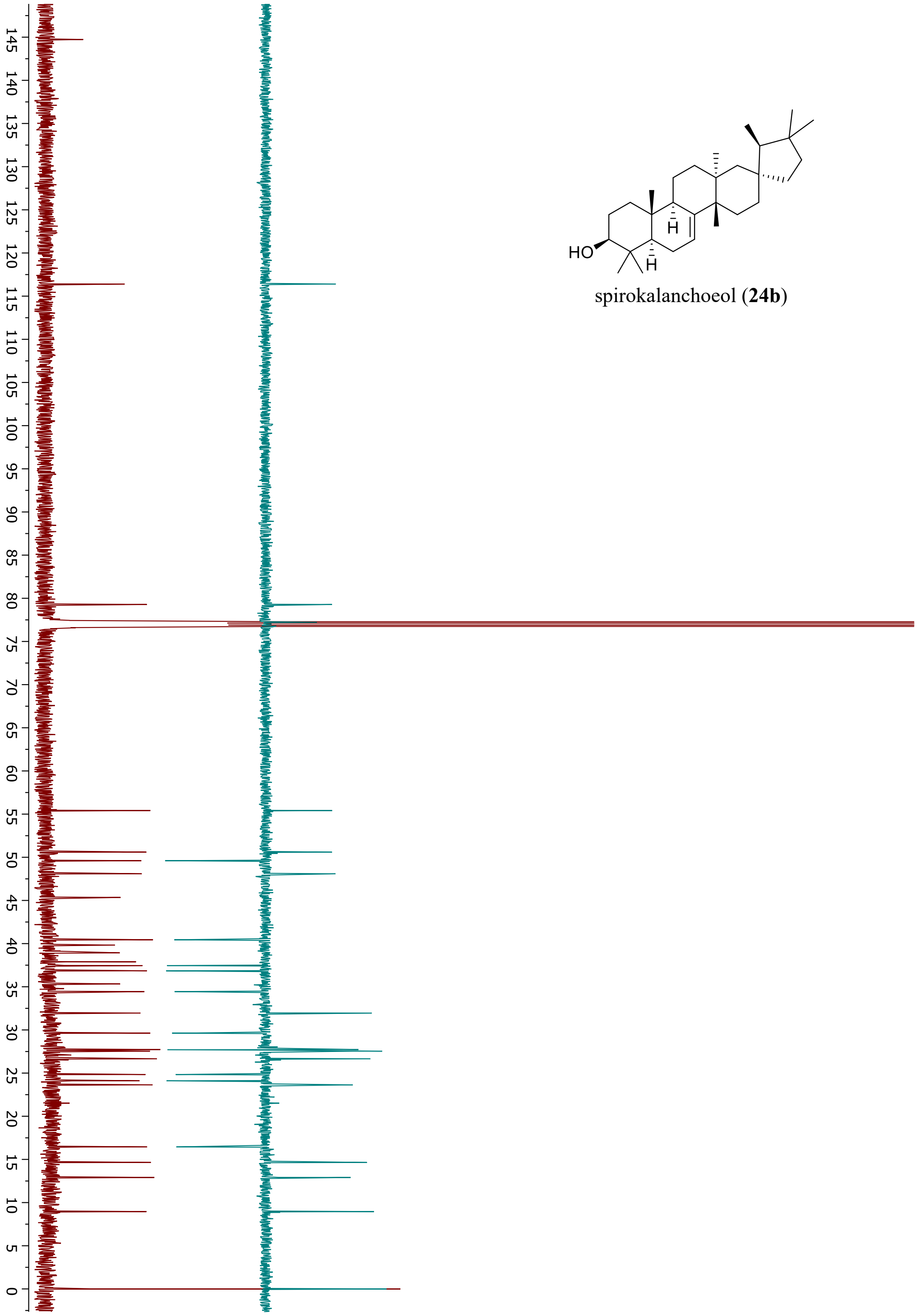

spirokalanchoeol (**24b**)

Supplementary Fig. 31 | ^{13}C & DEPT-135 spectrum spirokalanchoeol (**24b**) [150 MHz, $CDCl_3$ referenced to TMS]. Units: ppm.

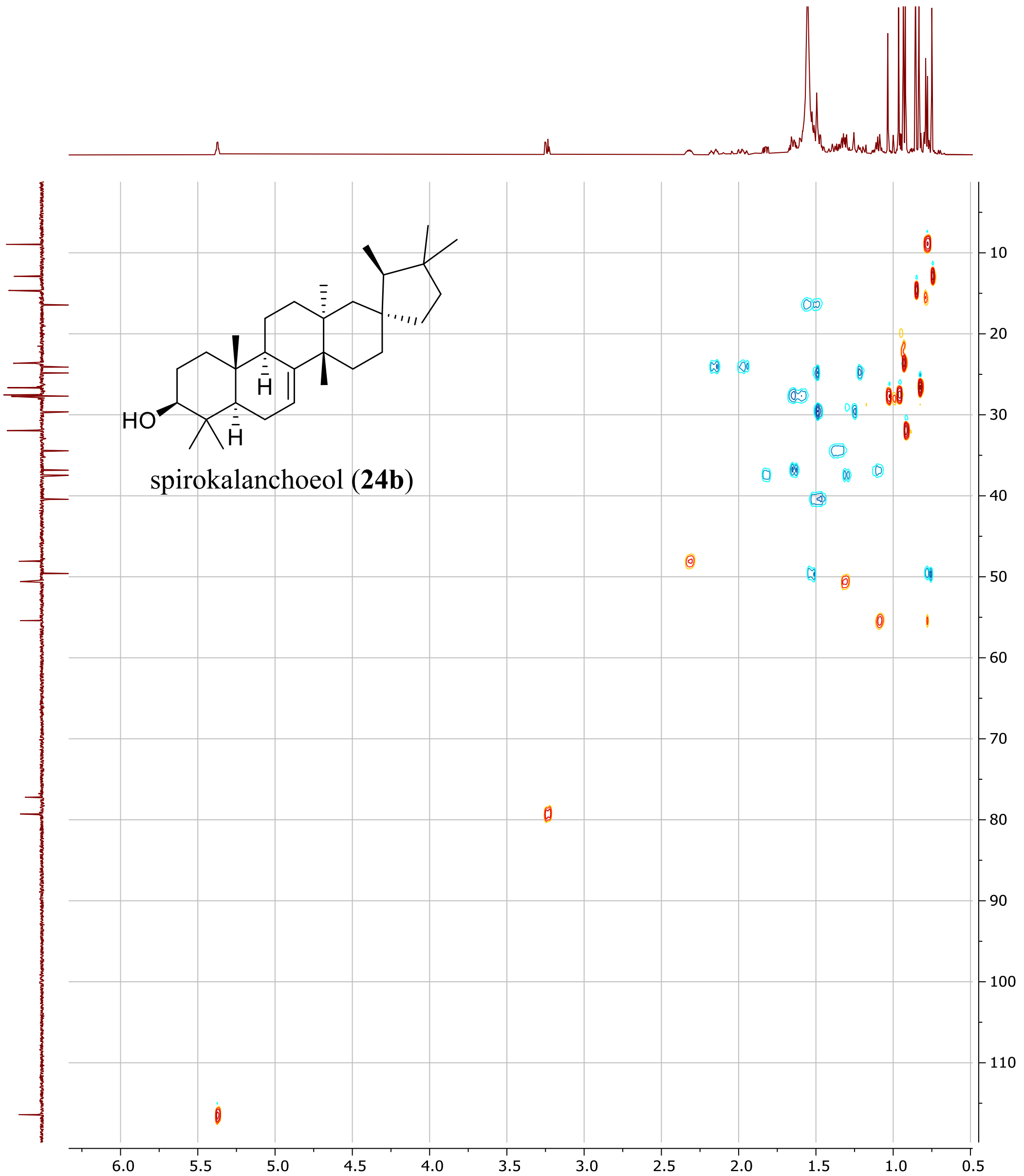

Supplementary Fig. 32 | DEPT-edited-HSQC spectrum spirokalanchoeol (**24b**) [^{1}H 600 MHz, ^{13}C 150 MHz, $CDCl_3$ referenced to TMS] Units: ppm.

Carbon numbering scheme and selected COSY, HMBC

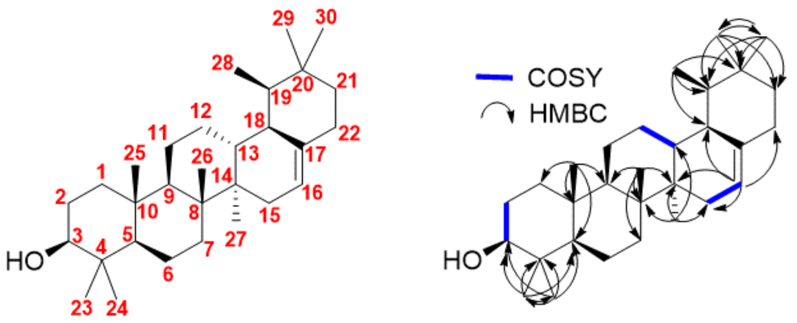

Carbon #	^{13}C δ (150 MHz)	^{1}H δ (600 MHz)	Carbon #	^{13}C δ (150 MHz)	^{1}H δ (600 MHz)
**17**	135.03	/	**21**	33.11	1.42 (1H, m)<br>1.08 (1H, m)
**16**	120.55	5.35 (1H, m)	**15**	31.53	2.08 (1H, m)<br>1.42 (1H, m)
**3**	79.02	3.21 (1H, dd, *J*= 11.5, 4.8)	**22**	30.78	2.14 (1H, m)<br>1.98 (1H, m)
**5**	55.36	0.71 (1H, m)	**30**	30.57	0.89 (3H, s)
**9**	50.72	1.21 (1H, m)	**24**	28.03	0.98 (3H, s)
**18**	41.34	1.91 (1H, m)	**2**	27.40	1.62 (1H, m)<br>1.57 (1H, m)
**8**	40.31	/	**29**	26.46	1.03 (3H, s)
**14**	40.18	/	**11**	26.28	1.69 (1H, m)<br>0.94 (1H, m)
**19**	40.05	1.47 (1H, m)	**12**	20.89	1.47 (1H, m)<br>1.28 (1H, m)
**4**	38.88	/	**6**	18.15	1.52 (1H, m)<br>1.39 (1H, m)
**1**	38.83	1.72 (1H, m)<br>0.95 (1H, m)	**25**	16.39	0.85 (3H, s)
**10**	37.16	/	**26**	16.23	0.96 (3H, s)
**13**	34.99	1.66 (1H, m)	**23**	15.44	0.77 (3H, s)
**7**	34.21	1.46 (1H, m)<br>1.37 (1H, m)	**27**	14.05	0.83 (3H, s)
**20**	33.82	/	**28**	9.78	0.70 (3H, d, *J*= 6.9)

Supplementary Fig. 33 | ^{13}C (150 MHz) & ^{1}H (600 MHz) δ assignments of kalanchoeol (**24a**) $CDCl_3$ [referenced to TMS]. Assignments were made via a combination of ^{1}H, ^{13}C, COSY, DEPT-135, DEPT-edited HSQC, HMBC. Where signals overlap ^{1}H δ is reported as the centre of the respective HSQC crosspeak. Multiplicities are described as, s = singlet, d = doublet, dd = doublet of doublets, dt = doublet of triplets, t = triplet, q = quartet, quint = quintet, tquin = triplet of quintets, m = multiplet, br = broad, appt = apparent.

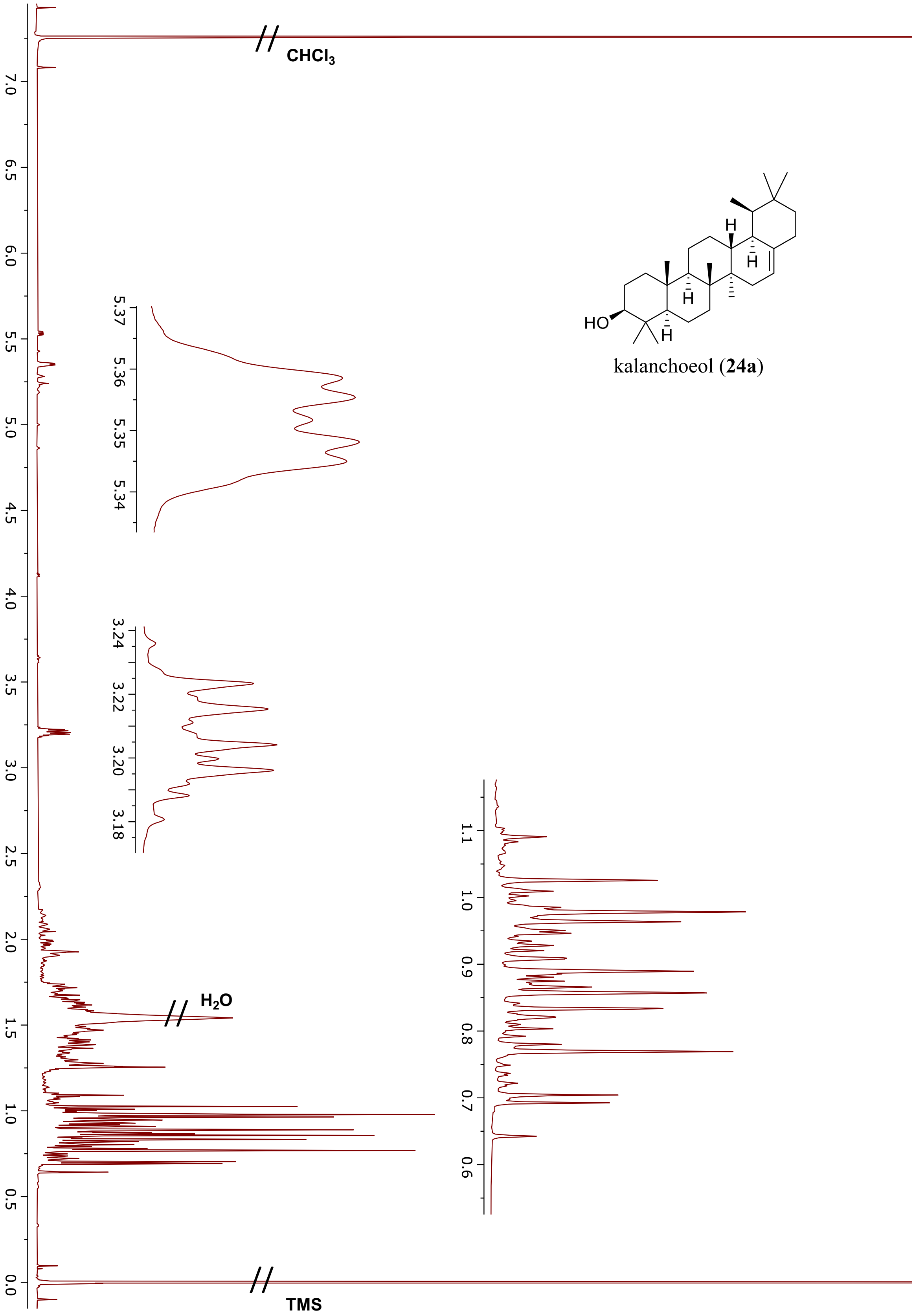

Supplementary Fig. 34 | ^{1}H spectrum kalanchoeol (**24a**) [600 MHz, $CDCl_3$ referenced to TMS]. Units: ppm.

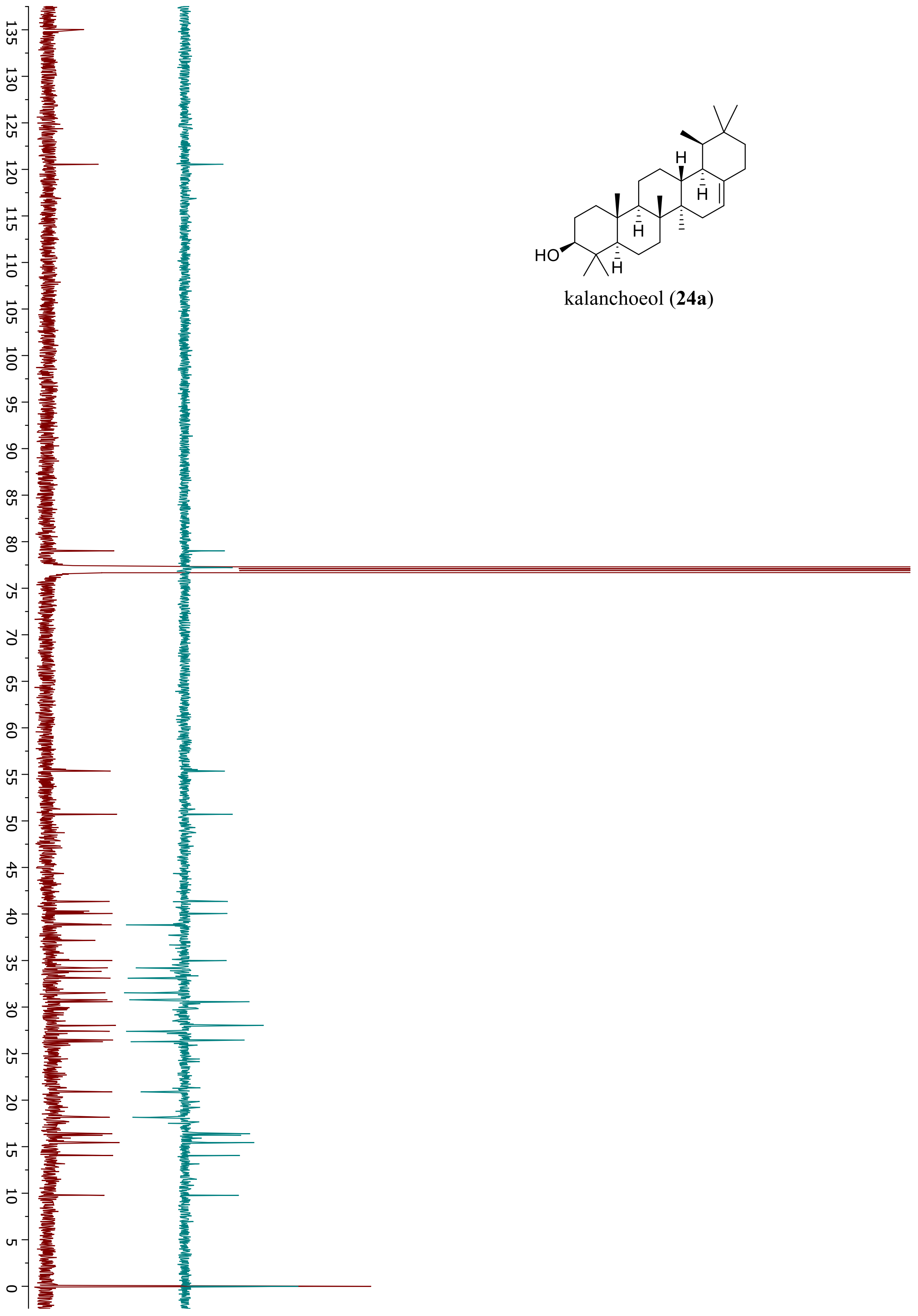

Supplementary Fig. 35 | ^{13}C & DEPT-135 spectrum kalanchoeol (**24a**) [150 MHz, $CDCl_3$ referenced to TMS]. Units: ppm.

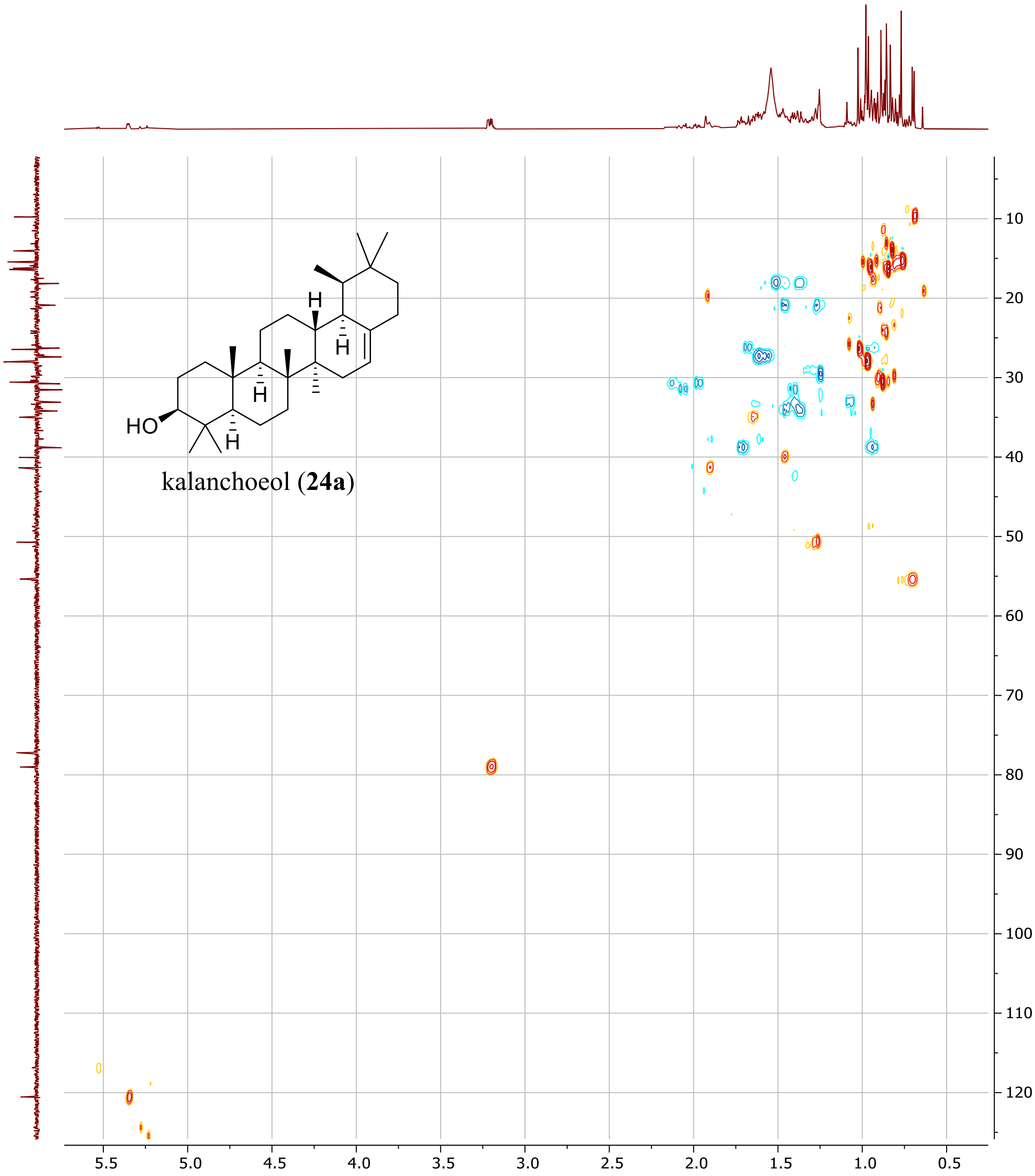

Supplementary Fig. 36 | DEPT-edited-HSQC spectrum kalanchoeol (**24a**) [^{1}H 600 MHz, ^{13}C 150 MHz, $CDCl_3$ referenced to TMS] Units: ppm.

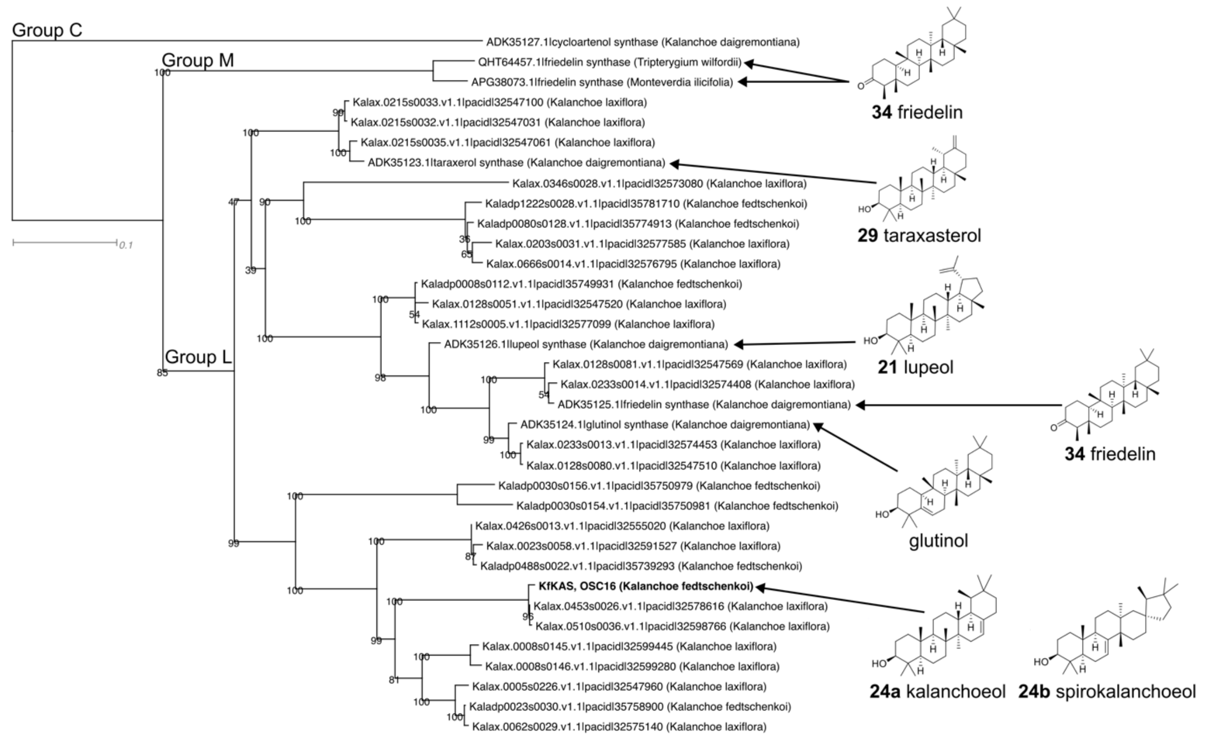

Supplementary Fig. 37 | Phylogeny of OSC16 (KfKAS). The NCBI non-redundant database was searched via BLASTP with OSC16, and the top 1000 hits were aligned with the mined Group L OSCs and characterised OSC sequences. A draft phylogeny was generated (with MAFFT version 7.394[15] and FastTree version 2.1[16]), but no further sequences were found that fell into Group L, nor was any sequence found with >75% sequence identity. The above tree was generated in RaXML (version 8.2[17]) from an alignment of Group L sequences, the characterised friedelin synthases in Group M and a Kalanchoe CAS as an outgroup. The products of OSC16 can be rationalised as being biosynthetically related to the other products produced by OSCs within this Group M clade (see Fig. 1 and Fig. 5).

```
                                         OSC9 MWKLKIGEG--GGPYIYSTNNYAGRQIWEYDPNAGTPEEQEAFEKARKGFTKQRQKGPHT   58
                   tirucallol synthase (CrOSC) MWRLKIGAKAAGDPYLSTTNNYVGKQVWEFDPEAGSPEELAEVEHARKNFSANRSQY-KA   59
tirucalla-7,24-diene-3β-ol synthase (AiOSC1) MWKLKIAEGDKNSPYISTTNNFVGRQIWEFDPNAGTAEELAEVEEARQNFYKNRHQV-KP   59
                                              **:***.     ..**: :***:.*:*:**:**:**: **   .*.**:.*  :* :  :

                                         OSC9 CGDLFMRMQMIKESEVDVLSIPPVRLREEEEVNYEAITTAVRKAVRLNCALQAKDGHWPA   118
                   tirucallol synthase (CrOSC) SADLLWRMQFLREKNFE-QKIPRVIIENAEKITYEDANTALKRGIHYMAALQSDDGHWPA   118
tirucalla-7,24-diene-3β-ol synthase (AiOSC1) ASDLIFRLQFLREKNFK-QTIPQVKVEDGEEITYDTATAAMKRAAHYFSAIQASDGHWPA   118
                                              ..**: *:*:::*.:..  .** * :.: *::.*:  .:*:::. :  .*:*:.******

                                         OSC9 ENSGPLFFTPPLMIALYISGSIDMILTSEHKKELVRYLYNHQNEDGGWGLYIDGHSTMIG   178
                   tirucallol synthase (CrOSC) ENAGCMFFNAPFVICLYITGHLDKIFSLEHRKEMLRYLYIHQNEDGGWGIDVESHSFMFC   178
tirucalla-7,24-diene-3β-ol synthase (AiOSC1) ENSGPMYFLPPFVFCLYITGHLDTVFTAAHRREVLRYLYNHQHEDGGWGIHIEAPSSMFG   178
                                              **:* ::*  *:::.***:* :* :::  *::*::**** **:******: ::. * *:

                                                                                  216
                                         OSC9 SALNYVALRLLGEEAD-----DAIARGRNWILQHGGATAIPSWGKIYLSVLGVYEWDGCN   233
                   tirucallol synthase (CrOSC) TVINYICLRIFGVDPDHDGKTSACARARKWIIDHGGATGTPLFGKAWLSVLGVYDWSGCR   238
tirucalla-7,24-diene-3β-ol synthase (AiOSC1) TVYSYLTMRLLGLGPN-DGENNACARARKWIRDNGGVTYIPSWGKNWLSILGLFEWAGTH   237
                                              :. .*: :*::*   :      .* **.*:** ::**.*  * :** :**:**:::* * .

                                                          254 256
                                         OSC9 PIPPEFWLFPSHFPFHPAKMWNYCRTTYMPMSYLYAKRYHGPLTNLVLSIRNEIHVKPYD   293
                   tirucallol synthase (CrOSC) PILPEFWFLPSYFPVNGGTLWIYLRDTFMAISYLFGKKFVATTTPLILQLREEIYPEVYN   298
tirucalla-7,24-diene-3β-ol synthase (AiOSC1) PMPPEFWMLPSHFPLHPAQMWCFCRLVYMPLCYLYGKRFVGPITPLIKQLREELHTEPYD   297
                                              *: ****::**:**.: . :* : * .:* :.**:.*:: .  * *: .:*:*:: : *:

                                         OSC9 QIDWNNTRMACCKEDTYYPHTFIQDVLWNALQYCTEPIMRRWPFNK-IREMAMRKVIMHM   352
                   tirucallol synthase (CrOSC) EIDWSKARHCCAKEDLYYPQSFIQDLFWNSVHIFSEKIVNRWPFNKFIREKAVQKAMDLI   358
tirucalla-7,24-diene-3β-ol synthase (AiOSC1) KINWRKVRHQCAKTDLYYPHPFVQEVLWDTLYFATEPLLTRWPLNKYVREKALKQTMKII   357
                                              :*:* :.*  *.* * ***: *:*:::*:::   :* :: ***:** :** *:::.:  :

                                         OSC9 RYGAEESRYITIGAIEKSLQMMCWWAEDPNCDELKYHLARVPDFLWLAEDGMTMQTFGSQ   412
                   tirucallol synthase (CrOSC) HYHDEATKYVTGGAVPKAFHMLACWVEDPESDYFKKHLAQVPGYIWIAEDGLKIQTFGSQ   418
tirucalla-7,24-diene-3β-ol synthase (AiOSC1) HYEDQSSRYITIGCVEKPLCMLACWVEDPEGVAFKKHLERIADFIWIGEDGMKVQTFGSQ   417
                                              :*  : ::*:* *.: * : *:. *.***:   :* ** :: .::*:.***:.:******

                                         OSC9 NWDATFATQALISSGMVEEYGDCLKKAHFYLKQSQIKENPKGDFKSMYRHFSKGAWAFSD   472
                   tirucallol synthase (CrOSC) LWDTPFMLQVMLAADVDEEIKSTLIKGYSFLKNSQFTEDPPGDFVKMFRDRSKGGWGFSD   478
tirucalla-7,24-diene-3β-ol synthase (AiOSC1) TWDTALGLQALLACNIVDEIGPALAKGHDYLKKAQVRDNPVGDYTSNFRHFSKGAWTFSD   477
                                               **: :  *.:::..: :*    * *.: :**::*. ::* **: . :*. ***.* ***

                                         OSC9 QDHGWVVSDCTAEAIKCLLLLGQMSPEIAGEKVDVQCLYEAVDICLYLQSPESGGFAVWE   532
                   tirucallol synthase (CrOSC) KDQGWFVSDCTSESLECCLIFETMPTEFIGEKMDVERLYEAVDMLLYMQSK-NGGISIWE   537
tirucalla-7,24-diene-3β-ol synthase (AiOSC1) QDHGWQVSDCTAESLKCCLHFSMLPPEIVGEKHDPERLYEAVNFILSLQDK-NGGIAVWE   536
                                              :*:** *****:*:::* * :  :  *: *** * : *****:: * :*.  .**:::**

                                                                     548 549         561
                                         OSC9 PPIPQPYLEMLNPSELFADIVVQGEHVETTGSIVSALAAFKHDYPDYRAKEVAMSIAKAA   592
                   tirucallol synthase (CrOSC) VESGKKWLEWLSPIEFIEDTILEHEYLECTGSAVVVLVRFLKQHPEYRQKEVQRFITKGV   597
tirucalla-7,24-diene-3β-ol synthase (AiOSC1) KAGASLMLEWLNPVEFLEDLIVEHTYVECTASAIEAFVMFKKLYPHHRKKEIENFLVKAV   596
                                                   .   ** *.* *:: * :::  ::* *.* : .:. * : :*.:* **:   :.*..

                                         OSC9 EYLETQQQADGSWYGYWGICFLYGTCFALLGLTAAGRSYENSEAIRKAAHFFLSKQNQEG   652
                   tirucallol synthase (CrOSC) EYIESKQTPDGSWFGNWGVCFIPGTFFAIRGLVAADKTYENCEAIRRAVQFILRTQNVEG   657
tirucalla-7,24-diene-3β-ol synthase (AiOSC1) QYIENEQTADGSWYGNWGVCFLYGTCFALGGLHAAGKTYNNCLAIRRAVEFLLQAQSDDG   656
                                              :*:*.:*  ****:* **:**: ** **: ** **.::*:*. ***:*..*:*  *. :*

                                         OSC9 GWGECLESCPTMKYIPFEGNRSNVVQTSWAMLGLMYTEQAERDPTPLHRAAKLLINAQME   712
                   tirucallol synthase (CrOSC) GWGESYLSCPKKMYIPLEGNKSNIVNTGQALIILILSGQMDRDPSPVHRGAKVLINSQMD   717
tirucalla-7,24-diene-3β-ol synthase (AiOSC1) GWGESYKSCPSKIYVPLDGKRSTVVHTALAILGLIHAGQAERDPTPIHRGVKLLINSQLE   716
                                              ****.  ***.  *:*::*::*.:*:*. *:: *: : * :***:*:**..*:***:*::

                                                           724 725
                                         OSC9 DGDFPQQDITGSYMKNCMLHYASHRSYFPLMALSEYRKRVWIS-   755
                   tirucallol synthase (CrOSC) NGDFPQEDIRGVYKNNVLLHYPTYRNIYSLWALIYYSKALRLLH   761
tirucalla-7,24-diene-3β-ol synthase (AiOSC1) NGDFPQQEIMGVFMRNCMLHYAQYRNIFPLWALAEYRRKVPLPN   760
                                              :*****::* * * : .* :***  :*. : * **  * : : :
```

Supplementary Fig. 38 | Multiple sequence alignment of the amino acid sequences for OSC9 and functional relatives. Alignment generated using CLUSTAL O (1.2.4) of OSC9 (from plant species *Ipomoea nil*; this study), a tirucallol synthase from *Capsella rubella* (CrOSC)[1] and a tirucalla-7,24-diene-3β-ol synthase from *Azadirachta indica* (AiOSC1)[9]. The residues studied are highlighted and colour-coded to match Fig. 8B.

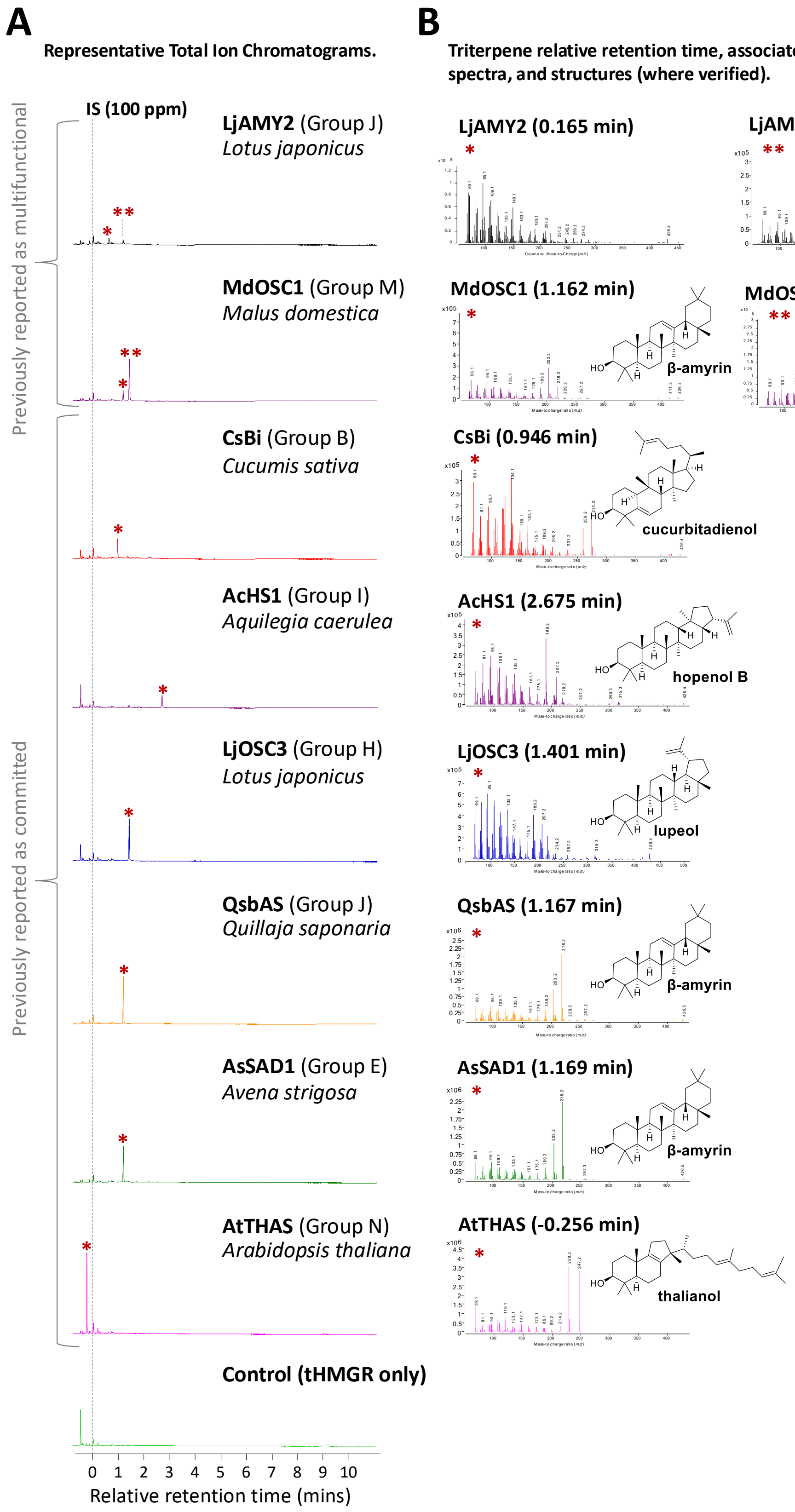

Supplementary Fig. 39 | Functional analysis of 8 previously characterised OSCs using the workflow in this paper. **A)** Representative Total Ion Chromatograms for each OSC (LjAMY2[18], MdOSC1[19], CsBi[7], AcHS1[20], LjOSC3[21], QsbAS[22], AsSAD1[23] and AtTHAS[24]) and the control (tHMGR expressed alone) scaled to the internal standard. Retention times are reported relative to the internal standard. Red asterisks label OSC triterpene products. IS = internal standard (coprostanol 100 ppm). **B)** Triterpene retention time (relative to the internal standard), associated EI mass spectra, and reported structure.

Supplementary Table 1 | Summary of selected OSCs from genome mining detailed this study and their products.

ID	Name	Genbank	Species	Product(s)	Reason for selection	Notes on activity	OSC Group
OSC1	AaCBS1	PV548881	*Aquilaria agallochum*	cucurbitadienol [6]; and 3 minor products [23, 33, 35]	Conserved clade but from a non-cucurbit species	First cucurbitadienol activity outside the Cucurbitales	B
OSC2	EgDDS1	PV548882	*Elaeis guineensis*	dammarenediol-II [36]; dammara-13(18),24-dien-3β-ol-like [4], eupha-7,24-dien-3β-ol [11]; and 3 minor products [14, 25, 27]	Unique OSC between monocot protosteryl- and dammarenyl-derived triterpenes	Producing dammarenyl-derived tetracycles, primarily dammarenediol-II representing direct quenching of the dammarenyl cation	E
OSC3	-	-	*Zoysia japonica*	*None observed*	Sub-clade of monocot triterpene synthases with no characterised members	*None observed*	E
OSC4	AoPPS1	PV548883	*Asparagus officinalis*	(9R,10S)-polypoda-8(26),13E,17E,21-tetraen-β-ol [17]	Stem member of unique monocot clade previously only known in Poales, with only one characterised member	First poaceatapetol-like activity outside the Poales	G
OSC5	-	-	*Sorghum bicolor*	*None observed*	Conserved clade with only one characterised member	*None observed*	G
OSC6	JrOSC1	PV548884	*Juglans regia*	bauerenol [28]; α-amyrin [20]; β-amyrin [13]	Duplication and diversification within the clade, potentially indicative of new function	New activity within a clade previously believed to be functionally conserved	H
OSC7	InOSC1	PV548885	*Ipomoea nil*	taraxasterol [29]; lupeol [21]; β-amyrin [13]; 3β,20-dihydroxylupane [39]; ψ-taraxasterol [31]; and minor product [15]	Duplication and diversification within the clade, potentially indicative of new function	New activity within a clade previously believed to be functionally conserved	H
OSC8	GbAAS1	PV548886	*Gossypium bardense*	achilleol A-like [1]	Unknown what functions this clade has for anything outside of asterids	Rare second example of a committed monocyclic triterpene synthase (with BrAAS1)	I
OSC9	InOSC2	PV548887	*Ipomoea nil*	euphol [5]; tirucallol [7]; tirucalla-7,24-diene-3β-ol [18]; eupha-7,24-dien-3β-ol [11]	Clade is source for all OSC diversity outside of core clade for the asterids, with large diversification apparent	Tetracyclic triterpene synthase producing triterpenes of both the euphane and tirucallane classes in approximately equal amounts	I
OSC10	DcOSC1	PV548888	*Daucus carota*	β-amyrin [13]; α-amyrin [20]; ψ-taraxasterol [31]; taraxasterol [29]; and minor product [12]	Clade is source for all OSC diversity outside of core clade for the asterids, with large diversification apparent	Producing a diverse range of closely related 6,6,6,6,6 pentacyclic triterpenes	I
OSC11	PbFRS1	PV548889	*Pyrus x bretschneideri*	friedelin [34]	Duplication and diversification, many Rosaceae have duplications here, most divergent and expressed	New example of a committed friedelin synthase phylogenetically distance from previously reported examples	J
OSC12	VrOSC1	PV548890	*Vigna radiata*	α-amyrin [20]; β-amyrin [13]; taraxasterol [29]	Duplication and clear diversification away from bAS shared amongst many Fabales	Mixed amyrin synthase producing primarily just α-amyrin and β-amyrin (with AaOSC1 & HlOSC1)	J
OSC13	-	-	*Aquilaria agallochum*	*None observed*	Duplication and diversification in core clade	*None observed*	J
OSC14	CbHDS1	PV548891	*Capsella bursa-pastoris*	hopanol B [38]; hopena-3,20-diol [40]; and minor products [2, 3, 14, 19]	Duplication and diversification in a clade with diverse functions	Producing 6,6,6,6,5 hopanoid-type triterpenes	K
OSC15	AaOSC1	PV548892	*Arabis alpina*	α-amyrin [20]; β-amyrin [13]	Duplication and diversification in a clade with diverse functions	Mixed amyrin synthase producing just α-amyrin and β-amyrin (with VrOSC1 & HlOSC1)	K
OSC16	KfKAS1	PV548893	*Kalanchoe fedtschenkoi*	kalanchoeol [24a]; spirokalanchoeol[24b]; eupha-7,24-dien-3β-ol [11]; α-amyrin [20] and 9 minor products [8, 9, 10, 16, 22, 26, 30, 31, 37]	Unknown sub-clade of a group with known diverse producers of dammerneyl derivatives	Produces novel products (kalanchoeol [24a]; spirokalanchoeol[24b])	L
OSC17	HlOSC1	PV548894	*Humulus lupulus*	α-amyrin [20]; β-amyrin [13]	Divergent clade, unknown function	Mixed amyrin synthase producing just α-amyrin and β-amyrin (with VrOSC1 & AaOSC1)	M
OSC18	-	-	*Citrullus lanatus*	*None observed*	Divergent clade, curcurbits known to produce triterpenes	*None observed*	M
OSC19	ThDHS1	PV548895	*Tarenaya hassleriana*	3β,20-dihydroxylupane [39]	Duplication and diversification in a clade with diverse functions	Parent OSC of orphan scaffold (first committed 3β,20-dihydroxylupane synthase)	N
OSC20	BrAAS1	PV548896	*Brassica rapa*	achilleol A-like [1]	Duplication and diversification in a clade with diverse functions	Rare second example of a committed monocyclic triterpene synthase (with GbAAS1)	N

Supplementary References

1. Liu, Z. et al. Drivers of metabolic diversification: how dynamic genomic neighbourhoods generate new biosynthetic pathways in the Brassicaceae. *New Phytologist* **227**, 1109-1123 (2020).

2. Thimmappa, R. et al. Triterpene biosynthesis in plants. *Annual Review of Plant Biology* **65**, 225-257 (2014).

3. Kushiro, T., Shibuya, M., Masuda, K. & Ebizuka, Y. Mutational studies on triterpene synthases: engineering lupeol synthase into β-amyrin synthase. *Journal of the American Chemical Society* **122**, 6816-6824 (2000).

4. Carothers, S. et al. Bauerenol acetate, the pentacyclic triterpenoid from Tabernaemontana longipes, is an antitrypanosomal agent. *Molecules* **23**, 355 (2018).

5. Reed, J. et al. A translational synthetic biology platform for rapid access to gram-scale quantities of novel drug-like molecules. *Metabolic Engineering* **42**, 185-193 (2017).

6. Reynolds, W.F. et al. Total assignment of ^{13}C and ^{1}H spectra of three isomeric triterpenol derivatives by 2D NMR: an investigation of the potential utility of 1H chemical shifts in structural investigations of complex natural products. *Tetrahedron* **42**, 3419-3428 (1986).

7. Shang, Y. et al. Biosynthesis, regulation, and domestication of bitterness in cucumber. *Science* **346**, 1084-1088 (2014).

8. Salmon, M. et al. A conserved amino acid residue critical for product and substrate specificity in plant triterpene synthases. *Proceedings of the National Academy of Sciences* **113**, E4407-E4414 (2016).

9. Hodgson, H. et al. Identification of key enzymes responsible for protolimonoid biosynthesis in plants: Opening the door to azadirachtin production. *Proceedings of the National Academy of Sciences* **116**, 17096-17104 (2019).

10. Fukuda, Y., Watanabe, T. & Hoshino, T. Mutated variants of squalene-hopene cyclase: enzymatic syntheses of triterpenes bearing oxygen-bridged monocycles and a new 6,6,6,6,6-fusded pentacyclic scaffold, named neogammacerane, from 2,3-oxidosqualene. *Organic & Biomolecular Chemistry* **16**, 8365-8378 (2018).

11. Ideno, N. et al. Alicyclobacillus acidocaldarius squalene-hopene cyclase: The critical role of steric bulk at ala306 and the first enzymatic synthesis of epoxydammarane from 2,3-oxidosqualene. *ChemBioChem* **19**, 1873-1886 (2018).

12. Wu, T.-K., Wang, T.-T., Chang, C.-H., Liu, Y.-T. & Shie, W.-S. Importance of Saccharomyces cerevisiae oxidosqualene-lanosterol cyclase tyrosine 707 residue for chair-boat bicyclic ring formation and deprotonation reactions. *Organic Letters* **10**, 4959-4962 (2008).

13. Meksuriyen, D., Dhammika Nanayakkara, N.P., Phoebe, C.H. & Cordell, G.A. Two triterpenes from Davidsonia pruriens. *Phytochemistry* **25**, 1685-1689 (1986).

14. Gadouche, L. et al. Triterpenoid and steroid content of lipophilic extracts of selected medicinal plants of the mediterranean region. *Molecules* **28**, 697 (2023).

15. Katoh, K. & Standley, D.M. MAFFT multiple sequence alignment software version 7: improvements in performance and usability. *Molecular Biology and Evolution* **30**, 772–780 (2013).

16. Price, M.N., Dehal, P.S., & Arkin, A.P. FastTree 2 -- approximately maximum-likelihood trees for large alignments. *PLoS ONE* **5**(3), e9490 (2010).

17. Stamatakis, A. RAxML version 8: a tool for phylogenetic analysis and post-analysis of large phylogenies. *Bioinformatics* **30**, 1312–1313 (2014).

18. Iturbe-Ormaetxe, I., Haralampidis, K., Papadopoulou, K., & Osbourn, A. E. Molecular cloning and characterization of triterpene synthases from Medicago truncatula and Lotus japonicus. *Plant molecular biology*, **51**(5), 731–743 (2003).

19. Brendolise, C., Yauk, Y. K., Eberhard, E. D., Wang, M., Chagne, D., Andre, C., Greenwood, D. R., & Beuning, L. L. An unusual plant triterpene synthase with predominant α-amyrin-producing activity identified by characterizing oxidosqualene cyclases from Malus × domestica. *The FEBS journal*, **278**(14), 2485–2499 (2011).

20. Liang, M., Zhang, F., Xu, J., Wang, X., Wu, R., & Xue, Z. A conserved mechanism affecting hydride shifting and deprotonation in the synthesis of hopane triterpenes as compositions of wax in oat. *Proceedings of the National Academy of Sciences of the United States of America*, **119**(12), e2118709119 (2022).

21. Sawai, S., Shindo, T., Sato, S., Kaneko, T., Tabata, S., Ayabe, S. I., & Aoki, T. Functional and structural analysis of genes encoding oxidosqualene cyclases of Lotus japonicus. *Plant Science*, **170**(2), 247-257 (2006).

22. Reed, J. et al. Elucidation of the pathway for biosynthesis of saponin adjuvants from the soapbark tree. *Science*, **379**(6638), 1252–1264 (2023).

23. Haralampidis, K., Bryan, G., Qi, X., Papadopoulou, K., Bakht, S., Melton, R., & Osbourn, A. A new class of oxidosqualene cyclases directs synthesis of antimicrobial phytoprotectants in monocots. *Proceedings of the National Academy of Sciences of the United States of America*, **98**(23), 13431–13436 (2001).

24. Fazio, G. C., Xu, R., & Matsuda, S. P. Genome mining to identify new plant triterpenoids. *Journal of the American Chemical Society*, **126**(18), 5678–5679 (2004).
